# Investigation of salivary microRNAs as non-invasive biomarkers for weaning stress in piglets: A preliminary study

**DOI:** 10.1371/journal.pone.0360727

**Published:** 2026-10-13

**Authors:** Ga-Hyeon Jeong, Sung-Jea Park, Kyu-Sang Lim

**Affiliations:** Department of Animal Resources Science, Kongju National University, Yesan, Chungnam, Republic of Korea; Colorado State University College of Veterinary Medicine and Biomedical Sciences, UNITED STATES OF AMERICA

## Abstract

Weaning exposes piglets to abrupt nutritional and environmental changes that induce physiological stress. Saliva, unlike serum or tissue samples, enables repeated and stress-free sampling, making it a practical biological matrix for monitoring early-life adaptation. To evaluate whether salivary microRNAs (miRNAs) can serve as non-invasive indicators of these responses, we profiled miRNA expression in saliva collected from suckling and weaning piglets. Small RNA sequencing was conducted to detect salivary miRNAs and a total of 41 differentially expressed miRNAs (DEMs) were detected between the two groups, with 20 showing highly expression and 21 showing lowly expression in weaning piglets. Several of these DEMs have been previously associated with immune activity, inflammatory regulation, oxidative stress responses, and disease processes, highlighting their biological relevance during the weaning transition. Functional annotation of predicted target genes revealed enrichment in pathways such as TGF-beta, HIF-1, and AMPK signaling, as well as autophagy-related processes. These pathways are known to play key roles in stress adaptation, cellular metabolism, and immune modulation. These findings suggest that salivary miRNA profiles may reflect molecular changes associated with the weaning transition and may serve as candidate non-invasive indicators of physiological stress and health status in piglets. This approach may provide a basis for future development of saliva-based monitoring strategies after validation in larger cohorts and cost-effective targeted assays.

## Introduction

Weaning is the process in which piglets are separated from the sow and begin to consume solid feed, performing independent physiological functions. This process is typically conducted early in commercial farming to enhance productivity and efficiency. Although it optimizes the sow reproductive cycle and increases the economic efficiency of the farming environment [1], it can induce significant physiological and psychological stress in piglets [2]. Weaning stress not only involves the abrupt separation of piglets from the sow, but also requires them to adapt to a new environment, which includes changes in diet, social environment, and physical surroundings [3,4]. These factors negatively affect piglets, leading to disruptions in immune function, small intestine health, and growth. Specifically, an imbalance in the small intestinal microbiota can trigger inflammatory responses in the intestinal mucosa, impairing the small intestinal barrier function [5]. Additionally, the immune system may be weakened and oxidative stress levels may increase [6]. These physiological changes reduce the digestive capacity and compromise nutrient absorption in piglets, ultimately leading to decreased feed intake, diarrhea, and growth retardation [7,8]. Such alterations in small intestinal function profoundly impact the overall physiological stability of piglets and are directly linked to reduced productivity. Therefore, mitigating weaning stress has emerged as a critical research topic for improving piglet health and productivity.

Recently, microRNAs (miRNAs) have attracted attention for their crucial roles in various physiological processes, particularly as biological markers in stress responses and immune regulation [9–11]. MiRNAs are small non-coding RNAs [12] that regulate gene expression by binding to the 3’-untranslated region (UTR) of target mRNAs, leading to mRNA degradation or translation inhibition [13]. In relation to weaning stress, previous studies have focused on the effects of miRNAs on small intestine metabolism, stress responses, and immune function [14]. Studies have reported that miRNA expression patterns in the small intestine are altered by weaning stress, and these changes play a vital role in regulating digestion, immune functions, and stress responses in piglets [15,16]. Furthermore, miRNAs exhibit differential expression in the serum [17], making them valuable indicators reflecting the overall physiological response of piglets in real-time.

Although miRNA expression in the serum and small intestine have been analyzed extensively, recent studies have highlighted the potential of pig saliva to reflect stress responses via miRNAs [10]. Unlike serum, which requires invasive blood collection, and small intestine samples, which can only be obtained postmortem, saliva can be easily collected non-invasively and is considered an alternative sample to reflect real-time stress responses in piglets [10,18,19]. Owing to these advantages, saliva is increasingly recognized as a critical analytical sample for studying weaning stress in piglets.

This preliminary study aimed to explore weaning stress-associated differentially expressed miRNAs (DEMs) in the saliva and evaluate their potential as candidate indicators of physiological changes during the suckling-to-weaning transition in piglets. Additionally, we aimed to confirm the weaning stress-related trends in miRNA expression in the serum and small intestine, as previously reported, and compare them with those in saliva.

## Materials and methods

### Ethics statement

The Institutional Animal Care and Use Committee (approval number KNU_2025-04) of Kongju National University, Republic of Korea, approved all animal procedures.

### Animals and sample collection

Saliva samples were collected from 2-week-old suckling piglets (n = 4) and 5-week-old weaning piglets (n = 4) using Salivette tubes (Sarstedt, Nümbrecht, Germany). To minimize saliva sample contamination, feeding and watering were restricted for approximately 30 min before sample collection. The piglets were allowed to chew a cotton roll of the Salivette tubes for 2–3 min and the tubes were centrifuged at 3,000 rpm and 4 °C for 20 min.

### Library preparation and small RNA sequencing

Small RNA was isolated from saliva using the XENOPURETM Plasma/Serum Small RNA Purification Kit (Xenohelix, Incheon, Republic of Korea). A small RNA sequencing library was constructed using the TruSeq Small RNA Library Preparation Kit (Illumina, San Diego, CA, USA), following the manufacturer’s protocol. Sequencing was performed using a NovaSeq X sequencer (Illumina, San Diego, CA, USA) to generate 50 bp single-end reads.

### Differentially expressed miRNA analysis

The raw sequence read quality was controlled using FastQC (v0.12.1) [20]. Adaptor and low-quality sequences were trimmed from small RNA sequencing reads using Cutadapt (v4.7) [21]. Reads shorter than 15 nt were discarded after trimming. Trimmed reads were aligned to the *Sus scrofa* reference genome (*S. scrofa* 11.1.112; GCA_000003025.6) using Bowtie1 (v1.3.1) [22]. The unique reads were mapped to porcine miRNA sequences in miRBase 22.1 [23] using the mapper.pl code of miRDeep2 [24]. Raw miRNA counts were normalized using the varianceStabilizingTransformation function of the DESeq2 package (v1.46.0) [25] in R. Principal component analysis (PCA) of normalized miRNA count data was performed using the prcomp function in the R package. DEMs were analyzed using the R Bioconductor package DESeq2 (v1.46.0) [25] based on a negative binomial generalized linear model and Wald test; the resulting p-values were corrected using the Benjamini-Hochberg procedure [26]. The miRNAs were considered as differentially expressed at an absolute value of log2 fold change (FC) ≥ 1 and on an adjusted false discovery rate (FDR) corrected p-value < 0.05. DEM function and DEM–target gene networks were visualized using Cytoscape (v3.10.3) [27].

### Target gene prediction and functional annotation

Potential target genes of the DEMs were predicted using TargetScan (v8.0) [28]. In further analyses, target genes with a probability of conserved targeting (PCT) [29] more than 95 were used. The Database for Annotation, Visualization, and Integrated Discovery (DAVID) tool [30] was used for the functional annotation of Gene Ontology (GO) and Kyoto Encyclopedia of Genes and Genomes (KEGG) pathways of the DEMs (p < 0.05). The enriched GO terms were analyzed using the REVIGO tool [31] and represented in treemaps.

## Results and discussion

### Overview of small RNA sequencing data

Small RNA sequencing was performed on the saliva samples from suckling and weaning piglets, generating average 72 million 50 bp single-end reads per library. After adapter removal and quality filtering, clean reads were obtained (Table 1). The Q20 and Q30 percentages for all samples were ≥95.0. The clean reads were mapped to the Sus scrofa reference genome using the Bowtie1 tool [22], with the percentage of mapped reads of all samples ranging between 5.84% and 25.34%. The relatively low mapping rate observed was similar to that reported in humans [32], likely owing to the high bacterial abundance in the oral cavity [33].

**Table 1:** Summary statistics of small RNA-seq data.

Group	Samples	Raw Reads	Clean Reads	Q20%	Q30%	Mapped Reads (%)
Suckling	S1	71,787,311	36,447,719	99.0	96.6	11.88
	S2	72,188,611	49,309,420	99.0	96.9	5.84
	S3	71,974,745	42,925,580	98.1	95.3	10.56
	S4	72,430,020	51,750,817	98.5	95.8	11.16
Weaning	W1	72,557,847	44,143,639	99.2	97.2	9.45
	W2	71,709,256	25,984,820	98.8	96.0	18.15
	W3	72,573,271	41,877,493	99.0	96.8	8.05
	W4	72,320,825	25,833,747	98.7	96.1	25.34

Known and novel miRNAs were predicted based on the miRBase v22.1 database, which provides miRNA sequences and annotations. In total, 457 porcine miRNAs were annotated using miRBase, of which 341 were expressed in piglets (Fig 1; S1 Table). Additionally, 1,252 novel miRNAs (S2 Table), predicted based on the seed sequences of *Bos taurus*, *Equus caballus*, *Ovis aries*, *Canis familiaris*, and *Homo sapiens* miRNAs, were identified, which were higher than the number of annotated miRNAs in the database. This suggests that the number of annotated miRNAs in miRBase is considerably smaller than that in humans and other species [34], indicating the need for further investigation on novel miRNAs identified in this study.

**Fig 1:**
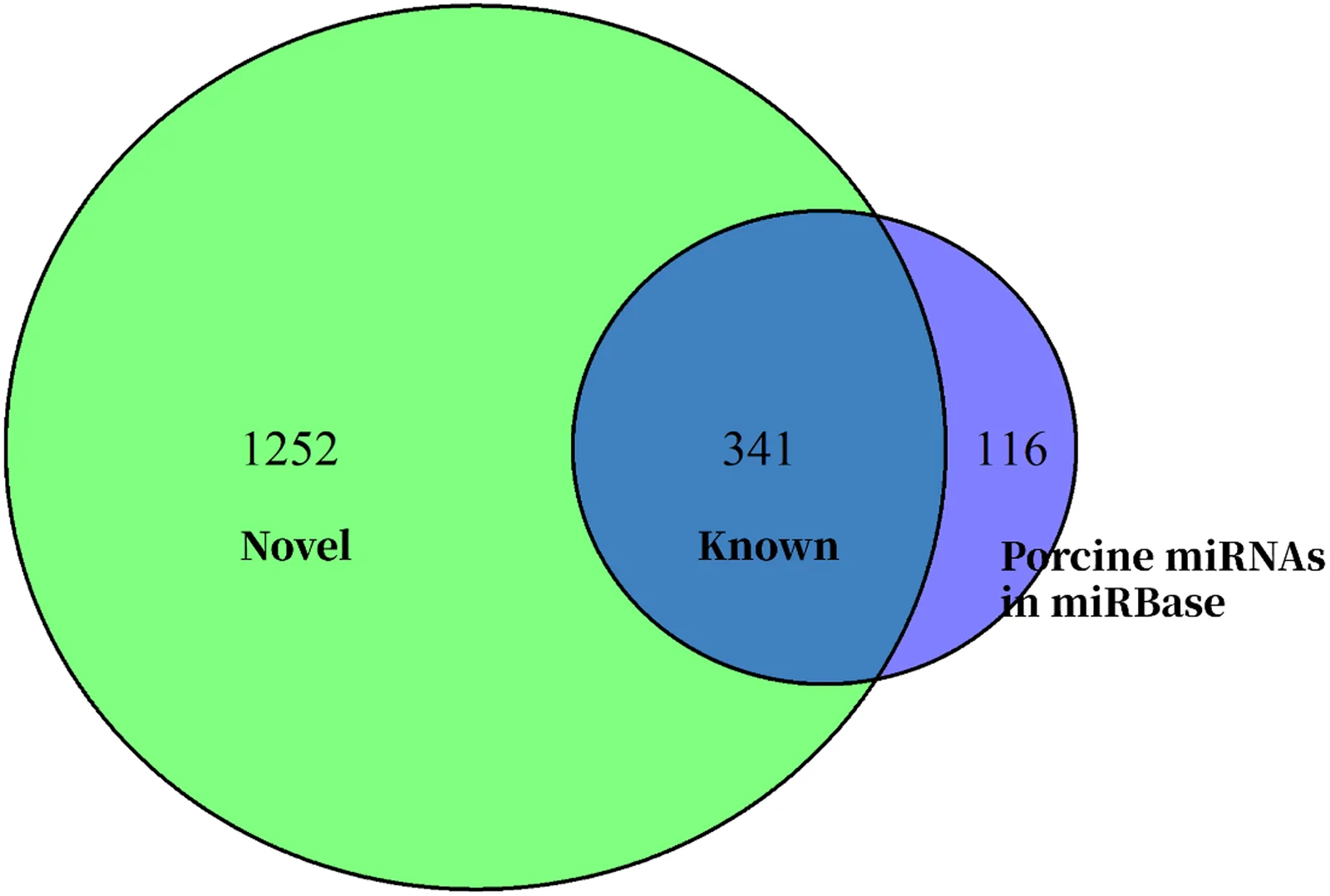
Venn diagram of known and novel microRNAs (miRNAs) detected across all samples. The green circle shows the number of novel and known miRNAs in the study. The novel miRNAs were predicted by comparing seed sequences with those of other species. The purple circle shows porcine miRNAs annotated in miRBase. The intersection represents known miRNAs annotated in miRBase that were expressed in the piglets. A total of 341 known and 1,252 novel miRNAs were identified in the samples.

Additionally, the top 10 most abundant miRNAs in weaning piglets were selected and compared with those in suckling piglets (Fig 2). The result revealed that the most abundant miRNAs in weaning piglets were: such as ssc-miR-27a, ssc-miR-27b-3p, ssc-miR-21-5p, ssc-miR-205, ssc-miR-16. Among these, ssc-miR-27a, ssc-miR-27b-3p, ssc-miR-21-5p, and ssc-miR-205 showed relatively high expression levels in weaning piglets, whereas ssc-miR-16 and ssc-miR-486 exhibited relatively low expression levels. These differences in expression levels were observed between weaning and suckling piglets, based on which, DEMs were identified.

**Fig 2:**
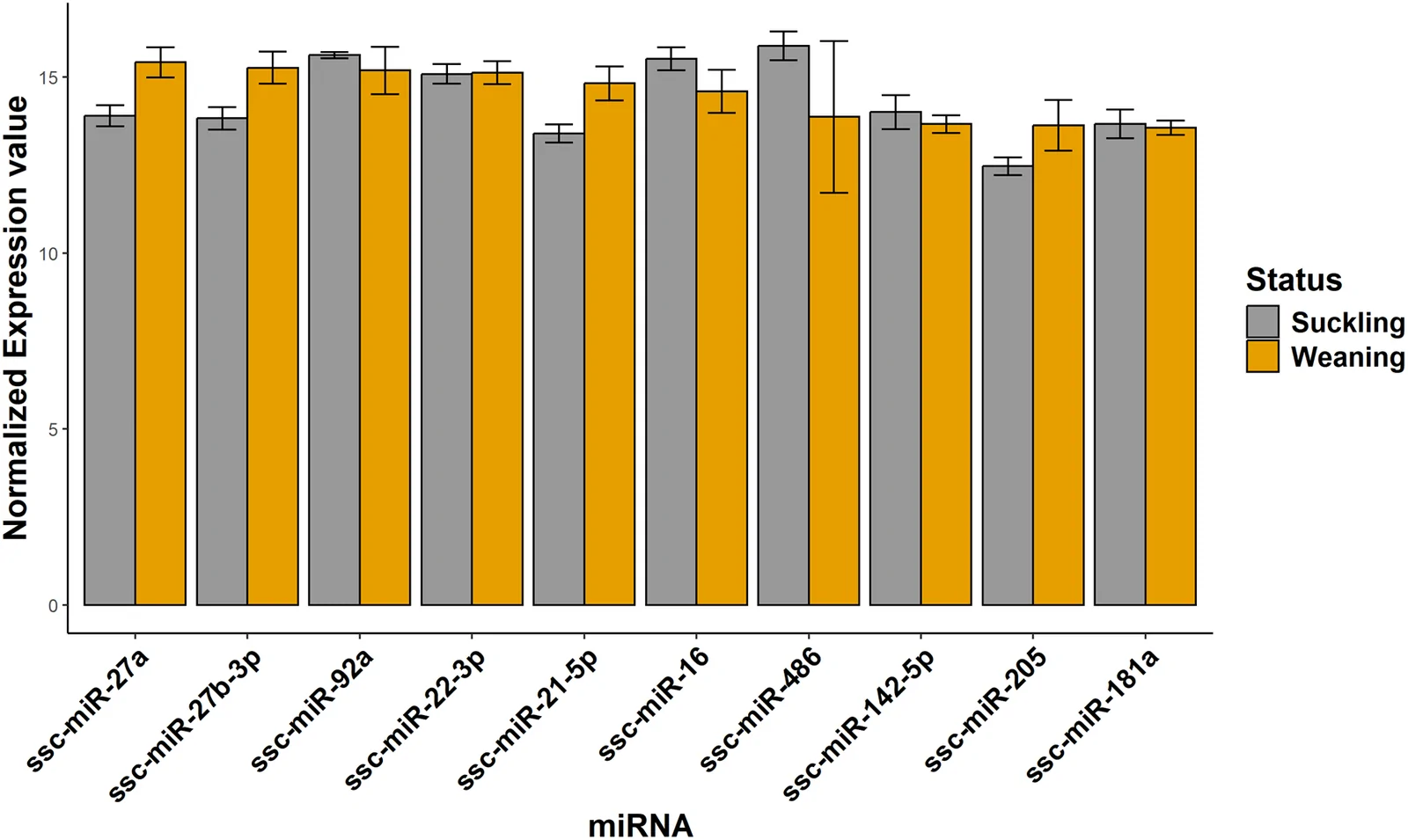
The expression levels of the most abundant microRNAs (miRNAs) in weaning piglets. The expression levels of the top 10 most abundant miRNAs in weaning piglets were compared with those in suckling piglets. The expression levels are normalized DESeq2’s median of ratio. Gray and orange bars represent the expression levels in suckling and weaning piglets, respectively.

### Global change in miRNA expression across suckling and weaning piglets by principal component analysis

Principal component analysis was performed to examine the clustering of miRNA expression in the saliva of suckling and weaning piglets. The PCA results showed that PC1 accounted for 47.6% variance, whereas PC2 accounted for 19.6% (Fig 3a). Furthermore, the miRNA profiles of suckling and weaning piglets clustered separately (Fig 3b). These findings suggest that salivary miRNA expression patterns vary between the two groups, reflecting their distinct physiological states. Tao et al [17] and Jang and Lee [16] have previously demonstrated similar differences in miRNA expression patterns in the small intestine and serum of suckling and weaning piglets. They suggested that the differences in miRNA expression between the two groups might be related to the stress responses associated with each physiological state. Weaning piglets experience weaning stress, which affects their hormonal changes and immune responses. These stressors induce alterations in miRNA expression patterns, highlighting the potential of salivary miRNAs as important indicators of physiological state. Therefore, the clustering observed in this study established that the differences in miRNA expression between suckling and weaning piglets are distinguishable, providing critical data for future analyses and applications.

**Fig 3:**
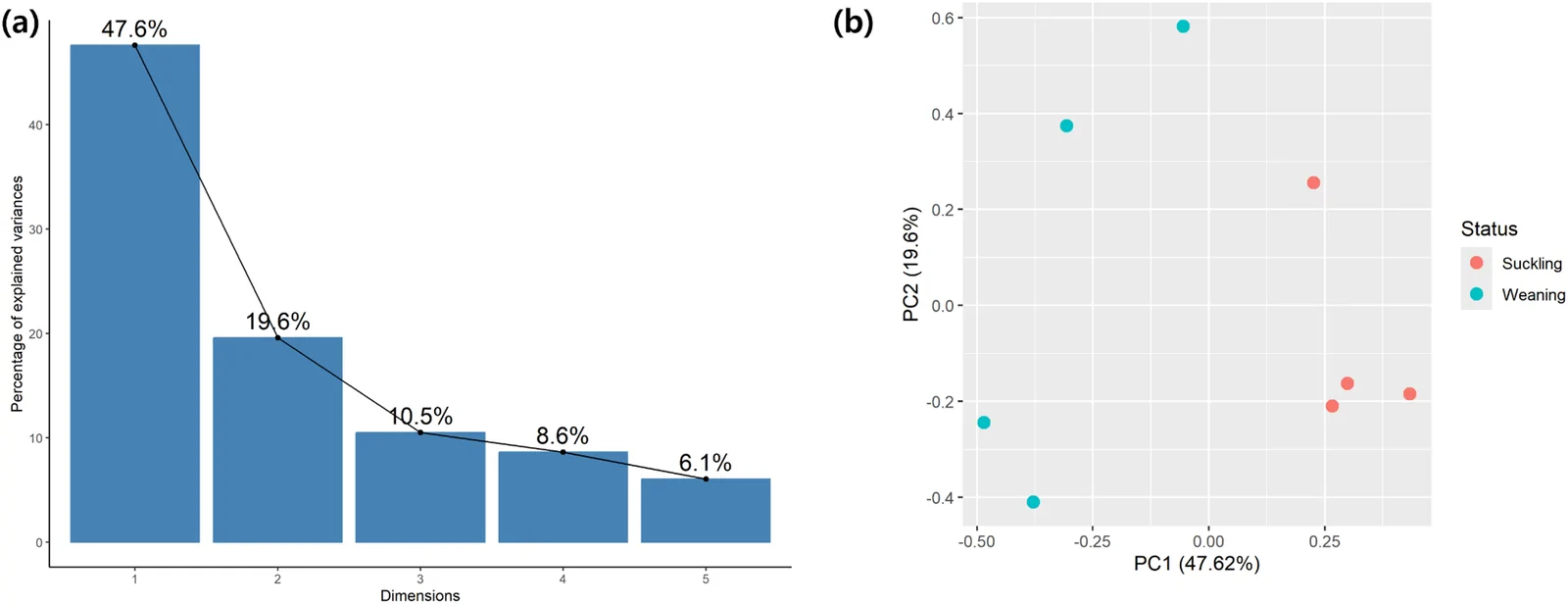
Principal component analysis (PCA) of salivary microRNA (miRNA) expression in suckling and weaning piglets. (a) Screen plot showing the percentage of variance explained by each principal component (PC). PC1 explains 47.6% variance, followed by PC2 (19.6%). (b) PCA scatter plot illustrating miRNA profile clustering between the suckling (red) and weaning (blue) groups.

### Identification of differentially expressed salivary miRNAs in suckling and weaning piglets

We identified DEMs in the saliva of suckling and weaning piglets (Fig 4a). Hierarchical clustering separated the samples into two distinct groups according to weaning status, indicating a marked difference in the miRNA expression profiles between the two groups (Fig 4b). Among the 341 miRNAs detected, a total of 41 DEMs were identified, of which 20 were highly expressed and 21 were lowly expressed in the weaning group (Tables 2 and 3). Notably, ssc-miR-138, ssc-miR-146b, and ssc-miR-155-5p exhibited log2 fold changes greater than 2, with statistically significant differences (FDR < 0.05; Table 2), suggesting their potential involvement in the physiological adaptation to weaning. These miRNAs have previously been reported to play roles in the stress response and immune regulation [36,37,52]. Lowly expressed miRNAs showed log2 fold changes ranging from −1.01 to −6.55, indicating a substantial decrease in expression in weaning piglets (Table 3). Particularly, ssc-miR-214-5p, ssc-miR-199a-5p, ssc-miR-9858-5p, and ssc-miR-127 showed the demonstrated the most prominent low expression levels. These miRNAs have also been implicated in diseases, stress, and immune responses [48–51] and their reduced expression may reflect regulatory changes in response to weaning-associated stress. These findings provide insights into miRNA-mediated regulation during weaning and highlight the role of miRNAs in stress and immune responses.

**Table 2:** Differentially expressed salivary miRNAs with higher expression in weaning piglets than in suckling piglets.

miRNA	Mature sequence (5′-3′)	log2FC	FDR	Function	Reference
ssc-miR-138	AGCUGGUGUUGUGAAUCAGGC	4.23	3.93E − 13	Stress, Immune	[35]
ssc-miR-146b	UGAGAACUGAAUUCCAUAGGC	3.44	8.16E − 07	Stress, Immune	[36]
ssc-miR-155-5p	UUAAUGCUAAUUGUGAUAGGGG	2.75	2.28E − 07	Immune	[37]
ssc-miR-9843-3p	UCUGUGAACUAGAAACCUCUGG	2.36	0.008962	–	–
ssc-miR-429	UAAUACUGUCUGGUAAUGCCGU	2.30	2.61E − 07	–	–
ssc-miR-378b-3p	ACUGGACUUGGAGUCAGAAGU	2.15	5.67E − 05	–	–
ssc-miR-141	UAACACUGUCUGGUAAAGAUG	1.73	2.48E − 05	Immune	[38]
ssc-miR-146a-5p	UGAGAACUGAAUUCCAUGGGUU	1.72	0.000309	Immune	[39]
ssc-miR-378	ACUGGACUUGGAGUCAGAAGGC	1.58	8.10E − 06	–	–
ssc-miR-21-3p	CAACAGCAGUCGAUGGGCUGU	1.56	2.54E − 06	Disease	[40]
ssc-miR-221-3p	AGCUACAUUGUCUGCUGGGUUU	1.47	0.000367	Disease	[41]
ssc-miR-27a	UUCACAGUGGCUAAGUUCCGC	1.46	3.33E − 05	Disease	[42]
ssc-miR-210	CUGUGCGUGUGACAGCGGCUGA	1.40	0.003659	Disease	[43]
ssc-miR-27b-3p	UUCACAGUGGCUAAGUUCUGC	1.37	0.000162	Immune	[44]
ssc-miR-21-5p	UAGCUUAUCAGACUGAUGUUGA	1.35	9.45E − 05	–	–
ssc-miR-99b	CACCCGUAGAACCGACCUUGCG	1.24	0.000473	–	–
ssc-miR-29c	UAGCACCAUUUGAAAUCGGUUA	1.20	0.000136	Immune	[45]
ssc-miR-222	AGCUACAUCUGGCUACUGGGUCUC	1.14	0.00067	Immune, Disease	[41,46]
ssc-miR-345-3p	CCCUGAACUAGGGGUCUGGAG	1.13	0.045834	Immune	[47]
ssc-miR-205	UCCUUCAUUCCACCGGAGUCUG	1.13	0.008089	Stress	[17]

miRNA, MircroRNA; log2FC, log2 Fold Change; FDR, False discovery rate.

**Table 3:** Differentially expressed salivary miRNAs with lower expression in weaning piglets than in suckling piglets.

miRNA	Mature sequence (5′-3′)	log2FC	FDR	Function	Reference
ssc-miR-214-5p	UGCCUGUCUACACUUGCUGUGC	−6.55	0.002922	Disease	[48]
ssc-miR-199a-5p	CCCAGUGUUCAGACUACCUGUUC	−3.96	2.28E − 07	Stress	[49]
ssc-miR-9858-5p	UUCCUGAGUCGGACUGGGCU	−3.91	0.000734	Disease	[50]
ssc-miR-127	UCGGAUCCGUCUGAGCUUGGCU	−3.56	1.25E − 07	Immune	[51]
ssc-miR-199a-3p	ACAGUAGUCUGCACAUUGGUUA	−3.10	2.25E − 06	Disease	[50]
ssc-miR-199b-3p	UACAGUAGUCUGCACAUUGGUU	−3.10	2.25E − 06	Disease	[50]
ssc-miR-144	UACAGUAUAGAUGAUGUAC	−2.55	0.000409	Stress, Disease	[16,52]
ssc-miR-139-5p	UCUACAGUGCACGUGUCUCCAG	−2.50	0.017408	Immune, Disease	[53,54]
ssc-miR-10b	UACCCUGUAGAACCGAAUUUGU	−2.46	0.0002	Immune, Disease	[55]
ssc-miR-381-3p	UAUACAAGGACAAGCUCUCUGU	−2.34	0.000104	Immune	[56]
ssc-miR-199b-5p	CCCAGUGUUUAGACUAUCUGUU	−2.00	0.003451	Disease	[57]
ssc-miR-451	AAACCGUUACCAUUACUGAGUU	−1.88	0.007453	Stress	[16]
ssc-miR-4334-3p	UCCCUGUCCUCCAGGAGCUC	−1.87	2.28E − 07	Disease	[58]
ssc-miR-339	UCCCUGUCCUCCAGGAGCUCA	−1.85	2.28E − 07	Immune	[59]
ssc-miR-339-5p	UCCCUGUCCUCCAGGAGCUCAC	−1.85	2.28E − 07	Immune	[60]
ssc-miR-126-3p	UCGUACCGUGAGUAAUAAUGCG	−1.77	0.006674	Disease	[61]
ssc-miR-126-5p	CAUUAUUACUUUUGGUACGCG	−1.37	0.028683	Disease, Immune	[62]
ssc-miR-31	AGGCAAGAUGCUGGCAUAGCUG	−1.34	5.67E − 05	–	–
ssc-miR-143-3p	UGAGAUGAAGCACUGUAGCUC	−1.18	0.027158	–	–
ssc-miR-1306-5p	CCACCUCCCCUGCAAACGUCCA	−1.14	0.02032	Disease	[63]
ssc-miR-30c-5p	UGUAAACAUCCUACACUCUCAGC	−1.01	0.007611	Disease, Immune	[64,65]

miRNA, MircroRNA; log2FC, log2 Fold Change; FDR, False discovery rate.

**Fig 4:**
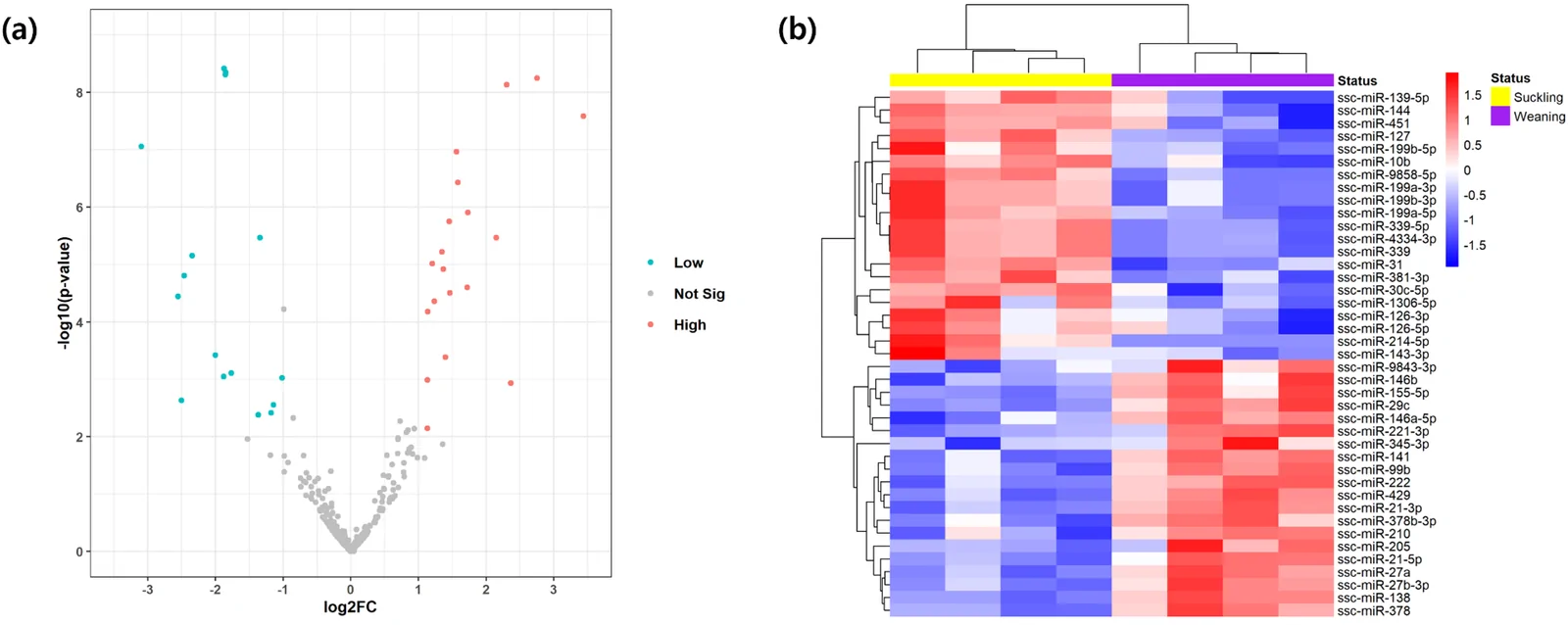
Differentially expressed microRNAs (DEMs) between suckling and weaning piglets. (a) Volcano plot of DEMs between weaning and suckling piglets. MicroRNAs (miRNAs) are classified into three categories: low (blue), not significant (gray), and high (red). (b) Heatmap showing DEM expression patterns across different samples. Red represents higher regulation, and blue represents lower regulation of miRNA expression in weaning piglets. The samples are grouped by status: weaning (W) and suckling (S), with the corresponding miRNA names listed on the y-axis.

### Functional annotation of the differentially expressed miRNA and their target genes

Fig 5 shows the functional networks related to immunity, stress responses, and disease diagnosis among the DEMs in suckling and weaning piglets. The highly expressed miRNAs in weaning piglets were mainly involved in immune modulation and inflammation control. Among these, ssc-miR-146b has been identified as a key miRNA related to weaning stress. It is highly expressed in the small intestine of weaning piglets, plays an essential role in immune and inflammatory regulation [15], modulates the inflammatory response of immune cells [66], and alleviates stress-induced intestinal tissue damage associated with stress [36]. Thus, increased miR-146b expression may contribute to inflammation suppression and tissue protection. miR-138, which showed the highest fold change, is known to respond to hypoxic stress in pulmonary artery smooth muscle cells [52], acting as a modulator of cell survival by inhibiting apoptosis and caspase activation. It is also involved in regulating inflammation and oxidative stress [67]. Weaning piglets are generally exposed to oxidative stress regardless of age [68,69], which negatively affects disease resistance and intestinal barrier function [2,6,70]. Therefore, increased ssc-miR-138 expression may play an important role in managing oxidative stress, promoting cell survival, and reducing inflammation. Overall, miRNAs with high expression were associated with essential functions, such as cell death regulation, inflammation, disease diagnostics, and viral infection responses, suggesting their role in modulating immune and inflammatory reactions during weaning stress.

**Fig 5:**
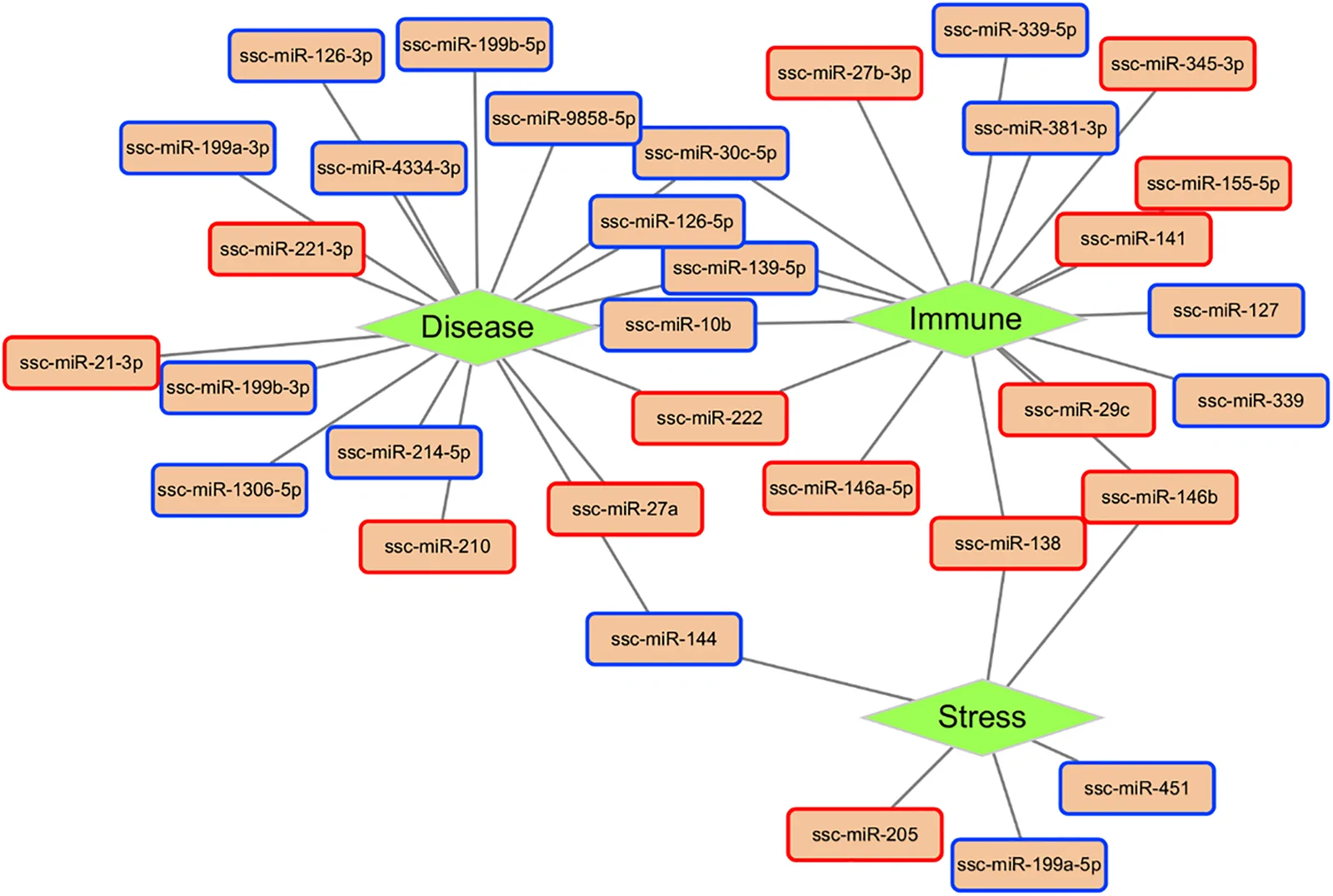
Differentially expressed microRNAs (DEMs) and their functional network. The network shows the relationships between DEMs categorized by their functional roles in Disease, Immune, and Stress. Green nodes represent functional categories, while the color of the node borders indicates regulation direction. Nodes with red and blue borders represent high- and low-regulated microRNAs in weaning piglets, respectively.

In contrast, miRNAs with low expression were predominantly involved in immune regulation, apoptosis, cell proliferation, and metastasis. Previous studies have also reported low expression of miR-30c-5p, miR-127, and miR-199a-5p in post-weaning serum [17] and miR-451 and miR-144 in both intestinal and serum samples [15,16]. miR-30c-5p, which tends to show low expression in oral squamous cell carcinoma, is known to inhibit apoptosis and autophagy via the eIF2α/ATF4 signaling pathway, thus promoting tumor cell survival [64,71]. Reduced gene expression in weaning piglets may contribute to sustained inflammation and cellular damage. miR-127 targets *B-cell lymphoma 6* (*Bcl6*), facilitating inflammatory macrophage development and inducing pulmonary damage [72], whereas miR-451 promotes gastric cancer cell proliferation and metastasis [73]. The low expression of miR-127 and miR-451 may suppress excess inflammatory responses and protect cells from damage, thereby contributing to immune homeostasis. miR-199a-5p, a critical miRNA involved in oxidative stress and apoptosis, may improve oxidative stress responses after weaning when its expression is reduced by upregulating *Brahma-related gene 1* (*Brg1*) [49]. In addition, miR-144 expression was low in the serum of patients with depression than in the serum of healthy participants [74], indicating a potential link to psychological stress in weaned piglets. Collectively, the lowly expressed miRNAs are involved in maintaining normal physiological responses by regulating inflammation and cell proliferation. These regulatory mechanisms contribute to the normalization of immune responses and cellular adaptation during weaning stress, and help maintain cellular homeostasis.

We predicted the target genes of DEMs in suckling and weaning piglets using TargetScan [28]. Since TargetScan v8.0 does not include a database for porcine miRNAs, we performed target gene prediction using human orthologs and identified 320 target genes for 14 DEMs. Network analysis, along with GO and KEGG pathway analyses, was conducted for DEMs and their predicted target genes. Network analysis revealed that *ZBTB20*, *PDE7B*, *NACC2*, and *BAZ2B* were the target genes regulated by more than three DEMs (Fig 6). Although these genes have been implicated in human diseases such as cancer [75–78], their functions in piglets remain unexplored, warranting further studies.

**Fig 6:**
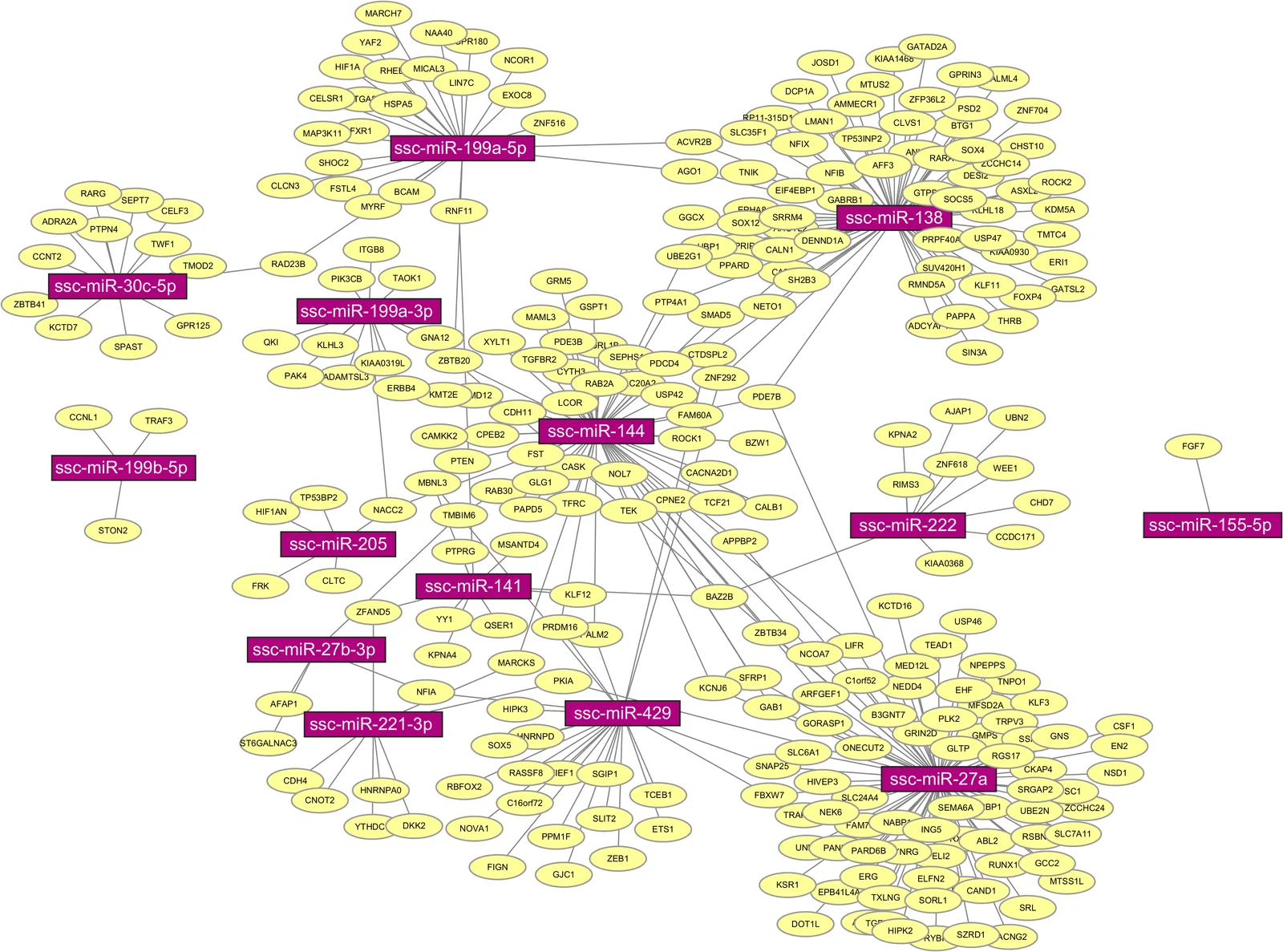
Differentially expressed microRNA (DEM)–target gene interaction network. The network illustrates interactions between DEMs and their predicted target genes under weaning stress in piglets. Yellow nodes represent the predicted target genes.

Fig 7 summarizes the GO analysis results with semantic similarity using REVIGO, visualized as a treemap. GO analysis revealed that positive regulation of DNA-templated transcription was the most prominent term in the Biological Process (BP) category, suggesting that miRNAs play a crucial role in regulating gene expression. Processes such as cell differentiation and chromatin remodeling are involved in regulating cell differentiation and gene expression, which are closely associated with weaning stress-induced physiological changes. Weaning piglets undergo rapid physiological changes during stress, indicating that miRNAs play an important role in modulating these processes. In the Cellular Component (CC) category, miRNAs were predominantly located in the nucleoplasm, with additional observations in the perinuclear region of the cytoplasm, neuron projections, and the receptor complex, highlighting their involvement in cellular signaling. Stress responses in weaning piglets affect various intracellular signaling pathways and miRNAs play pivotal roles in regulating these pathways within the nucleus and cytoplasm. Notably, miRNAs may regulate immune responses to stress by modulating interactions with cell membranes and intracellular signaling pathways, which is a significant finding. Protein serine/threonine kinase activity and transcription coactivator binding were identified as the main functions in the Molecular Function (MF) category, along with DNA-binding transcription factor activity and RNA polymerase II-specific functions. These findings demonstrate that during stress in weaning piglets, miRNAs regulate gene expression and control the activation of proteins and transcription processes necessary for the stress response. These functions have significant implications in regulating inflammatory responses, cellular physiological changes, and immune system modulation under stressful conditions.

**Fig 7:**
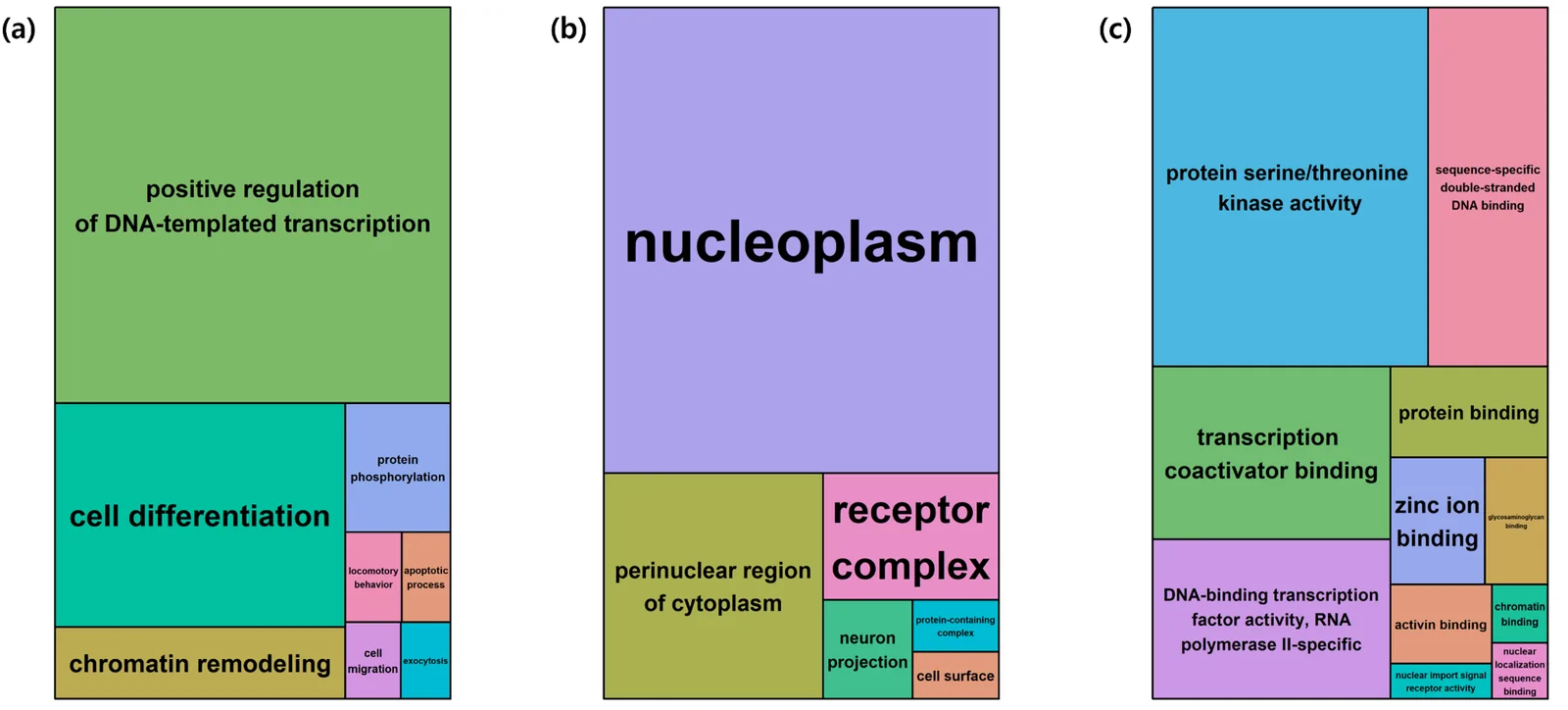
Gene Ontology (GO) treemaps of target genes for differentially expressed microRNAs (DEMs) between suckling and weaning piglets. GO terms representing (a) Biological Processes (BP), (b) Cellular Components (CC), and (c) Molecular Functions (MF) were analyzed and visualized using REVIGO. Each rectangle represents a specific GO term, with the size of the rectangle reflecting the significance (−log10 *p*-value) of the term, and colors representing related functional categories.

Further investigating the BP terms for the target genes of highly expressed miRNAs revealed high enrichment in processes such as promyelocytic leukemia (PML) body organization and negative regulation of granulocyte differentiation (Fig 8). Negative regulation of granulocyte differentiation refers to any process that inhibits, prevents, or reduces granulocyte differentiation frequency, rate, or extent. Under weaning stress, downregulating this process may enhance granulocyte production, which could facilitate rapid immune activation and contribute to effective responses against stress and potential infections.

**Fig 8:**
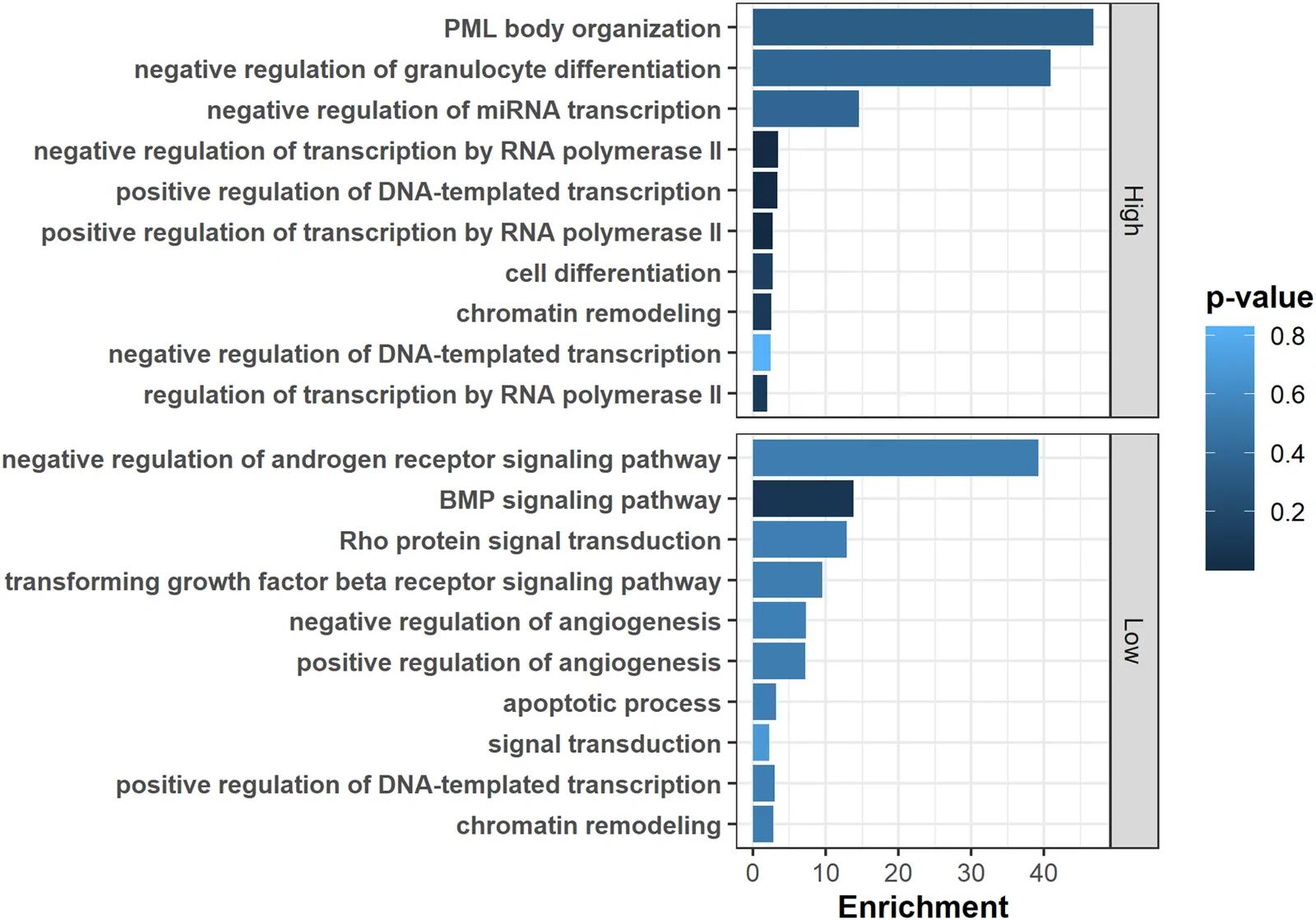
Enrichment analysis of Biological Process (BP) for target genes of differentially expressed microRNAs (DEMs) in response to weaning stress. The X-axis represents the enrichment score, and the Y-axis represents the different Gene Ontology (GO) BP terms. High and low enrichment scores are shown for the high- and low-regulated microRNAs, respectively.

KEGG pathway analysis revealed that the target genes of highly expressed miRNAs were enriched in pathways involved in suppressing excess cell growth and immune responses, including the Ras signaling, cellular senescence, and acute myeloid leukemia pathways (Fig 9). These findings suggested a regulatory mechanism aimed at controlling overactive responses and maintaining cellular functions during stress. In contrast, the target genes of lowly expressed miRNAs with low expression were enriched in pathways such as the TGF-beta signaling pathway, AMPK signaling pathway, and autophagy, which are essential for cellular signaling, energy homeostasis, and damaged cell clearance under stress conditions.

**Fig 9:**
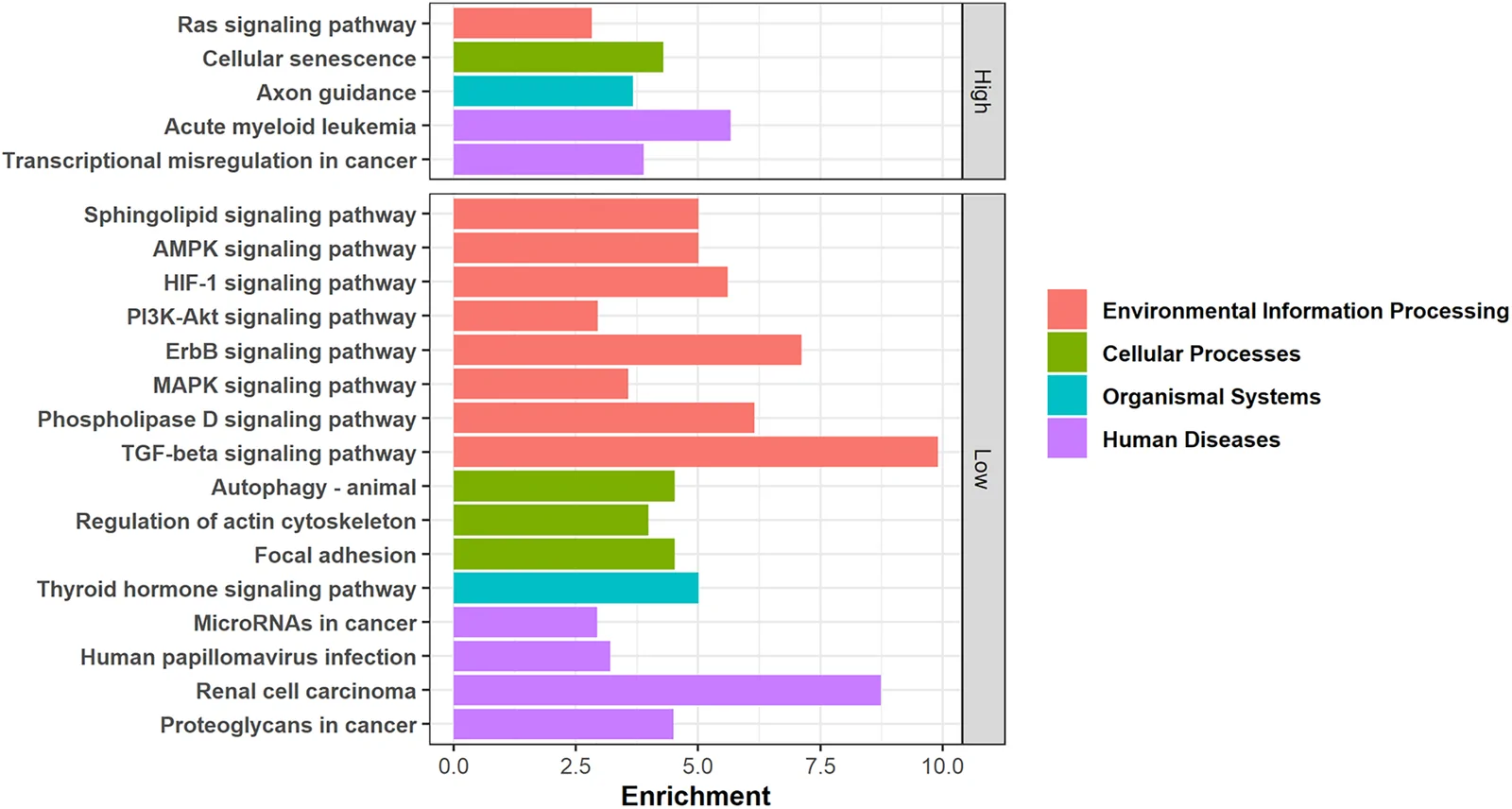
Kyoto Encyclopedia of Genes and Genomes (KEGG) pathway enrichment analysis for target genes of differentially expressed microRNAs (DEMs) in response to weaning stress. The X-axis represents the enrichment score, and the Y-axis represents the various KEGG pathways. The pathways are classified into four categories: Environmental Information Processing, Cellular Processes, Organismal Systems, and Human Diseases. High and low enrichment scores are shown for the high- and low-regulated microRNAs, respectively.

Collectively, these results highlight the molecular mechanisms by which salivary miRNAs modulate the physiological responses to weaning stress in piglets. These findings provide a valuable foundation for future studies of miRNA-based biomarkers related to stress and immune regulation.

### Comparison of DEMs in the saliva, serum, and small intestine under weaning stress

Among the 20 most highly expressed salivary miRNAs in weaning piglets, 10 (ssc-miR-138, ssc-miR-146b, ssc-miR-378, ssc-miR-221-3p, ssc-miR-210, ssc-miR-27b-3p, ssc-miR-99b, ssc-miR-29c, ssc-miR-222, and ssc-miR-205) showed expression patterns consistent with those reported in previous studies (Fig 10) [15–17]. Similarly, 8 out of 21 lowly expressed miRNAs showed matching trends. These miRNAs play crucial roles in immune responses, inflammation, stress, infection, and disease diagnosis, indicating that weaning stress contributes to physiological and immunological alterations in piglets. In addition, many salivary miRNAs exhibited expression patterns similar to those observed in serum and the small intestine, suggesting that saliva may serve as a promising alternative or complementary sample for conventional serum- or tissue-based analyses. Owing to its non-invasive collection and suitability for repeated sampling, saliva may be a useful matrix for identifying candidate biomarkers associated with stress and immune status in piglets.

**Fig 10:**
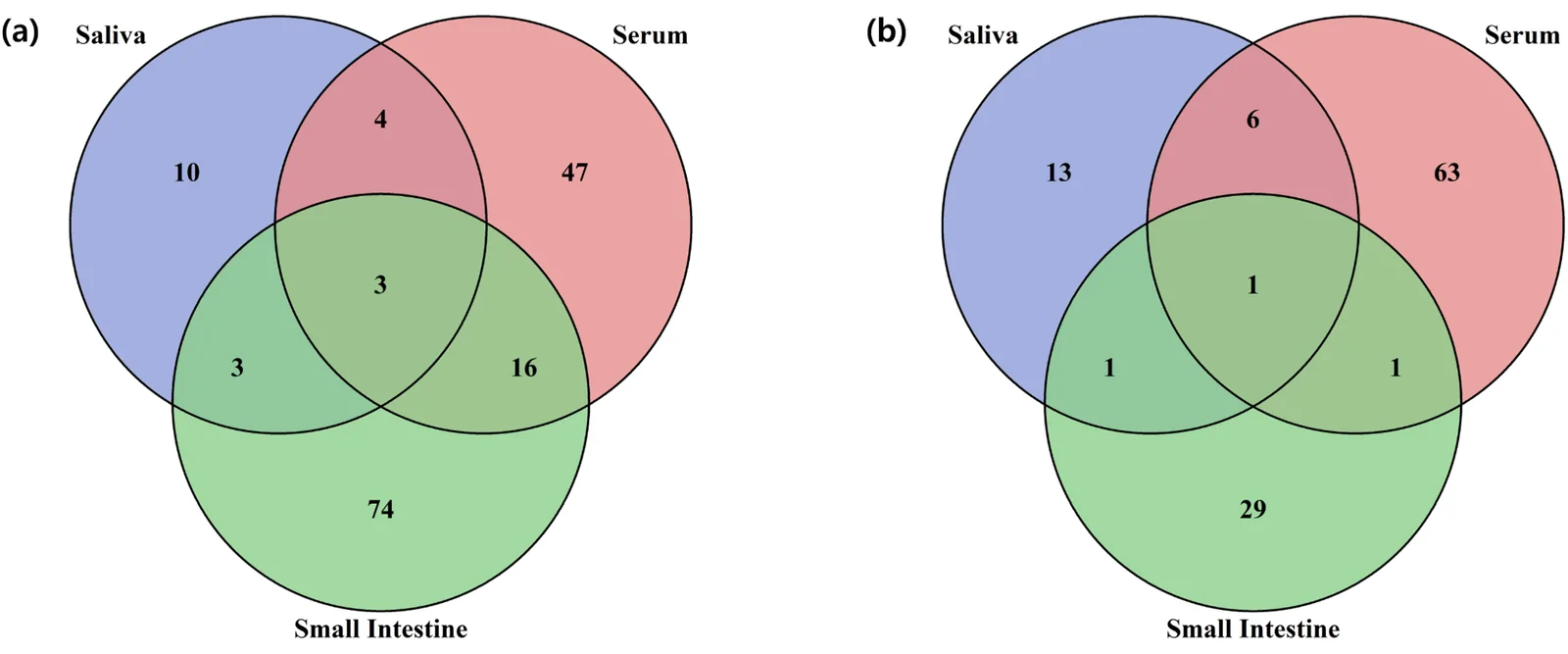
Venn diagram comparing differentially expressed microRNAs (DEMs) in the serum, small intestine, and saliva under weaning stress. (a) Comparison of high-expressed and (b) low-expressed microRNAs, with the numbers in the overlapping areas indicating the number of DEMs in all regions. The DEMs in the small intestine and serum were based on those reported in previous studies (Tao and Xu 2013; Tao et al. 2016; Jang and Lee 2021), with comparisons to saliva presented in this study.

## Conclusion

This preliminary study investigated the differential expression of salivary miRNAs in piglets in response to weaning stress, suggesting their potential as non-invasive biomarkers. A total of 41 DEMs were identified, several of which were associated with immune responses, inflammation regulation, and oxidative stress. In addition, several salivary miRNAs showed expression patterns similar to those previously reported in serum and small intestine, suggesting that saliva may serve as a non-invasive matrix for exploring molecular indicators of weaning-associated physiological changes. Functional annotation via GO and KEGG pathway analyses further demonstrated that these miRNAs are involved in stress-related biological processes and signaling pathways. These findings support the feasibility of salivary miRNA profiling as a real-time, non-invasive method for monitoring physiological stress in piglets. However, because of the limited number of animals and the transient nature of weaning stress, further validation using larger cohorts, longitudinal sampling designs, and cost-effective targeted assays are required before salivary miRNAs can be applied as practical biomarkers for piglet health management.

## Supporting information

S1 Table List of identified known miRNAs detected in the saliva of suckling and weaning pigs. (PDF)

S2 Table List of novel miRNAs predicted from the saliva of suckling and weaning pigs. (PDF)
