# Supplementary material for: Investigation of salivary microRNAs as non-invasive biomarkers for weaning stress in piglets: A preliminary study
Source: PLoS One. 2026 Oct 13;21(10):e0360727. doi: 10.1371/journal.pone.0360727 (PMC13660905; doi:10.1371/journal.pone.0360727)
Supplement: Supplementary file 1 [file pone.0360727.s001.pdf]

S1 Table. List of identified known miRNAs detected in the saliva of suckling and weaning pigs.

Known miRNA	CHR	Mature sequence (5'-3')	Known miRNA Names with the Same Seed
ssc-let-7a	5	UGAGGUAGUAGGUUGUAUAGU	bta-let-7f
ssc-let-7c	13	UGAGGUAGUAGGUUGUAUGGU	bta-let-7f
ssc-let-7d-3p	3	CUAUACGACCUGCUGCCUUUCU	-
ssc-let-7d-5p	3	AGAGGUAGUAGGUUGCAUAGUU	-
ssc-let-7e	6	UGAGGUAGGAGGUUGUAUAGU	bta-let-7f
ssc-let-7f-3p	3	CUAUACAAUCUAUUGCCUUCC	-
ssc-let-7f-5p	3	UGAGGUAGUAGAUUGUAUAGU	bta-let-7f
ssc-let-7g	13	UGAGGUAGUAGUUUGUACAGU	bta-let-7f
ssc-let-7i-3p	5	CUGCGCAAGCUACUGCCUUGCU	-
ssc-let-7i-5p	5	UGAGGUAGUAGUUUGUGCUGU	bta-let-7f
ssc-miR-100	9	AACCCGUAGAUCCGAACUUG	bta-miR-99a-5p
ssc-miR-101	6	UACAGUACUGUGAUAACUGAAG	bta-miR-101
ssc-miR-103	16	AGCAGCAUUGUACAGGGC	bta-miR-103
ssc-miR-10382	14	UCUGACCCUGGCCCCUGACCC	cfa-miR-8853
ssc-miR-10383	2	UGGUGCCUGACGUCUUGGCAG	-
ssc-miR-10384	2	CACAGGAAGGCCACGGAGGGGCC	hsa-miR-3193
ssc-miR-10386	6	GUCGUCCUCUCCCUCCCUCCU	-
ssc-miR-10387	7	CUGAUAGAGCUCCUGGAAGGCCU	-
ssc-miR-10388	X	CACAACUCUGGGAAGUACCAUU	-
ssc-miR-10390	9	AUACUACUGACAGACCGCAACCU	-
ssc-miR-10391	15	AAGGAAGGAGACUAACUCCGCC	-
ssc-miR-106a	X	AAAAGUGCUUACAGUGCAGGUAGC	bta-miR-20a
ssc-miR-107	14	AGCAGCAUUGUACAGGGC	bta-miR-103
ssc-miR-10a-3p	12	CAAAUUCGUAUCUAGGGGAAU	-
ssc-miR-10a-5p	12	UACCCUGUAGAUCCGAAUUUG	bta-miR-10a
ssc-miR-10b	15	UACCCUGUAGAACCGAAUUUG	bta-miR-10a

ssc-miR-1249	5	ACGCCCUUCCCCCCCUUCUUC	bta-miR-1249
ssc-miR-125a	6	UCCCUGAGACCCUUUAACCUG	bta-miR-125a
ssc-miR-125b	13	UCCCUGAGACCCUAACUUGUGA	bta-miR-125a
ssc-miR-126-3p	1	UCGUACCGUGAGUAAUAAUGCG	-
ssc-miR-126-5p	AEMK02000682.1	CAUUAUUACUUUUGGUACGCG	bta-miR-126-5p
ssc-miR-127	7	UCGGAUCCGUCUGAGCUUGGC	bta-miR-127
ssc-miR-1271-3p	2	UGCCUGCUAUGUGCCAGGCA	-
ssc-miR-1271-5p	2	CUUGGCACCUAGUAAGCACUC	bta-miR-96
ssc-miR-1277	X	UAUAUAUAUAUAUGUACGUA	hsa-miR-5011-5p
ssc-miR-128	15	UCACAGUGAACCGGUCUCUU	bta-miR-128
ssc-miR-1285	1	CUGGGCAACAUAGCGAGACCCCGU	-
ssc-miR-1296-5p	14	UUAGGGCCCUGGCUCCAUC	bta-miR-1296
ssc-miR-129a-3p	2	AAGCCCUUACCCCAAAAAGCAU	-
ssc-miR-129a-5p	2	CUUUUUGCGGUCUGGGCUUG	bta-miR-129
ssc-miR-129b	18	CUUUUUGCGGUCUGGGCU	bta-miR-129
ssc-miR-1306-3p	14	ACGUUGGCUCUGGUGGUGAUG	-
ssc-miR-1306-5p	14	CCACCUCCCCUGCAAACGUCCA	bta-miR-1306
ssc-miR-1307	14	UCGACCGGACCUCGACCGGC	hsa-miR-1307-5p
ssc-miR-130a	2	CAGUGCAAUGUUAAAAGGGCA	bta-miR-130a
ssc-miR-130b-3p	14	CAGUGCAAUGAUGAAAGGGCAU	-
ssc-miR-130b-5p	14	CAGUGCAAUGAUGAAAGGGCA	bta-miR-130a
ssc-miR-132	12	GUGGCUUUCGAUUGUUAC	bta-miR-132
ssc-miR-133a-3p	-	UUGGUCCCCUUCAACCAGCUG	-
ssc-miR-1343	2	CUCCUGGGGCCCGCACUCUCGC	bta-miR-1343-3p
ssc-miR-135	5	UAUGGCUUUUUAUUCCUAUGUGA	bta-miR-135a
ssc-miR-136-3p	7	CAUCAUCGUCUCAAAUGAGUCU	-
ssc-miR-136-5p	7	CAUCAUCGUCUCAAAUGAGUC	hsa-miR-136-3p
ssc-miR-137	4	UUAUUGCUUAAGAAUACGCGUAG	bta-miR-137
ssc-miR-138	13	AGCUGGUGUUGUGAAUCAGGCCG	bta-miR-138

ssc-miR-1388	-	AGGACUGUCAAACCUGAGAAUG	-
ssc-miR-139-5p	9	UCUACAGUGCACGUGUCUCCAG	bta-miR-139
ssc-miR-140-3p	-	UACCACAGGGUAGAACCACGGAC	-
ssc-miR-140-5p	6	ACCACAGGGUAGAACCACGGAC	cfa-miR-140
ssc-miR-141	5	UAACACUGUCUGGUAAAGA	bta-miR-200a
ssc-miR-142-3p	12	UGUAGUGUUUCCUACUUUAUGG	-
ssc-miR-142-5p	12	CAUAAAGUAGAAAGCACUAC	bta-miR-142-5p
ssc-miR-143-3p	2	UGAGAUGAAGCACUGUAGCUC	bta-miR-143
ssc-miR-143-5p	2	UGAGAUGAAGCACUGUAGCUC	bta-miR-143
ssc-miR-144	12	UACAGUAUAGAUGAUGUAC	bta-miR-144
ssc-miR-145-3p	2	GGAUUCCUGGAAAUACUGUUCU	-
ssc-miR-145-5p	2	GUCCAGUUUUCCCAGGAAUCCC	bta-miR-145
ssc-miR-1468	X	CUCCGUUUGCCUGUUUUGCUGA	bta-miR-1468
ssc-miR-146a-3p	16	CCUGUGAAGUUUAGUUCUUCAG	-
ssc-miR-146a-5p	16	UGAGAACUGAAUUCCAUGGGU	bta-miR-146b
ssc-miR-146b	14	UGAGAACUGAAUUCCAUAGGCUG	bta-miR-146b
ssc-miR-148a-3p	18	UCAGUGCACUACAGAACUUUGU	-
ssc-miR-148a-5p	18	UCAGUGCACUACAGAACUUUG	bta-miR-148a
ssc-miR-148b-3p	5	UCAGUGCAUCACAGAACUUUGU	-
ssc-miR-148b-5p	5	UCAGUGCAUCACAGAACUUUG	bta-miR-148a
ssc-miR-149	15	UCUGGCUCCGUGUCUUCACUCCC	bta-miR-149-5p
ssc-miR-150	6	UCUCCCAACCCUUGUACCAG	bta-miR-150
ssc-miR-151-3p	-	CUAGACUGAAGCUCCUUGAGGA	-
ssc-miR-151-5p	4	UCGAGGAGCUCACAGUCUAG	bta-miR-151-5p
ssc-miR-152	12	AGGUUCUGUGAUACACUCCGAC	hsa-miR-152-5p
ssc-miR-153	18	UGCAUAGUCACAAAAGUGAUCA	hsa-miR-153-5p
ssc-miR-155-3p	13	UCCUACAUGUUAGCAUUAACA	-
ssc-miR-155-5p	13	UUAAUGCUAAUUGUGAUAGGGG	bta-miR-155
ssc-miR-15a	11	UAGCAGCACAUAAUGGUUUG	bta-miR-16b

ssc-miR-15b	13	UAGCAGCACAUCAUGGUUUACA	bta-miR-16b
ssc-miR-16	13	UAGCAGCACGUAAAUAUUGGCG	bta-miR-16b
ssc-miR-17-3p	11	ACUGCAGUGAAGGCACUUGUAG	-
ssc-miR-17-5p	11	CAAAGUGCUUACAGUGCAGG	bta-miR-20a
ssc-miR-181a	1	AACAUUCAACGCUGUCGGUGAG	bta-miR-181a
ssc-miR-181b	10	AACAUUCAUUGCUGUCGGUGGG	bta-miR-181a
ssc-miR-181c	2	AACAUUCAACCUGUCGGUGAG	bta-miR-181a
ssc-miR-181d-5p	2	AACAUUCAUUGUUGUCGGUGGG	bta-miR-181a
ssc-miR-182	18	UUUGGCAAUGGUAGAACUCACAC	bta-miR-182
ssc-miR-183	18	UAUGGCACUGGUAGAAUUCAC	bta-miR-183
ssc-miR-1839-3p	7	AGACCUACUUUUCUACCAACA	-
ssc-miR-1839-5p	7	AAGGUAGAUAGAACAGGUC	bta-miR-1839
ssc-miR-184	7	UGGACGGAGAACUGAUAAGG	bta-miR-184
ssc-miR-1842	3	UGGCUCUGCGAGGUCGGCUC	bta-miR-760-3p
ssc-miR-185	14	UGGAGAGAAAGGCAGUUCCUGA	bta-miR-185
ssc-miR-186-3p	6	GCCCAAAGGUGAAUUUUUUGGG	-
ssc-miR-186-5p	6	CAAAGAAUUCUCCUUUUGGGC	bta-miR-186
ssc-miR-187	6	CGUGUCUUGUGUUGCAGCCGG	bta-miR-12042
ssc-miR-18a	11	UAAGGUGCAUCUAGUGCAGA	bta-miR-18b
ssc-miR-18b	X	UAAGGUGCAUCUAGUGCAGU	bta-miR-18b
ssc-miR-190a	1	UGAUAUGUUUGAUAUAUUAGG	bta-miR-190a
ssc-miR-190b	4	UGAUAUGUUUGAUAUUGGGU	bta-miR-190a
ssc-miR-191	13	CAACGGAAUCCCAAAAGCAGC	bta-miR-191
ssc-miR-192	2	CUGACCUAUGAAUUGACAGCC	-
ssc-miR-193a-3p	12	AACUGGCCUACAAAGUCCCAGU	-
ssc-miR-193a-5p	12	AACUGGCCUACAAAGUCCCAG	bta-miR-193a-3p
ssc-miR-194a-3p	2	CCAGUGGGGCUGCUGUUAUCU	-
ssc-miR-194a-5p	2	UGUAACAGCAACUCCAUG	bta-miR-194
ssc-miR-194b-5p	10	UGUAACAGCGACUCCAUGUGGA	-

ssc-miR-195	12	UAGCAGCACAGAAAUAUUGGCA	bta-miR-16b
ssc-miR-196a	12	UAGGUAGUUUCAUGUUGUUGGG	bta-miR-196a
ssc-miR-196b-3p	18	CGACAGCACGACACUGCCUUCA	-
ssc-miR-196b-5p	18	CGACAGCACGACACUGCCUUCA	bta-miR-196a
ssc-miR-199a-3p	-	ACAGUAGUCUGCACAUUGGUUA	-
ssc-miR-199a-5p	2	ACAGUAGUCUGCACAUUGGU	bta-miR-199a-3p
ssc-miR-199b-3p	1	UACAGUAGUCUGCACAUUGGUU	-
ssc-miR-199b-5p	1	ACAGUAGUCUGCACAUUGGU	bta-miR-199a-3p
ssc-miR-19a	11	UGUGCAAAUCUAUGCAAAAC	bta-miR-19a
ssc-miR-19b	11	UGUGCAAAUCCAUGCAAAAC	bta-miR-19a
ssc-miR-200b	6	UAAUACUGCCUGGUAAUGAUGAC	bta-miR-200c
ssc-miR-204	1	UUCCCUUUGUCAUCCUAUGCC	bta-miR-204
ssc-miR-205	9	UCCUUCAUUCCACCGGAGUC	bta-miR-205
ssc-miR-208b	7	AUAAGACGAACAAAAGGUUUG	bta-miR-208a
ssc-miR-20a-3p	11	ACUGCAUUAUGAGCACUUAAAGU	-
ssc-miR-20a-5p	11	UAAAGUGCUUAUAGUGCAGGUAG	bta-miR-20a
ssc-miR-20b	X	ACUGUAGUGUGGGCACUUCCA	hsa-miR-20b-3p
ssc-miR-21-3p	12	CAACAGCAGUCGAUGGGCUGU	-
ssc-miR-21-5p	12	UAGCUUAUCAGACUGAUGUUGA	bta-miR-21-5p
ssc-miR-210	2	CUGUGCGUGUGACAGCGGCUGA	hsa-miR-210-3p
ssc-miR-212	12	UAACAGUCUCCAGUCACGGCCA	bta-miR-132
ssc-miR-214-3p	9	ACAGCAGGCACAGACAGGCAG	-
ssc-miR-214-5p	9	ACAGCAGGCACAGACAGGCAG	bta-miR-214
ssc-miR-215	10	AUGACCUAUGAAUUGACAG	bta-miR-215
ssc-miR-216	3	UAAUCUCAGCUGGCAACUGUGA	bta-miR-216a
ssc-miR-217	3	UACUGCAUCAGGAACUGAUUGGAU	-
ssc-miR-218	8	UUGUGCUUGAUCUAACCAUG	bta-miR-218
ssc-miR-218-5p	16	UUGUGCUUGAUCUAACCAUG	bta-miR-218
ssc-miR-218b	16	UUGUGCUUGAUCUAACCAUGUG	-

ssc-miR-219a	7	AGAGUUGAGUCUGGACGUCCCG	bta-miR-219
ssc-miR-22-3p	-	AAGCUGCCAGUUGAAGAACUGU	-
ssc-miR-22-5p	12	AAGCUGCCAGUUGAAGAACUG	bta-miR-22-3p
ssc-miR-221-3p	X	AGCUACAUUGUCUGCUGGGUU	bta-miR-221
ssc-miR-221-5p	X	AGCUACAUUGUCUGCUGGGUU	bta-miR-221
ssc-miR-222	X	AGCUACAUCUGGCUACUGGGUC	bta-miR-221
ssc-miR-223	X	UGUCAGUUUGUCAAAUACCCC	-
ssc-miR-224	X	CAAGUCACUAGUGGUUCCGUU	bta-miR-224
ssc-miR-2320-3p	6	CGAUGAUGGUCCCUGUGUUUG	-
ssc-miR-2320-5p	6	UGGCACAGGGUCCAGCUGUCGG	bta-miR-2320-5p
ssc-miR-23a	2	AUCACAUUGCCAGGGAUUUCC	bta-miR-23a
ssc-miR-23b	10	AUCACAUUGCCAGGGAUU	bta-miR-23a
ssc-miR-24-1-5p	2	UGGCUCAGUUCAGCAGGAAC	bta-miR-24-3p
ssc-miR-24-2-5p	10	UGGCUCAGUUCAGCAGGAAC	bta-miR-24-3p
ssc-miR-24-3p	-	UGGCUCAGUUCAGCAGGAACAG	-
ssc-miR-2411	4	GUGGAGUGACUGUCAGAUGCAG	-
ssc-miR-2483	X	AAACAUCUGGUUGGUUGAGAG	bta-miR-2483-3p
ssc-miR-26a	5	UUCAAGUAAUCCAGGAUAGGC	bta-miR-26a
ssc-miR-26b-3p	-	CCUGUUCUCCAUUACUUGGCU	-
ssc-miR-26b-5p	15	UUCAAGUAAUUCAGGAUAGGU	bta-miR-26a
ssc-miR-27a	2	UUCACAGUGGCUAAGUUC	bta-miR-27a-3p
ssc-miR-27b-3p	10	UUCACAGUGGCUAAGUUC	bta-miR-27a-3p
ssc-miR-27b-5p	10	UUCACAGUGGCUAAGUUC	bta-miR-27a-3p
ssc-miR-28-3p	13	CACUAGAUUGUGAGCUCCUGGA	-
ssc-miR-28-5p	13	CACUAGAUUGUGAGCUCC	eca-miR-28-3p
ssc-miR-296-3p	17	AGGGUUGGGCGGAGGCUU	-
ssc-miR-296-5p	17	AGGGUUGGGCGGAGGCUU	-
ssc-miR-299	AEMK02000452.1	UAUGUGGGACGGUAAACCGC	eca-miR-299
ssc-miR-29a-3p	18	CUAGCACCAUCUGAAAUCGGUUA	-

ssc-miR-29a-5p	18	UAGCACCAUCUGAAAUCGGU	bta-miR-29b
ssc-miR-29b	9	UAGCACCAUUUGAAAUCAGUGU	bta-miR-29b
ssc-miR-29c	9	UAGCACCAUUUGAAAUCGGU	bta-miR-29b
ssc-miR-301	-	CAGUCCAAUAGUAUUGUCAAAGC	-
ssc-miR-30a-3p	1	CUUUCAGUCGGAUGUUUGCAGC	-
ssc-miR-30a-5p	1	UGUAAACAUCCUCGACUGGAAGC	bta-miR-30d
ssc-miR-30b-3p	4	CUGGGAGGUGGAUGUUUACUU	-
ssc-miR-30b-5p	4	UGUAAACAUCCUACACUCAGC	bta-miR-30d
ssc-miR-30c-1-3p	6	CUGGGAGAGGGUUGUUUACU	-
ssc-miR-30c-3p	1	CUGGGAGAAGGCUGUUUACUCU	-
ssc-miR-30c-5p	1	UGUAAACAUCCUACACUCUCAGC	bta-miR-30d
ssc-miR-30d	4	UGUAAACAUCCCCGACUGGAAGC	bta-miR-30d
ssc-miR-30e-3p	6	CUUUCAGUCGGAUGUUUACAGC	-
ssc-miR-30e-5p	6	UGUAAACAUCCUUGACUGGAAGC	bta-miR-30d
ssc-miR-31	1	AGGCAAGAUGCUGGCAUAGC	bta-miR-31
ssc-miR-32	1	UAUUGCACAUUACUAAGU	bta-miR-92a
ssc-miR-320	14	AAAAGCUGGGUUGAGAGGGCGA	bta-miR-320a
ssc-miR-323	AEMK02000452.1	GCACAUUACACGGUCGACCUC	bta-miR-323
ssc-miR-324	12	CGCAUCCCCUAGGGCAUUGGUG	bta-miR-324
ssc-miR-326	9	CCUCUGGGCCCUUCCUCCAG	bta-miR-326
ssc-miR-328	6	CUGGCCCUCUCUGCCCUUCCG	bta-miR-328
ssc-miR-330	6	UCUCUGGGCCUGUGUCUUAGGC	bta-miR-326
ssc-miR-331-3p	5	GCCCCUGGGCCUAUCCUAGAA	-
ssc-miR-331-5p	5	GCCCCUGGGCCUAUCCUAGA	bta-miR-331-3p
ssc-miR-335	18	UUUUUCAUUAUUGCUCCUGAC	hsa-miR-335-3p
ssc-miR-338	12	UCCAGCAUCAGUGAUUUUGUUGA	bta-miR-338
ssc-miR-339	1	UCCCUGUCCUCCAGGAGCUCA	-
ssc-miR-339-3p	3	AGCUCCUCGAGGCCAGAGCCC	-
ssc-miR-339-5p	3	UCCCUGUCCUCCAGGAGCUCAC	-

ssc-miR-33b-3p	-	CAGUGCCUCGGCAGUGCAGCCC	-
ssc-miR-33b-5p	12	GUGCAUUGCUGUUGCAUUGCA	bta-miR-33a
ssc-miR-340	2	UUAUAAAGCAAUGAGACUGAU	eca-miR-340-5p
ssc-miR-342	7	UCUCACACAGAAAUCGCACCCG	bta-miR-342
ssc-miR-345-3p	-	CCCUGAACUAGGGGUCUGGAG	-
ssc-miR-345-5p	7	GCUGACUCCUAGUCCAGUGC	bta-miR-345-5p
ssc-miR-34a	6	UGGCAGUGUCUUAGCUGGUUG	bta-miR-34b
ssc-miR-34c	9	AGGCAGUGUAGUUAGCUGAUUGC	bta-miR-34b
ssc-miR-361-3p	X	CCCCCAGGUGUGAUUCUGAUUUGC	-
ssc-miR-361-5p	X	UUAUCAGAAUCUCCAGGGGUAC	bta-miR-361
ssc-miR-3613	11	ACAAAAAAAAAAGCCCAACCC	eca-miR-3613
ssc-miR-362	X	AACACACCUAUUCAAGGAUUCA	bta-miR-329a
ssc-miR-363	X	AAUUGCACGGUAUCCAUCUG	bta-miR-92a
ssc-miR-365-3p	-	UAAUGCCCCUAAAAAUCCUUAU	-
ssc-miR-365-5p	12	UAAUGCCCCUAAAAAUCCUUA	bta-miR-365-3p
ssc-miR-369	AEMK02000452.1	AAUAAUACAUGGUUGAUCU	bta-miR-369-3p
ssc-miR-370	7	GCCUGCUGGGGUGGAACC	bta-miR-370
ssc-miR-371-5p	6	ACUCAAACUGUGGGGGCACUU	eca-miR-371-5p
ssc-miR-374a-3p	X	CUUAUCAGGUUGUAUUGUAAUU	-
ssc-miR-374a-5p	X	UUAUAAUACAACCUGAUAAG	bta-miR-374a
ssc-miR-374b-3p	X	CUUAUCAGGUUGUAUUAUCAUU	-
ssc-miR-374b-5p	X	AUAUAAUACAACCUGCUAAG	bta-miR-374a
ssc-miR-375	15	UUUGUUCGUUCGGCUCGCGUGA	cfa-miR-375
ssc-miR-376a-3p	-	AUCAUAGAGGAAAAUCCACGU	-
ssc-miR-376a-5p	AEMK02000452.1	AUCAUAGAGGAAAAUCCA	bta-miR-376b
ssc-miR-378	2	ACUGGACUUGGAGUCAGAAGGC	bta-miR-378
ssc-miR-378b-3p	1	ACUGGACUUGGAGUCAGAAGU	-
ssc-miR-381-3p	-	UAUACAAGGACAAGCUCUCUGU	-
ssc-miR-382	AEMK02000452.1	GAAGUUGUUCGUGGUGGAUUCG	bta-miR-382

ssc-miR-411	AEMK02000452.1	UAGUAGACCGUAUAGCGUACG	eca-miR-411
ssc-miR-421-3p	X	AUCAACAGACAUUAAUUGGGCGC	-
ssc-miR-421-5p	X	AUCAACAGACAUUAAUUGGGCG	bta-miR-421
ssc-miR-423-3p	12	AGCUCGGUCUGAGGCCCCUCAGU	-
ssc-miR-423-5p	12	UGAGGGGCAGAGAGCGAGACUU	bta-miR-423-5p
ssc-miR-424-3p	-	CAAAACGUGAGGCGCUGCUAU	-
ssc-miR-424-5p	X	CAGCAGCAAUUCAUGUUUUGA	bta-miR-16b
ssc-miR-425-3p	13	AUCGGGAAUGUCGUGUCCGCCC	-
ssc-miR-425-5p	13	AAUGACACGAUCACUCCCGU	cfa-miR-425
ssc-miR-429	6	UAAUACUGUCUGGUAAUGCCG	bta-miR-200c
ssc-miR-432-5p	7	UCUUGGAGUAGGUCAUUGGGUG	bta-miR-432
ssc-miR-4331-3p	6	UGUGGCUGUGGUGUAGGCCAGC	-
ssc-miR-4331-5p	-	UGGCGUAGAUCACAGACACAGC	-
ssc-miR-4332	-	CACGGCCGCCGCCGGGCGCC	-
ssc-miR-4334-3p	3	UCCCUGUCCUCCAGGAGCUC	bta-miR-339a
ssc-miR-4338	6	UUCCCAUUGAGACUGAGCAUC	-
ssc-miR-450a	X	UUUUGCGAUGUGUUCCUAAUA	bta-miR-450a
ssc-miR-450b-5p	X	UUUUGCAAUAUGUUCCUGAA	bta-miR-450b
ssc-miR-450c-5p	X	UUUUGCGAUGUGUUCCUAAUAC	bta-miR-450a
ssc-miR-451	12	AAACCGUUACCAUUACUGAGUU	-
ssc-miR-452	X	UGUUUGCAGAGGAAACUGAGAC	bta-miR-452
ssc-miR-454	12	UAGUGCAAUAUUGCUUAUAGGG	bta-miR-130a
ssc-miR-455-3p	1	GCAGUCCAUGGGCAUAUACAC	-
ssc-miR-455-5p	1	UAUGUGCCUUUGGACUACAUCG	bta-miR-455-5p
ssc-miR-486	17	UCCUGUACUGAGCUGCCCCGAG	bta-miR-486
ssc-miR-490	-	CAACCUGGAGGACUCCAUGCUG	-
ssc-miR-490-3p	18	CAACCUGGAGGACUCCAUGCUG	-
ssc-miR-490-5p	18	CAACCUGGAGGACUCCAUGC	bta-miR-490
ssc-miR-491	1	AGUGGGGAACCCUUCCAUGAGG	bta-miR-491

ssc-miR-493-5p	7	UUGUACAUGGUAGGCUUUCAUUC	oar-miR-493-5p
ssc-miR-497	12	CAGCAGCACACUGUGGUUUG	bta-miR-16b
ssc-miR-499-3p	17	AACAUCACAGCAAGUCUGUGCU	-
ssc-miR-499-5p	17	UUAAGACUUGCAGUGAUGUU	bta-miR-499
ssc-miR-500-3p	X	AUGCACCUGGGCAAGGAUUCU	-
ssc-miR-500-5p	X	AUGCACCUGGGCAAGGAUUCUGA	cfa-miR-500
ssc-miR-503	X	UAGCAGCGGGAACAGUAC	bta-miR-6519
ssc-miR-504	X	AGACCCUGGUCUGCACUCUA	bta-miR-504
ssc-miR-505	X	CGUCAACACUUGCUGGUUUCC	bta-miR-505
ssc-miR-532-3p	X	CCUCCCACACCCAAGGCUUGCA	-
ssc-miR-532-5p	X	CAUGCCUUGAGUGUAGGACCG	bta-miR-532
ssc-miR-542-3p	-	UGUGACAGAUUGAUAACUGAAA	-
ssc-miR-542-5p	X	UGUGACAGAUUGAUAACUGA	eca-miR-542-3p
ssc-miR-545-3p	X	AUCAACAAACAUUUAUUGUGUG	-
ssc-miR-545-5p	X	UCAGUAAAUGUUUAUUGGAUGA	bta-miR-545-5p
ssc-miR-551a	6	GCGACCCACUCUUGGUUUCC	bta-miR-551a
ssc-miR-574-3p	8	CACGCUCAUGCACACACCCACA	-
ssc-miR-574-5p	8	CACGCUCAUGCACACACCCACA	cfa-miR-574
ssc-miR-582-3p	16	UAACCGGUUGAACAACUGAACC	-
ssc-miR-582-5p	16	AACCGGUUGAACAACUGAACC	eca-miR-582-3p
ssc-miR-615	5	UCCGAGCCUGGGUCUCCCUCU	-
ssc-miR-628-3p	1	UCUAGUAAGAGUGGCAGUCGA	hsa-miR-628-3p
ssc-miR-628-5p	1	UCUAGUAAGAGUGGCAGUCGA	hsa-miR-628-3p
ssc-miR-6516	12	AUCAUGUAUGAUACUGCAAACA	hsa-miR-6516-3p
ssc-miR-652	X	AAUGGCGCCACUAGGGUUG	bta-miR-652
ssc-miR-6529	13	GAGAGAUCAGAGGCGCAGAGU	-
ssc-miR-660	X	UACCCAUUGCAUAUCGGAGU	bta-miR-660
ssc-miR-664-3p	10	UAUUCAUUUAUCUCCCAGCCUACA	-
ssc-miR-664-5p	10	UUCAUUUAUCUCCCAGCCUACA	hsa-miR-5696

ssc-miR-671-3p	18	UCCGGUUCUCAGGGCUCCACC	-
ssc-miR-671-5p	18	UCCGGUUCUCAGGGCUCCACC	eca-miR-671-3p
ssc-miR-676-3p	-	CCGUCCUAAGGUUGUUGAGUU	-
ssc-miR-6782-3p	8	UGACCUCUGGUCUCCCGCCC	hsa-miR-6821-3p
ssc-miR-7-3p	10	CAACAAAUCACAGUCUGCCAUA	-
ssc-miR-7-5p	10	UGGAAGACUAGUGAUUUUGUUGU	bta-miR-7
ssc-miR-708-3p	9	CAACUAGACUGUGAGCUUCUAGA	-
ssc-miR-708-5p	9	CAACUAGACUGUGAGCUUCUAGA	eca-miR-9139
ssc-miR-7134-3p	5	AUGCGGAACCUGCGGAUACGG	-
ssc-miR-7134-5p	5	AUGCGGAACCUGCGGAUACGG	hsa-miR-6850-5p
ssc-miR-7135-3p	3	AUCUGUCUGUGUCUCUGAGCAG	-
ssc-miR-7135-5p	3	AUCUGUCUGUGUCUCUGAGCAG	hsa-miR-6888-3p
ssc-miR-7136-5p	7	UCUGGUCCAGACACUGUGGAGC	bta-miR-12063
ssc-miR-7137-3p	3	AGCUGGUCUGGGAGUUCCCGGG	-
ssc-miR-7137-5p	3	GGGGGAACUCCCAGACCAGC	hsa-miR-4471
ssc-miR-7139-3p	5	UAGGGCACAGGAUGGGAUGAGG	-
ssc-miR-7139-5p	5	CCAUUCCUUCGUCUGUGCAC	-
ssc-miR-7140-3p	17	AAUGAUGCCCCUUAGAGUUGAG	hsa-miR-1322
ssc-miR-7140-5p	17	AAUGAUGCCCCUUAGAGUUGAG	hsa-miR-1322
ssc-miR-9784-5p	12	AACGGGUUGUCUCAGUUGUCAGC	-
ssc-miR-7142-5p	2	UUUGUUGGCUCCUCUGAAGUGA	-
ssc-miR-7143-3p	9	UCUGCACUUGAAGCUGAGAC	eca-miR-7143
ssc-miR-7144-5p	11	ACUUUCCCGGGAUUUGGAGCGC	-
ssc-miR-885-3p	-	AGGCAGCGGGGUGUAGUGGAU	-
ssc-miR-758	AEMK02000452.1	UUUGUGACCUGGUCCACUAACC	bta-miR-758
ssc-miR-99a-3p	13	CAAGCUCGCUUCUAUGGGUCU	-
ssc-miR-769-5p	6	UGAGACCUCUGGGUUCUGAGC	bta-miR-769
ssc-miR-7857-3p	7	AUUGUUCUCCAACCUGGCUCUU	bta-miR-7857-3p
ssc-miR-802	13	UCAGUAACAAAGAUUCAUCCU	eca-miR-9142

ssc-miR-874	2	CUGCCCUGGCCCGAGGGACCGAC	bta-miR-874
ssc-miR-99b	6	CACCCGUAGAACCGACCUUGCG	-
ssc-miR-885-5p	13	UCCAUUACACUACCCUGCCUC	bta-miR-885
ssc-miR-9	7	UCUUUGGUUAUCUAGCUGUAUGA	bta-miR-9-5p
ssc-miR-9810-3p	4	CGAGCACGCGGCCACUCUCCC	-
ssc-miR-769-3p	6	CUGGGAUCUCUGGGGUCUUGGUU	-
ssc-miR-92a	X	UAUUGCACUUGUCCCGGCCUG	bta-miR-92a
ssc-miR-92b-3p	4	UAUUGCACUCGUCCCGGCCUCC	-
ssc-miR-92b-5p	4	UAUUGCACUCGUCCCGGCC	bta-miR-92a
ssc-miR-935	6	CAGUUACCGCUUCCGCUACCG	-
ssc-miR-95	8	UUCAACGGGUAUUUAUUGAGC	bta-miR-95
ssc-miR-96-5p	18	UUUGGCACUAGCACAUUUU	bta-miR-96
ssc-miR-9-1	4	UCUUUGGUUAUCUAGCUGUAUGA	-
ssc-miR-9785-5p	AEMK02000499.1	CUGCCCACCUGGGUGAGAAGGA	-
ssc-miR-98	X	UGAGGUAGUAAGUUGUAUUGU	bta-let-7f
ssc-miR-9-2	2	UCUUUGGUUAUCUAGCUGUAUGA	-
ssc-miR-9820-5p	2	GAGGAGGAGGGAAGAAAGGC	-
ssc-miR-9841-3p	7	UCUAGCAUCGAGCACCCGCC	-
ssc-miR-9843-3p	8	UCUGUGAACUAGAAACCUC	-
ssc-miR-744	12	UGCGGGGCUAGGGCUAACAGCA	-
ssc-miR-9858-5p	4	UUCCUGAGUCGGACUGGGC	hsa-miR-1200
ssc-miR-9860-5p	7	UUGCCCGAGAGCUUGGACCG	-
ssc-miR-9851-3p	14	UGGCACCAGCACUGGCGGUGGC	-
ssc-miR-99a-5p	13	AACCCGUAGAUCCGAUCUUG	bta-miR-99a-5p
ssc-miR-7142-3p	2	UUUGUUGGCUCCUCUGAAGUGA	-
