# Supplementary material for: Investigation of salivary microRNAs as non-invasive biomarkers for weaning stress in piglets: A preliminary study
Source: PLoS One. 2026 Oct 13;21(10):e0360727. doi: 10.1371/journal.pone.0360727 (PMC13660905; doi:10.1371/journal.pone.0360727)
Supplement: Supplementary file 2 [file pone.0360727.s002.pdf]

S2 Table. List of novel miRNAs predicted from the saliva of suckling and weaning pigs.

Novel miRNA	CHR	Mature sequence (5'-3')	Known miRNA Names with the Same Seed
ssc-miR-novel-1	6	UAACACUGUCUGGUAACGAUGU	bta-miR-200a
ssc-miR-novel-2	3	UAAAGUGCUGACAGUGCAGA	bta-miR-20a
ssc-miR-novel-3	3	AACUGGCCCACAAAGUCCCGC	bta-miR-193a-3p
ssc-miR-novel-4	4	GCGCCGCGCUGGGAGCCCUGCGG	-
ssc-miR-novel-5	3	UAAUUUUAUGUAUAAGCUAG	eca-miR-590-3p
ssc-miR-novel-6	5	GUGCAUUGUAGUUGCAUUGCA	bta-miR-33a
ssc-miR-novel-7	9	AGGCAGUGUAAUUAGCUGAUUG	bta-miR-34b
ssc-miR-novel-8	5	UAAUACUGCCGGGUAAUGA	bta-miR-200c
ssc-miR-novel-9	AEMK02000452.1	GUGAAAUGUUUAGGACCAC	cfa-miR-203
ssc-miR-novel-10	13	UUCAAGUAACCCAGGAUAGGC	bta-miR-26a
ssc-miR-novel-11	12	GGGCAGGGUGAGUCGGCC	hsa-miR-1207-5p
ssc-miR-novel-12	2	GGGAGGCUUAGAAGCAGC	bta-miR-12010
ssc-miR-novel-13	7	GAAGAACCUUACCCAGCC	hsa-miR-548b-3p
ssc-miR-novel-14	11	UGACUCACUCUGUUGUGCAGC	-
ssc-miR-novel-15	3	CAAAGUGCUGUUCGUGCAGG	bta-miR-20a
ssc-miR-novel-16	17	ACUGGCCUUGGAGUCAGAAA	bta-miR-2439-5p
ssc-miR-novel-17	8	GUGGGGAGUUUGACUGGG	hsa-miR-3150a-3p
ssc-miR-novel-18	1	UCUGUGCCGAAAGCAUGGGGAC	eca-miR-6529
ssc-miR-novel-19	16	UGGCAGUGUAUUGUUAGCUGG	bta-miR-34b
ssc-miR-novel-20	X	CAUCCCUUGCAUGGUGGAGGG	bta-miR-188
ssc-miR-novel-21	3	AGAAGAGGACAGUGGAAC	bta-miR-10169-3p
ssc-miR-novel-22	1	GAGGGCUGGGCACGGGGG	hsa-miR-4446-3p
ssc-miR-novel-23	12	GAGGGCUGGGCACGGGGG	hsa-miR-4446-3p
ssc-miR-novel-24	3	CCUGGUGCCAAUGGCAAAG	hsa-miR-548q
ssc-miR-novel-25	3	CGGGCUGUGAUGAUUGGCGCAGGGG	hsa-miR-4675
ssc-miR-novel-26	AEMK02000452.1	ACCCGUCCCGUGCGUCCCCGG	bta-miR-1247-5p

ssc-miR-novel-27	X	AUGCACCUGGGCAAGGAUUC	cfa-miR-500
ssc-miR-novel-28	2	CGGGGCGGCAGGCUGAGCC	bta-miR-1777b
ssc-miR-novel-29	12	CAGUGCAAUAGUAUUGUCAAAGC	bta-miR-130a
ssc-miR-novel-30	16	AGGCAGUGUAUUGUUAGCUGGC	bta-miR-34b
ssc-miR-novel-31	12	CUUGGGAUGGGCGGGGGG	hsa-miR-6513-5p
ssc-miR-novel-32	5	GAAACUGUCAUACUGGAG	hsa-miR-548g-3p
ssc-miR-novel-33	16	AGGUUCUAACCCAGAUCC	hsa-miR-182-3p
ssc-miR-novel-34	11	UCCCAAGGGUUUGGCUGU	hsa-miR-6814-5p
ssc-miR-novel-35	AEMK02000452.1	UAUGUAAUAUGGUCCACGUC	bta-miR-380-3p
ssc-miR-novel-36	15	ACUGGAGGAAGGUGGGGA	cfa-miR-1843
ssc-miR-novel-37	15	UGGCUCAGCUGGAUAGAG	bta-miR-24-3p
ssc-miR-novel-38	9	GCUGGUGUAGUGGUAUCAUGCAAG	hsa-miR-7978
ssc-miR-novel-39	9	GUGGGGAAGAACUACAAGACAGC	bta-miR-6525
ssc-miR-novel-40	17	GGUGGGAGACUGGGAAGG	bta-miR-2897
ssc-miR-novel-41	12	GCUCUGUGAUGAACCCCAUGC	bta-miR-2364
ssc-miR-novel-42	2	GUGGGGAAGAACUACAAGACAGC	bta-miR-6525
ssc-miR-novel-43	3	AAAUCUCUGCAGGCAAAUGUGA	bta-miR-216b
ssc-miR-novel-44	2	CGGAGGGAGGGGGAGAGGGAGG	hsa-miR-1249-5p
ssc-miR-novel-45	7	UCUGAAAGUACUGGGGGCC	hsa-miR-4766-5p
ssc-miR-novel-46	17	GGGUGGGGGUCUGGGGGG	hsa-miR-92a-2-5p
ssc-miR-novel-47	12	UCCCUUCUCACUCCUAGCUGGUCA	hsa-miR-5088-3p
ssc-miR-novel-48	1	GGCCCUUACAUAGUCAGACUC	hsa-miR-938
ssc-miR-novel-49	13	GGGGCAGUGAGACUGUGGG	bta-miR-2450a
ssc-miR-novel-50	17	CGGUGGGGUGCAGUGCUGGAC	hsa-miR-92a-2-5p
ssc-miR-novel-51	6	AGGAAGAGGAUUCUAAGG	hsa-miR-6077
ssc-miR-novel-52	AEMK02000256.1	CAGCAAUGUGUAUCCUUA	hsa-miR-3908
ssc-miR-novel-53	12	ACCAUCGGAGGACGCGGUAGC	-
ssc-miR-novel-54	5	GGGGGUGGCGGCGGGGGG	hsa-miR-608
ssc-miR-novel-55	9	CUCCUGGCUGGCUCGCCA	-

ssc-miR-novel-56	1	GAGCGCCACCCCUGUCCC	-
ssc-miR-novel-57	13	UAAAUGAAAAGAGGAGUC	hsa-miR-3646
ssc-miR-novel-58	6	CCCCCGGCCGGGCCCGCCC	bta-miR-2890
ssc-miR-novel-59	15	AAGGGACUGGGCCCACCC	eca-miR-9024
ssc-miR-novel-60	9	UGGAAGAAGGGGUGCUGGC	-
ssc-miR-novel-61	13	AGGCUAACUGAGGCUCUC	-
ssc-miR-novel-62	AEMK02000682.1	GGGGGUGGCGGCGGGGGG	hsa-miR-608
ssc-miR-novel-63	12	CCACUGCCCCAGGUGCUGC	eca-miR-324-3p
ssc-miR-novel-64	1	CAAUUUAGGUUAAUGACUGUC	hsa-miR-32-3p
ssc-miR-novel-65	4	CACUGUUUAGGCAUUGUCU	cfa-miR-452
ssc-miR-novel-66	13	GCACCUGGGACUCGGCUCAGACACU	hsa-miR-6765-3p
ssc-miR-novel-67	11	CGGGGCCGGGGGUGGGGC	eca-miR-9056
ssc-miR-novel-68	3	GAACUCCGUGGACACUGCC	-
ssc-miR-novel-69	15	CAACUAGACUGUGAGCUUC	eca-miR-9139
ssc-miR-novel-70	14	UGCAGACUCGCUCUGCUCCCGGC	hsa-miR-4771
ssc-miR-novel-71	17	UGUGACUGUGCAGAGCUGGGGG	bta-miR-134
ssc-miR-novel-72	3	GUGCCUGCGGUCCUAGGCG	hsa-miR-6894-3p
ssc-miR-novel-73	2	GGGGGUGGGGGCCGGGCCGCC	hsa-miR-608
ssc-miR-novel-74	2	AGGGUGAAGGAACUAGGCA	-
ssc-miR-novel-75	12	GGAGCGGGCGGGCGGUCC	bta-miR-2302
ssc-miR-novel-76	3	CAUUGCACUUGUCUCGGUCUGA	bta-miR-92a
ssc-miR-novel-77	14	ACACUGCCGGGAAAAGCU	eca-miR-324-3p
ssc-miR-novel-78	X	GCGGCGGCGACUCUGGAC	-
ssc-miR-novel-79	2	CAGGGUUGGGCUGUUCGC	bta-miR-296-3p
ssc-miR-novel-80	17	ACAGCAGGUGGAGAUUCC	bta-miR-214
ssc-miR-novel-81	5	UCUCCAGUGAGACAGUCUC	hsa-miR-3925-3p
ssc-miR-novel-82	5	UCUGAGAUGUGACCUGGGCA	-
ssc-miR-novel-83	10	AGGACAGGGCUCCUGGCUCC	bta-miR-10162-5p
ssc-miR-novel-84	AEMK02000682.1	AGAGGAGCCGCGACAGAGCCG	eca-miR-1597

ssc-miR-novel-85	13	GGGACUGAGACACGGCCA	hsa-miR-4740-5p
ssc-miR-novel-86	AEMK02000264.1	GGGGGUGUAGCUCAGUGGUAGAG	bta-miR-2438
ssc-miR-novel-87	12	CCCUGAGGCAGAGCCUCCUC	hsa-miR-544b
ssc-miR-novel-88	12	GGGACUGAGACACGGACC	hsa-miR-4740-5p
ssc-miR-novel-89	3	AUUUGAGAGGAGCUGCCC	-
ssc-miR-novel-90	X	GAGACGUGAUGACGAGUC	hsa-miR-6863
ssc-miR-novel-91	13	CAAGAGGCUGAUGAGGAC	-
ssc-miR-novel-92	2	UUUGUUAGAGGAAUGAGC	-
ssc-miR-novel-93	18	UGCUGGAGGCCGCCUAUCC	eca-miR-8929
ssc-miR-novel-94	12	UUCCUGUGGCUGGGAAGCUC	-
ssc-miR-novel-95	5	GACUCGACUGGUGUGCGGACAC	-
ssc-miR-novel-96	6	CCUCCUGCACUGCCUCCCCAG	bta-miR-12051
ssc-miR-novel-97	14	CUAGGUUGGGUGUGGAAGAG	bta-miR-2369
ssc-miR-novel-98	16	UGGUCUAGCGGUUAGGACA	hsa-miR-601
ssc-miR-novel-99	6	GGUGGCACAGGCCAGGGGG	hsa-miR-891a-3p
ssc-miR-novel-100	9	UUCAGUGAUGUCAGCAGC	hsa-miR-4276
ssc-miR-novel-101	2	AGCACUUACGGAUUCUGGGGG	-
ssc-miR-novel-102	1	GAAACUGGUGGGCUUGAG	bta-miR-2285aa
ssc-miR-novel-103	11	GCAUUCUAAUGAGACUGC	-
ssc-miR-novel-104	18	AGUGAAAGAGAAUCUGAAAC	-
ssc-miR-novel-105	7	CCCUGGGCCAGGGUGUCG	hsa-miR-939-3p
ssc-miR-novel-106	16	CAGGAAAAGGAUUGGCUC	-
ssc-miR-novel-107	14	GGUCAUGCUGGGCACUCUU	-
ssc-miR-novel-108	13	AAGGAGCUGAGGACGGAGA	bta-miR-28
ssc-miR-novel-109	6	GCUAAGUGGCAAAGGAUG	-
ssc-miR-novel-110	X	UGUUCAUUGCCUCCUCGGCCGAC	hsa-miR-6853-3p
ssc-miR-novel-111	AEMK02000697.1	AUGGUGAAUGCCUUGGCAG	-
ssc-miR-novel-112	1	CGGGCUGUGGCGGGAGGGU	hsa-miR-4675
ssc-miR-novel-113	11	CUUGUCCUUAGUUGCCAUG	bta-miR-2355-3p

ssc-miR-novel-114	AEMK02000256.1	GCCGGGUGGUCUCCUGGG	eca-miR-9152
ssc-miR-novel-115	13	GGCUGGGCUGUGAUGGGGAAAGA	-
ssc-miR-novel-116	14	GUGGGAGGCGGGGGAAGGA	bta-miR-30b-3p
ssc-miR-novel-117	17	GUUGGGAGACUUUGAAAU	hsa-miR-887-5p
ssc-miR-novel-118	6	UCUGCACCGGGGCUUUGAGGG	eca-miR-9143
ssc-miR-novel-119	X	GAGGAACCUUACCCAGGC	hsa-miR-3914
ssc-miR-novel-120	17	CUGCAAGGAGGAGGAAGGCGC	eca-miR-9049
ssc-miR-novel-121	1	ACCAGGGCUGCUCUCAGGC	hsa-miR-4685-5p
ssc-miR-novel-122	11	GUUUGGUUGUGGUUCUUUGAGGCGG	bta-miR-133c
ssc-miR-novel-123	4	GCCGAGGAGGAGGAGGCAGG	-
ssc-miR-novel-124	7	GUGCCUGCAAGGCUGGAG	hsa-miR-6894-3p
ssc-miR-novel-125	9	UGUGGGGGAGGGGGAAGG	hsa-miR-4447
ssc-miR-novel-126	3	AGAGUAGCCAAGUCUGGA	-
ssc-miR-novel-127	9	CAUUCAGAUCUUGCUGUC	hsa-miR-892c-5p
ssc-miR-novel-128	7	CCCUUGGAAAAGCCAUGCUC	eca-miR-9099
ssc-miR-novel-129	5	CAUUCAGAUCUUGCUGUC	hsa-miR-892c-5p
ssc-miR-novel-130	AEMK02000318.1	CAGCAAUGUGUAUCCUUA	hsa-miR-3908
ssc-miR-novel-131	X	UGAGUACGAGCAACUCUCC	cfa-miR-8856
ssc-miR-novel-132	1	ACGCUGGGGUUAUAGGAU	-
ssc-miR-novel-133	2	CCUUGAUGGUCAUAAUGC	-
ssc-miR-novel-134	16	CUGGGCUGUGUUUGUGCUCA	hsa-miR-3620-5p
ssc-miR-novel-135	14	CCAGGAGAGGAGAAGGGA	hsa-miR-4768-3p
ssc-miR-novel-136	13	GGUGGGAGACUGGGAAAG	bta-miR-2897
ssc-miR-novel-137	6	GCAGGCAAAUCCACGGGG	hsa-miR-4742-5p
ssc-miR-novel-138	X	AAGAGUGAAAAAUCUCUC	hsa-miR-5586-3p
ssc-miR-novel-139	2	GUGGCUCUGUCAGAAUUCGCUC	bta-miR-12004
ssc-miR-novel-140	8	AGAGUUUGCUCAUGGCUC	-
ssc-miR-novel-141	5	AGAGAACCUGAAACCCUG	cfa-miR-589
ssc-miR-novel-142	3	GGUGCCUGACUCAGCCAGAGG	hsa-miR-550a-5p

ssc-miR-novel-143	13	AUUCUGUUAGAAAAAUGCAAGA	hsa-miR-4652-3p
ssc-miR-novel-144	1	AGAAUUAUCUGGGAAGGUA	-
ssc-miR-novel-145	9	CUGGUCACCUGGGCCAGGCCCUAC	hsa-miR-5587-5p
ssc-miR-novel-146	13	GAGGAAUUGGCUGGGAAGCU	-
ssc-miR-novel-147	2	GGUACUGAGACAGGGACC	-
ssc-miR-novel-148	10	UCCGUGGCUUGGGAUGGGC	hsa-miR-132-5p
ssc-miR-novel-149	2	UCUUCCAGGAAAAGCCAC	-
ssc-miR-novel-150	AEMK02000452.1	CCGGGCGCUGCCGGAGGGGGC	hsa-miR-6499-5p
ssc-miR-novel-151	X	UCUUGUGAUGUCAGCAGC	bta-miR-2356
ssc-miR-novel-152	18	GAUGGAGGGACGGAGAAA	hsa-miR-4802-5p
ssc-miR-novel-153	AEMK02000452.1	GUGAAAUGUUUAGGACCACUAG	cfa-miR-203
ssc-miR-novel-154	14	GGGGAUGUAGCUCAGUGGUAGAG	-
ssc-miR-novel-155	3	ACUCUGGUGUAGGCCCGC	hsa-miR-6764-3p
ssc-miR-novel-156	12	ACUCUGGUGUAGGCCCGC	hsa-miR-6764-3p
ssc-miR-novel-157	14	GUUUCAUACGUAGCAGAGCAGCUC	-
ssc-miR-novel-158	13	UGUGGUUGUGGCAUAGACC	eca-miR-9035
ssc-miR-novel-159	15	GUUCGAUCCCUACCUGGC	-
ssc-miR-novel-160	1	GUUCGAUCCCUACCUGGC	-
ssc-miR-novel-161	7	UCUGGUUCUGUGACCCUGCC	-
ssc-miR-novel-162	11	UGACUCACUCUGUUGUGCAG	-
ssc-miR-novel-163	12	CCACCGUCGGUUCUCUGGUGUC	-
ssc-miR-novel-164	9	AUGGAGGUCUCUGUCUGGCU	bta-miR-12054
ssc-miR-novel-165	15	GGCGGGGCCGGGGGCGGGGC	bta-miR-744
ssc-miR-novel-166	7	GCCGGCGCGCCCCCGGCC	bta-miR-10170-5p
ssc-miR-novel-167	2	CGGGGCGGCAGGCUGAGCCUC	bta-miR-1777b
ssc-miR-novel-168	1	GGCCCUUACAUAGUCAGAC	hsa-miR-938
ssc-miR-novel-169	14	CAGUGCAAUGAUAUUGUCAAAGC	bta-miR-130a
ssc-miR-novel-170	X	GGCGGGCGGGGCCGGGGG	hsa-miR-4767
ssc-miR-novel-171	AEMK02000452.1	UUGAACUGUUAAGAACCAC	hsa-miR-203b-3p

ssc-miR-novel-172	13	GCCCUCGUAGCUCAGUGG	hsa-miR-4655-3p
ssc-miR-novel-173	17	GCUGGACUGGGCCGGACUGGGC	bta-miR-378
ssc-miR-novel-174	5	CUGAGGGCUGGGCAUGGGGG	hsa-miR-1226-5p
ssc-miR-novel-175	3	CCAGUGAUCAGGUUACGAUGGAU	-
ssc-miR-novel-176	17	UCUGGGGUAACCUCUGAGU	bta-miR-6523a
ssc-miR-novel-177	8	GGUGGGGAGUUUGACUGGG	bta-miR-491
ssc-miR-novel-178	3	AGGCUUGGCUCUGAGGGC	eca-miR-9025
ssc-miR-novel-179	15	ACACUUGGGGAGACUGAG	hsa-miR-3614-5p
ssc-miR-novel-180	10	GUCAGGAUGGCAGAGGGG	eca-miR-8919
ssc-miR-novel-181	6	GCUGGUGGGUGGUGGGUGG	hsa-miR-4456
ssc-miR-novel-182	12	CAGGCAGUUGGAAAGGAGG	hsa-miR-34b-5p
ssc-miR-novel-183	3	AGGGGCUGACGCCUGCCC	hsa-miR-185-3p
ssc-miR-novel-184	8	GGGAGGAUAGGAAGCUGC	hsa-miR-6894-5p
ssc-miR-novel-185	2	GCAGCACUCUGUCCCCCCCAG	hsa-miR-383-3p
ssc-miR-novel-186	6	UUAUUAUUUUUCUUUUACUGC	hsa-miR-4795-3p
ssc-miR-novel-187	3	CAUAGCUCAGUGGUAGAG	hsa-miR-7705
ssc-miR-novel-188	18	AACUCUGGUGGAGGCCCU	hsa-miR-514a-5p
ssc-miR-novel-189	7	UGAGGUUUGAGGAUUGCUC	bta-miR-329b
ssc-miR-novel-190	13	GCCAGUGAAAUACCACUAC	bta-miR-2375
ssc-miR-novel-191	8	AUGGCUCUGAGGACUGGGC	bta-miR-12004
ssc-miR-novel-192	13	GAGGGCUGGGGGUAGGGC	hsa-miR-4446-3p
ssc-miR-novel-193	6	AGAGACAGGCGGCUGUAAC	hsa-miR-4524a-3p
ssc-miR-novel-194	11	GCGGGGCCAGGGGUGGGG	-
ssc-miR-novel-195	3	GCUGGCAGGGCCCCCACCC	eca-miR-9171
ssc-miR-novel-196	13	UUUCCUGUUUACUCUGCC	hsa-miR-7152-5p
ssc-miR-novel-197	6	AGACAUUCGCCUCCUCGCUGAC	-
ssc-miR-novel-198	5	GAGUAGGUGGCUGCCUUC	bta-miR-2341
ssc-miR-novel-199	13	AAGGAGCUGAGGACGGAGAAGG	bta-miR-28
ssc-miR-novel-200	AEMK02000158.1	AUUGGACUUAUUUUCAUUU	hsa-miR-4529-3p

ssc-miR-novel-201	3	CAGCUGGCUAGAGCAUCU	eca-miR-9027
ssc-miR-novel-202	6	GCCGCCGCCGCCGCCGCC	-
ssc-miR-novel-203	9	GGAAGAAGGGGUGCUGGC	hsa-miR-3185
ssc-miR-novel-204	9	GGGGAAGAACUACAAGACAGC	cfa-miR-8808
ssc-miR-novel-205	15	GCUGGACUGGAGCUGAGG	bta-miR-378
ssc-miR-novel-206	2	GUUGGAUUUAUGAAAGACG	hsa-miR-5004-3p
ssc-miR-novel-207	15	AGGAAGAGAAUUCUAAGG	hsa-miR-6077
ssc-miR-novel-208	14	CCAAACCAGUUGUGCCUGUAG	hsa-miR-6715a-3p
ssc-miR-novel-209	5	CGGGCUGCCUCUCCCCGCAG	hsa-miR-6829-5p
ssc-miR-novel-210	6	CGCGGCGGUUGUGGGCUGC	bta-miR-11975
ssc-miR-novel-211	1	CCCGGAAAUCCAAGGACUGUA	-
ssc-miR-novel-212	2	GGGGAAGAACUACAAGACAGC	cfa-miR-8808
ssc-miR-novel-213	1	CGCCCGCCGCCCGCCCCGC	hsa-miR-4707-3p
ssc-miR-novel-214	10	GCGGGCCCACGGGGGCCC	-
ssc-miR-novel-215	15	GCCGGCGGGGGCCUCGGU	-
ssc-miR-novel-216	9	CAGGCUGAGGAUGUGAGAAC	hsa-miR-3929
ssc-miR-novel-217	3	GGGGGCCGGCGGCGGCGGC	eca-miR-9056
ssc-miR-novel-218	16	GACAUGGAUGGGGGUGAAA	bta-miR-194b
ssc-miR-novel-219	12	GGGGGCCGGCGGCGGCGGC	eca-miR-9056
ssc-miR-novel-220	15	GCGGGGGUGGGCGGGCGG	hsa-miR-4787-5p
ssc-miR-novel-221	13	CACUGUACUCCAGCCUGGGCAA	bta-miR-2384
ssc-miR-novel-222	7	GCUGGCCGAGGCGCCGCC	-
ssc-miR-novel-223	7	CCCCGCCUCCUCUCGCCGCC	-
ssc-miR-novel-224	13	GGGGUGGGCGCCGGCGGC	hsa-miR-6756-5p
ssc-miR-novel-225	3	CGGCGGGCCGCCGCGCGC	bta-miR-2899
ssc-miR-novel-226	12	GGCAGGGUGAGUCGGCCC	-
ssc-miR-novel-227	13	AGCAUUCGGCUCGCCGCGUCG	cfa-miR-8852
ssc-miR-novel-228	4	CGGGGUGUGGGGAGGGCC	bta-miR-2438
ssc-miR-novel-229	10	CGGAGCCUGGAGGGCUGC	bta-miR-2904

ssc-miR-novel-230	16	CGGGGCCGGACUGGGCGGGG	eca-miR-9056
ssc-miR-novel-231	12	GGGCGCGGCGGCCGCUGC	eca-miR-675
ssc-miR-novel-232	15	CCAAGGCCCGGCCUCGCCU	hsa-miR-3922-5p
ssc-miR-novel-233	5	GCGGGGGUGGCGGCGGGGGGC	hsa-miR-4787-5p
ssc-miR-novel-234	12	GCCAAAGGGGGCAGACUG	-
ssc-miR-novel-235	18	GAAACUGUGGAGCUGGAG	hsa-miR-548g-3p
ssc-miR-novel-236	14	CGGCGGCGCGGGCCCCGGGC	hsa-miR-4467
ssc-miR-novel-237	AEMK02000489.1	UGUGUGUGGGCGCCGGACGC	hsa-miR-6867-5p
ssc-miR-novel-238	6	GCAGGGGUAGAGCACUUCC	-
ssc-miR-novel-239	8	GGUGGCGCGCGCCUGUAGUCCC	-
ssc-miR-novel-240	1	UGUCCCACAACCCGCACCCGCC	hsa-miR-4433a-5p
ssc-miR-novel-241	12	GGUUUGAUUCCCGGCUGG	-
ssc-miR-novel-242	14	CAGUGGAUAGAGCUCUGU	hsa-miR-3681-5p
ssc-miR-novel-243	9	GGGCGCGGCGGCCGCUGC	eca-miR-675
ssc-miR-novel-244	3	AGGGCCUGAUGAGGGGGG	hsa-miR-2861
ssc-miR-novel-245	X	CACUGGUUUCCUGGAGCAGCAGGU	cfa-miR-8888
ssc-miR-novel-246	12	CGCGCUGCAGCCUGAGCCCUCG	eca-miR-8992
ssc-miR-novel-247	16	GUUCGAUCCCUACCUGGC	-
ssc-miR-novel-248	2	CGCAGCCGUCUCCCUGGGCC	-
ssc-miR-novel-249	AEMK02000695.1	UGUGUGUGGGCGCCGGACGC	hsa-miR-6867-5p
ssc-miR-novel-250	3	GGGCGGGGGCCUCGGGAA	hsa-miR-663a
ssc-miR-novel-251	2	GCAGGCAGGCGGGCAGAC	hsa-miR-4514
ssc-miR-novel-252	12	CGUCCUCUCCCUCCCUCC	hsa-miR-3909
ssc-miR-novel-253	9	CACUGGAGUUUUGUUUCAACAU	-
ssc-miR-novel-254	2	UUUCCUGGCUGGUGCACC	-
ssc-miR-novel-255	15	GUUCAAUCCCGGCCGGGC	-
ssc-miR-novel-256	AEMK02000682.1	GCGGGGGUGGCGGCGGGGG	hsa-miR-4787-5p
ssc-miR-novel-257	3	GUGCCUGCGGUCCUAGGCGCG	hsa-miR-6894-3p
ssc-miR-novel-258	9	AGCCUGGGGCUCUGGACA	hsa-miR-661

ssc-miR-novel-259	18	CCUGGUGGGUGGUGGGUGA	hsa-miR-4456
ssc-miR-novel-260	5	CCCAGGCAGUCAGACUGCA	hsa-miR-4645-5p
ssc-miR-novel-261	14	GUGGCUUAUGAAUCUGGC	-
ssc-miR-novel-262	4	CCAGGGCCGGCAGAAGCUGUUU	bta-miR-12039
ssc-miR-novel-263	3	CAUUGCACUUGUCUCGGUC	bta-miR-92a
ssc-miR-novel-264	16	GAGUGAGACAGAGGAGAG	-
ssc-miR-novel-265	1	CCCCUGUCCUCCAGGAGCUC	bta-miR-339a
ssc-miR-novel-266	5	GGGUGGGGGUCUGGGGGG	hsa-miR-92a-2-5p
ssc-miR-novel-267	10	GGGGCUGUAGCUCCGAUGG	hsa-miR-4675
ssc-miR-novel-268	2	UCUGACACCCCCCCACCCAC	hsa-miR-6730-3p
ssc-miR-novel-269	8	CGGGGCCGGCGGGGGCAG	eca-miR-9056
ssc-miR-novel-270	3	ACGGAGGGUGUGGGCUGACUCC	bta-miR-10185-5p
ssc-miR-novel-271	9	AGGAAGCCCUGGAGGGGC	bta-miR-671
ssc-miR-novel-272	2	CAGUGAACAGGACCUUGCCCC	-
ssc-miR-novel-273	18	GGCCGGGCUUCUGCAGGGUG	cfa-miR-8834a
ssc-miR-novel-274	2	ACUGCUGGGCCUGGCAUGCC	hsa-miR-3692-5p
ssc-miR-novel-275	X	UCUGGGGAAGGGACCUGGGAA	hsa-miR-4667-5p
ssc-miR-novel-276	8	GGCCGGGCGGAGCGGUCGUC	cfa-miR-8834a
ssc-miR-novel-277	1	GUCCUCUCCUGGGCACCU	hsa-miR-7111-3p
ssc-miR-novel-278	2	UCUGCAGGUCCAGGAGGAGC	bta-miR-10179-5p
ssc-miR-novel-279	3	GGGCUGUGAUGAUUGGCGCAGGGG	hsa-miR-7974
ssc-miR-novel-280	AEMK02000695.1	AGGGACCGGGGUCAGGCGG	bta-miR-2887
ssc-miR-novel-281	6	UGCCCCACCCGCAGGGCCUGU	hsa-miR-4758-3p
ssc-miR-novel-282	13	GUUGGACCCUGUGAAACUGCC	eca-miR-9180
ssc-miR-novel-283	2	AGGGGCGGGCGCGGGCGC	bta-miR-1777b
ssc-miR-novel-284	14	GUGGGGCUGGAGCAGGUCC	bta-miR-2374
ssc-miR-novel-285	18	GGUGCAGUGGGUUAAGGA	hsa-miR-143-5p
ssc-miR-novel-286	8	AGGCUGAGCUGUGAUGGGU	bta-miR-2338
ssc-miR-novel-287	6	AGGUCCCUGUUCAGGCGC	-

ssc-miR-novel-288	2	CAGGGCUGGGCUCGGGGG	hsa-miR-4446-3p
ssc-miR-novel-289	18	UGCUGGUGGCCAUGGCAGG	bta-miR-138
ssc-miR-novel-290	6	GCAGUAAAAGAAAAAAUAA	bta-miR-545-5p
ssc-miR-novel-291	5	CGGGAAAGUUGGUCAAACCC	hsa-miR-3153
ssc-miR-novel-292	17	GCCAUGCGCUCCUGGCCU	-
ssc-miR-novel-293	1	AUUGGCUGGAAAUGGACA	-
ssc-miR-novel-294	2	GUGAGGGAGGAGGGGGAG	hsa-miR-6834-5p
ssc-miR-novel-295	AEMK02000682.1	GGCGGGGCCUUGGCCGAC	bta-miR-744
ssc-miR-novel-296	17	GUGGGGCCAUCGGGCUGCCUGG	hsa-miR-1227-5p
ssc-miR-novel-297	6	CACUGGCCCGAGGCCCGGC	bta-miR-193a-3p
ssc-miR-novel-298	3	UGCUUCCUUCUGAACAUUUUG	hsa-miR-516b-3p
ssc-miR-novel-299	8	CCUGGGAAGGAAGGAGUAAGGC	bta-miR-2447
ssc-miR-novel-300	7	UUGAAAGAGCCUGAUGCAC	-
ssc-miR-novel-301	6	GCCCCGCUCUGAGGACUGCGC	eca-miR-8994
ssc-miR-novel-302	12	UGAGGGACGGAAGGGGGAG	hsa-miR-3126-5p
ssc-miR-novel-303	12	UCUGUUUACAACACACCCAACA	-
ssc-miR-novel-304	9	AGGUCAAGCUGGGCACUC	eca-miR-1898
ssc-miR-novel-305	13	UAGGGUCUAGGUCGCAGAUACA	hsa-miR-1182
ssc-miR-novel-306	6	UUCCACUGUCUCUGCUGC	hsa-miR-6509-3p
ssc-miR-novel-307	4	GAGCAGGUUCUCUUGGUC	-
ssc-miR-novel-308	6	UGCCAAGGGUGGGGUUGG	-
ssc-miR-novel-309	3	UCUGGUCUCCCUCACAGGAC	hsa-miR-3663-5p
ssc-miR-novel-310	3	GGGGCCUCCCAGCACCCGG	eca-miR-9114
ssc-miR-novel-311	13	CAGUGGUUGAGGCCUGCU	eca-miR-1244
ssc-miR-novel-312	6	CUGGGCUCGGGGGUCCCA	hsa-miR-4674
ssc-miR-novel-313	5	AACUAAGUGGGAAAGGAGA	-
ssc-miR-novel-314	6	GAGGAUUGGCUCUGAGGA	-
ssc-miR-novel-315	14	GGCAGAAGGGGGCUUGAC	hsa-miR-298
ssc-miR-novel-316	5	GACCUGGGAGUGUUGAGAAC	hsa-miR-7851-3p

ssc-miR-novel-317	10	ACUGGGGUAACCUCUGAGG	bta-miR-6523a
ssc-miR-novel-318	12	GUGAGGGGACUGGGGUGGGC	bta-miR-2422
ssc-miR-novel-319	15	UAUCUGGUUGAUUCUGCCA	-
ssc-miR-novel-320	8	AGGAGGAGGAGGAGGACG	bta-miR-10176-5p
ssc-miR-novel-321	3	CCCCUGCCAGGGAGAGGGGAC	hsa-miR-6752-3p
ssc-miR-novel-322	AEMK02000682.1	AUCCCACUUCUGACACCA	hsa-miR-3156-3p
ssc-miR-novel-323	3	GCUCCCAACCCCUCCAGCUCU	bta-miR-150
ssc-miR-novel-324	12	GUUCGAUCCCCUGGCCAA	-
ssc-miR-novel-325	3	CAGGCUCUCCAUGCUGGGAU	bta-miR-8550
ssc-miR-novel-326	2	UGAGAUGAGAGCCCUUGGCC	bta-miR-143
ssc-miR-novel-327	AEMK02000258.1	UCAUCCCCGAACUACGGCUGC	hsa-miR-6729-3p
ssc-miR-novel-328	3	GCUGGGCGAGCAUCUGCC	hsa-miR-4486
ssc-miR-novel-329	2	GGGCUGAGGCGGGCUGUGCCC	bta-miR-2338
ssc-miR-novel-330	4	GGUCACAGGUUCGAUUCC	hsa-miR-12122
ssc-miR-novel-331	17	CGGGGCGGGGGCCCGGGC	bta-miR-1777b
ssc-miR-novel-332	14	GAGGCGGGGUGAGGACCA	bta-miR-2419-3p
ssc-miR-novel-333	1	UCAGCUUCGGGCCUAGAUCC	-
ssc-miR-novel-334	6	CUGGCUGGCCGGUCUGCU	hsa-miR-6842-3p
ssc-miR-novel-335	6	GUUCAAAUCCUGCCUCCGC	hsa-miR-607
ssc-miR-novel-336	7	UCAGCUGGCUAGAGCACA	bta-miR-763
ssc-miR-novel-337	5	UCCUGGGGCUGGAGCAGG	eca-miR-8970
ssc-miR-novel-338	13	GGGGGAGGGCGAGGCGGA	hsa-miR-6825-5p
ssc-miR-novel-339	15	ACAGUGGUAGAGCACUCCC	-
ssc-miR-novel-340	2	GGCUGGGGCCUGGGCUCCUA	hsa-miR-4731-5p
ssc-miR-novel-341	7	UGGGAGGUCUUGUGGGGC	hsa-miR-6799-5p
ssc-miR-novel-342	3	GGGGCUGGAUGACCCGAGG	eca-miR-8953
ssc-miR-novel-343	14	CCCUGUCUCACUGUCUCCUUC	hsa-miR-4421
ssc-miR-novel-344	2	GAGGAGGCUGUCUAACAAG	hsa-miR-4695-5p
ssc-miR-novel-345	17	CCCGUGGCUGUGGGCUCCA	hsa-miR-132-5p

ssc-miR-novel-346	13	GGGGUGGGGCAGUGGGAG	hsa-miR-6756-5p
ssc-miR-novel-347	14	UGCACUGGGUCAGGGCAGGGC	-
ssc-miR-novel-348	12	ACUGGACUUGGAGUCAGAAG	bta-miR-378
ssc-miR-novel-349	2	CUCCCCCCCUCCCCGGCC	-
ssc-miR-novel-350	17	UUGGCUGGGAGUCGGGGGCCC	hsa-miR-6842-3p
ssc-miR-novel-351	12	CUACUCUGUCCCCACCUCCAG	bta-miR-2319a
ssc-miR-novel-352	4	GGAGACAGGUGCUAAGAGCUC	hsa-miR-4524a-3p
ssc-miR-novel-353	3	UAUAGCUCAGGGGUAGAGCA	hsa-miR-7705
ssc-miR-novel-354	12	CCUGGGGGUGAGAGGGUGUCCCC	hsa-miR-637
ssc-miR-novel-355	1	CCUGGGUGCAUUUGCAGCGAGA	eca-miR-8931
ssc-miR-novel-356	11	UCUUGGCUGUCUGAGCUC	-
ssc-miR-novel-357	12	AAGGUCCCAAGGGUUGGGA	bta-miR-12015
ssc-miR-novel-358	15	GAGGAAGGUGAGGAUGAC	hsa-miR-4476
ssc-miR-novel-359	12	CCCCGCCCCCGCCAGGACCA	hsa-miR-7108-3p
ssc-miR-novel-360	11	GGGAGCAGAGCUGAAAAAGC	hsa-miR-6081
ssc-miR-novel-361	11	UGUGUGGCUAAGUGGUAGAU	hsa-miR-644a
ssc-miR-novel-362	4	UGGCCUGUGCUGCUGCUGC	hsa-miR-6735-3p
ssc-miR-novel-363	11	CAGGAAACAAAGAGCAGC	bta-miR-12047
ssc-miR-novel-364	2	UCUGGGCUUUAUUUCAUA	oar-miR-134-3p
ssc-miR-novel-365	AEMK02000328.1	GGUGGAGGGAAGCUGUAGCAGC	hsa-miR-7847-3p
ssc-miR-novel-366	X	CAUGCCUUGAGUGUAGGACA	bta-miR-532
ssc-miR-novel-367	13	UCUGGCCUUGUGCACACAGGG	bta-miR-2439-5p
ssc-miR-novel-368	2	CCCCUGCAGGCCCUGACCAC	bta-miR-2331-3p
ssc-miR-novel-369	17	CUCAGUUGGCAGAGCACA	-
ssc-miR-novel-370	1	GGAGGCAGAGGACUUGGC	hsa-miR-1827
ssc-miR-novel-371	13	AGGGAAUGAGGAUCUGAUG	hsa-miR-6716-5p
ssc-miR-novel-372	2	UGGUGGUUAAGAUUCGGCGC	hsa-miR-876-3p
ssc-miR-novel-373	2	UCUGACCUUUGCCUCCCAG	oar-miR-3956-3p
ssc-miR-novel-374	14	UGGGUGGAGCGUAGACCUGCC	bta-miR-10171-3p

ssc-miR-novel-375	X	UAUGGAGGGUGACUGAGGAUAG	hsa-miR-4802-5p
ssc-miR-novel-376	6	UCCUGGGUUUCAGGGUAGGUC	hsa-miR-6742-3p
ssc-miR-novel-377	10	AUCCCUGACCUGGGGAAC	eca-miR-670
ssc-miR-novel-378	10	CUGGGGGCUGGGCUGAGGGG	bta-miR-2454-5p
ssc-miR-novel-379	8	AGGGAGAGGCCUGAGGCC	hsa-miR-6731-5p
ssc-miR-novel-380	6	UGUUCUCUUCUGUCACACUC	hsa-miR-2117
ssc-miR-novel-381	18	AGGUGAGGCUCUGCAGCCUGGUC	hsa-miR-4710
ssc-miR-novel-382	15	GUCGGGCUGGACGAGGGC	hsa-miR-3944-3p
ssc-miR-novel-383	AEMK02000408.1	GCCUGCCUGUGGUGAACUCUCUGCC	hsa-miR-6747-3p
ssc-miR-novel-384	11	GACAGAUGGACACGCCCUGACG	bta-miR-1721
ssc-miR-novel-385	AEMK02000278.1	GCCUGCCUGUGGUGAACUCUCUGCC	hsa-miR-6747-3p
ssc-miR-novel-386	AEMK02000682.1	GGAGGCAGAGGACUUGGC	hsa-miR-1827
ssc-miR-novel-387	4	GGAGUGGAGCCUGUGGCU	-
ssc-miR-novel-388	11	UGAUGGAGAGCUGAGGAGGCGC	bta-miR-2417
ssc-miR-novel-389	16	GAGGGACAGGAAGAUGGG	eca-miR-7667
ssc-miR-novel-390	6	CUGGAGGGCUUGUUUUGCUUCU	hsa-miR-6515-5p
ssc-miR-novel-391	12	CCGCUCCCUCCCACUGCCCAG	-
ssc-miR-novel-392	3	CUGGGGGUGGGACCAGAGCAGC	bta-miR-2285ah-5p
ssc-miR-novel-393	AEMK02000394.1	GCCUGCCUGUGGUGAACUCUCUGCC	hsa-miR-6747-3p
ssc-miR-novel-394	15	GAAGGAGAGGAGGGGGUC	hsa-miR-4428
ssc-miR-novel-395	1	GAUGGAGGAGGUGGAGGCG	hsa-miR-4802-5p
ssc-miR-novel-396	AEMK02000657.1	GCCUGCCUGUGGUGAACUCUCUGCC	hsa-miR-6747-3p
ssc-miR-novel-397	17	UCUGUAUUGUGAAUGGGGGGA	hsa-miR-4742-3p
ssc-miR-novel-398	AEMK02000361.1	GCUCAGUGGUAGAACAUUUGAC	-
ssc-miR-novel-399	X	GCCAGGAGACCCGGGUUC	cfa-miR-665
ssc-miR-novel-400	4	GCUGGCAGGGCCCCCACCC	eca-miR-9171
ssc-miR-novel-401	9	GGUUUGAUCCUGGCUCAG	-
ssc-miR-novel-402	1	ACAGGAGGAAGGUGGGGA	-
ssc-miR-novel-403	16	GGGGGUGGGAGAGGGGGC	hsa-miR-608

ssc-miR-novel-404	2	GGCACCUGGCUCGCCGUCCUCCC	bta-miR-11974
ssc-miR-novel-405	1	GGGGGUGGACGGCGUGGGA	hsa-miR-608
ssc-miR-novel-406	AEMK02000452.1	GAAUGUUGCUCGGUGAACCCC	eca-miR-409-3p
ssc-miR-novel-407	1	GGCCGGGCCCGGAGGCCGGGC	cfa-miR-8834a
ssc-miR-novel-408	1	UGUCUCUGCCCCUGCCCC	hsa-miR-593-3p
ssc-miR-novel-409	2	GGCUCCCUCCACCCGCCC	hsa-miR-7106-3p
ssc-miR-novel-410	7	UUGCUCUGCUGUGUUCUGUUC	hsa-miR-5691
ssc-miR-novel-411	14	AGGGUGUUGGUAGGUCUG	hsa-miR-609
ssc-miR-novel-412	6	GACUGAACCAGGCUCUUG	hsa-miR-4724-5p
ssc-miR-novel-413	5	AUUGGAGGGCAAGUCUGG	-
ssc-miR-novel-414	6	CCCAGGGCGAGGCUCAUC	hsa-miR-4685-5p
ssc-miR-novel-415	9	AGAGCCGAGAAUGGGAGAAGAG	hsa-miR-4537
ssc-miR-novel-416	5	GAAACUGUGAGGCUGGAG	hsa-miR-548g-3p
ssc-miR-novel-417	3	GAAGGCAGGUCUCUGGGC	hsa-miR-3714
ssc-miR-novel-418	10	GCUGUGGGUAGGGGUGAA	hsa-miR-134-3p
ssc-miR-novel-419	9	GGAAGAAGGGGUGCUGGCU	hsa-miR-3185
ssc-miR-novel-420	1	AGGGCUGGGCAUGGGGGUC	eca-miR-8953
ssc-miR-novel-421	4	ACUGGCUCUGAGGGUUGGG	bta-miR-149-5p
ssc-miR-novel-422	7	AAUGAUUGGGGUGAAGUC	cfa-miR-507a
ssc-miR-novel-423	7	AGCCUGGGAGUCAGACUGC	hsa-miR-661
ssc-miR-novel-424	1	AGGGUGUUGGCUUAGAAG	hsa-miR-609
ssc-miR-novel-425	14	CUGAGCUGGGAGAGCACC	bta-miR-2410
ssc-miR-novel-426	6	GGGGGCCGAUGAAGGACG	eca-miR-9056
ssc-miR-novel-427	5	AUCCUGGGGCUGGAGCAGG	bta-miR-1343-3p
ssc-miR-novel-428	11	GACUCUAGAGAAACUGCC	hsa-miR-5684
ssc-miR-novel-429	5	GACUGAGCAUCCUGUGAC	cfa-miR-8890
ssc-miR-novel-430	17	GAAGAACCUUACCAGGAG	hsa-miR-548b-3p
ssc-miR-novel-431	X	AUGUGGGGAAGGAGAAGG	hsa-miR-6796-5p
ssc-miR-novel-432	11	AAAAGAACUGUGAGAAGA	hsa-miR-3133

ssc-miR-novel-433	7	UCUGGUGAUGAGGGAGA	hsa-miR-4299
ssc-miR-novel-434	4	GGACUCUGAUAGGACUGC	hsa-miR-4522
ssc-miR-novel-435	12	UGGUGGGGUUGUAGGGUC	hsa-miR-92a-2-5p
ssc-miR-novel-436	6	GGAAGGCGGCUCAGGGGG	eca-miR-8987
ssc-miR-novel-437	6	UCUGGUGAUGGGGGUGAG	hsa-miR-4299
ssc-miR-novel-438	9	GCUGGUGUAGUGGUAUCAUGCA	hsa-miR-7978
ssc-miR-novel-439	14	AUGCUAAUCUCUAACCCA	cfa-miR-8825
ssc-miR-novel-440	X	UGCACCUGGGCAAGGAUUC	bta-miR-11974
ssc-miR-novel-441	12	AUGUGGGGAAGGAGAAGG	hsa-miR-6796-5p
ssc-miR-novel-442	X	CCUGAUGACCAUCACAAG	bta-miR-2471-3p
ssc-miR-novel-443	8	AGGUUCUAACCUAGGUCC	hsa-miR-182-3p
ssc-miR-novel-444	1	AGACUUCCUAGAAAAACC	bta-miR-2340
ssc-miR-novel-445	8	GGUGGGAGACUGAGAGGC	bta-miR-2897
ssc-miR-novel-446	15	GAGGAAGGUGGGGAUGAA	hsa-miR-4476
ssc-miR-novel-447	9	CCACCGAAGCCGCGGACC	-
ssc-miR-novel-448	2	CUGCCUGGACCCCUGCUCACC	-
ssc-miR-novel-449	2	CGCGGCGAGAGCGGGCCG	-
ssc-miR-novel-450	3	GUCUGAAGAUGGGGGGGC	-
ssc-miR-novel-451	18	GGAGGAGGAAGGUGUGGAGG	eca-miR-8947
ssc-miR-novel-452	7	CAGAGAAAAGGUCCAGGC	-
ssc-miR-novel-453	11	GGGACUUUGAAAAGAGAG	bta-miR-193a
ssc-miR-novel-454	11	GUCCACGGCCGCCGCAGG	-
ssc-miR-novel-455	6	AGGAAGAGGAUUCUAAGGC	hsa-miR-6077
ssc-miR-novel-456	2	GAGAACUUGGCAGAGAUGC	-
ssc-miR-novel-457	12	GGUUCGAUUCCUGCCGCC	-
ssc-miR-novel-458	2	UCUGAAGGACAGACAUCAC	bta-miR-12041
ssc-miR-novel-459	11	GGUUCGAUCCCUGUCGGG	-
ssc-miR-novel-460	15	AUUGGUUGGAGCUGCUCC	-
ssc-miR-novel-461	17	AGGUGGGCUUAGAAGCAG	bta-miR-541

ssc-miR-novel-462	9	CAUCUGUGGGAUUAUGAC	-
ssc-miR-novel-463	17	AGGCAGCAGUGGGGAGUA	-
ssc-miR-novel-464	5	CCAGGCAGUCAGACUGCA	hsa-miR-4514
ssc-miR-novel-465	1	GGGUGGGGGUUCAAGUCC	hsa-miR-92a-2-5p
ssc-miR-novel-466	17	AACUCAAGAGAGACUGCA	-
ssc-miR-novel-467	11	CAGGCAGGAAACUGGGGC	hsa-miR-940
ssc-miR-novel-468	13	CAGGGGUAGAGGGACAAG	-
ssc-miR-novel-469	14	AGUAAAGCUGGGCACUCUG	-
ssc-miR-novel-470	13	GGCAGUGAGACUGUGGGG	bta-miR-2440
ssc-miR-novel-471	2	GGCCUCAGGGUUGUAAAG	-
ssc-miR-novel-472	2	GUUGGUCCCAAGGGCUGGG	bta-miR-133a
ssc-miR-novel-473	9	GAUGAAGUGGUAAAGGAUG	-
ssc-miR-novel-474	8	GGUGGGGUUGGGGAGCGG	bta-miR-2888
ssc-miR-novel-475	8	CUUGUGGCUGGGAGUGAA	-
ssc-miR-novel-476	8	ACACUGGGACUGAGCCAC	bta-miR-10172-3p
ssc-miR-novel-477	14	UGGGGCUGGAGCAGGUCC	hsa-miR-185-3p
ssc-miR-novel-478	16	UGGAGGUAGAGCCACUGG	-
ssc-miR-novel-479	17	UGGGCUGGUGGUGGUGGGG	eca-miR-8953
ssc-miR-novel-480	9	GGGGAUAUAGCUCAGUGGUAGAGCA	-
ssc-miR-novel-481	8	GCAGGCAAAUCCGCAGCC	hsa-miR-4742-5p
ssc-miR-novel-482	2	CUGGAGUCAGACAGUGGGA	bta-miR-10161-5p
ssc-miR-novel-483	2	CUGGUCUAGUGGCUAGGAUUC	-
ssc-miR-novel-484	6	GGUUAGGAAUCUGCCUAG	hsa-miR-1258
ssc-miR-novel-485	11	ACACUGGGACUGAGCCAC	bta-miR-10172-3p
ssc-miR-novel-486	4	GUGGGAGAGCAGGUCGCC	bta-miR-12060
ssc-miR-novel-487	12	GCUCUGUGAUGAACCCCA	bta-miR-2364
ssc-miR-novel-488	2	CUGUGAAGAAUGGAGCGGCC	hsa-miR-3611
ssc-miR-novel-489	X	AGGUUCUAACCCAGCCCC	hsa-miR-182-3p
ssc-miR-novel-490	5	GACUCGACUGGUGUGCGGACACU	-

ssc-miR-novel-491	6	AGAACUAUCUGGGAAGGUG	-
ssc-miR-novel-492	18	CACUCUAGAGGGACAGCC	hsa-miR-5684
ssc-miR-novel-493	13	GAAACUGCAGGGCUGGAG	hsa-miR-548av-3p
ssc-miR-novel-494	AEMK02000452.1	UGCCCGGGCCUGGGAGCCC	bta-miR-2882
ssc-miR-novel-495	1	GGCAGUGAGACUGUGGGG	bta-miR-2440
ssc-miR-novel-496	5	GAGAAGGGUUCCAUGUGAA	bta-miR-3154
ssc-miR-novel-497	11	CCGGGCAGUUUGACUGGG	hsa-miR-7112-5p
ssc-miR-novel-498	14	UAGGGAGAGGAAAAGAAA	hsa-miR-6760-5p
ssc-miR-novel-499	12	UUGGGAUGGGCGGAGGGGA	bta-miR-12056
ssc-miR-novel-500	9	CACAGGUGGCCAGGUAGA	hsa-miR-552-3p
ssc-miR-novel-501	14	CUAGGUUGGGUGUGGAAGA	bta-miR-2369
ssc-miR-novel-502	X	UAGACAGUUUGACUGGGG	-
ssc-miR-novel-503	2	GUCUGAGUGGGAAAGCAGG	-
ssc-miR-novel-504	5	CGCCUCCUCCUCCCCCCC	hsa-miR-6791-3p
ssc-miR-novel-505	14	GUCAUGCUGGGCACUCUU	-
ssc-miR-novel-506	5	GUCGGGAGGGAAGCGGGG	hsa-miR-6771-5p
ssc-miR-novel-507	14	CCAUGGCCAGAAUGAAGG	-
ssc-miR-novel-508	AEMK02000264.1	UGGGGUGUAGCUCAAUGGUAGAG	bta-miR-2438
ssc-miR-novel-509	3	ACCUGGGUGAUCUGCCCC	hsa-miR-6742-3p
ssc-miR-novel-510	6	GGUGACUGCUCCCUGGGCC	bta-miR-134
ssc-miR-novel-511	1	AGCGGCCGUGUCUGGGGC	cfa-miR-1199-3p
ssc-miR-novel-512	12	GCCUCUGCCUGCCUCCUGGG	cfa-miR-8906
ssc-miR-novel-513	12	CUGCCCUGUCUCCGCCUCCA	bta-miR-874
ssc-miR-novel-514	3	CAGGGCUGGUGGGGCUCUGGGG	hsa-miR-4446-3p
ssc-miR-novel-515	18	CGUGGGGGCCGGGCCGCC	hsa-miR-4447
ssc-miR-novel-516	3	UGAGUGCCAGGUGUUGGG	hsa-miR-1909-5p
ssc-miR-novel-517	14	GGUUGCAGGUUCGAGUCC	eca-miR-9079
ssc-miR-novel-518	13	AGCAUCAGGACGCCCCGU	hsa-miR-4711-5p
ssc-miR-novel-519	2	GAGGUCACGAGUUCAAUUC	bta-miR-1193

ssc-miR-novel-520	6	GCUGGGGUUGAGGCCUGCC	bta-miR-6523a
ssc-miR-novel-521	1	GUAGGACUCGGUGAUGGAC	hsa-miR-3117-3p
ssc-miR-novel-522	17	UUGGGGUUGUAGGACUGA	hsa-miR-3170
ssc-miR-novel-523	5	UGGCAGGGGGGGCUGGGG	hsa-miR-1207-5p
ssc-miR-novel-524	3	AGCUGCACGAGGCCCCGGG	eca-miR-9140
ssc-miR-novel-525	3	CCUGGUGACGAUGGCAGA	hsa-miR-4299
ssc-miR-novel-526	1	UGGCAGUGAGACAGUGGG	bta-miR-34b
ssc-miR-novel-527	1	AAGGAGGGACGCAGGAGG	bta-miR-10180-3p
ssc-miR-novel-528	9	UCCUGAUGGUCCUAGUGC	-
ssc-miR-novel-529	4	GGAAGGGGAGUGAAAGAGA	hsa-miR-3179
ssc-miR-novel-530	13	UGGGGUUGUGGGGAUGCA	bta-miR-2324
ssc-miR-novel-531	13	AGGCAGCAUUGGGGAAUUU	-
ssc-miR-novel-532	7	UGAGAACUCUGCUGAAGG	bta-miR-146b
ssc-miR-novel-533	9	AAUCACUAAUUCCACUGCCAUC	eca-miR-34b-3p
ssc-miR-novel-534	12	CCUGGGUGGGAGGACGUGAAA	eca-miR-8931
ssc-miR-novel-535	15	GCACUCUGUGGAUGCUGC	bta-miR-2461-5p
ssc-miR-novel-536	5	CCCACUGAGCGAGGCCAG	hsa-miR-181a-2-3p
ssc-miR-novel-537	6	CAGGACUGGACCUUGGGCAUCUUC	hsa-miR-3169
ssc-miR-novel-538	1	AGGUUCUAACCCAGGCCC	hsa-miR-182-3p
ssc-miR-novel-539	9	AGAGCUGGGCACUCUGGGG	hsa-miR-4487
ssc-miR-novel-540	1	UUAAGAGGAGGACUUAGG	-
ssc-miR-novel-541	2	GACACAGGACAAUAGGGCAUCUG	hsa-miR-6768-5p
ssc-miR-novel-542	3	GGGCAGGGCUGGGACUUCGGCC	hsa-miR-1207-5p
ssc-miR-novel-543	1	GCUUGGGAAUCUGGCUUU	cfa-miR-8849
ssc-miR-novel-544	6	GAAUGGUCUGGGAAGUCC	eca-miR-7177b
ssc-miR-novel-545	7	GAGAAGAGCGAACGGAGGC	hsa-miR-5197-3p
ssc-miR-novel-546	AEMK02000472.1	CUCCCCGGCCCUGGCUGACC	bta-miR-2889
ssc-miR-novel-547	3	ACCAGGAGGAGCAUGGAGCACU	cfa-miR-665
ssc-miR-novel-548	17	CGGGGCAGCUCAGUACAGGA	eca-miR-486-3p

ssc-miR-novel-549	4	GGCGGGCGCGGGGGUCGGGG	hsa-miR-4767
ssc-miR-novel-550	9	AGUCUAGGCCUGUAGGGCAAUUCC	hsa-miR-8078
ssc-miR-novel-551	12	CAGUGUGCGCAGCCGGGGGA	eca-miR-8986a
ssc-miR-novel-552	12	CUGGGCUCUGGGGGUGGC	hsa-miR-4674
ssc-miR-novel-553	6	CGGGGGAGGACUGGGGAGUCCC	hsa-miR-4723-5p
ssc-miR-novel-554	13	CGGGGGAGGGAGAAGGGG	hsa-miR-4723-5p
ssc-miR-novel-555	7	GGGUGGGCAGGAACCUGC	bta-miR-541
ssc-miR-novel-556	13	GGGAAGGUAGAGCACUGAA	-
ssc-miR-novel-557	1	ACCUGGGUUUGUAGCUCA	hsa-miR-6742-3p
ssc-miR-novel-558	11	CUGCCCAGGACACGCGGAU	eca-miR-8908m
ssc-miR-novel-559	AEMK02000452.1	AUAAUACAUGGUUAACCUCUUU	bta-miR-655
ssc-miR-novel-560	6	GUCGGGGAGGGGAGCGCUGCUCAGC	eca-miR-542-5p
ssc-miR-novel-561	13	UGCCUGUGCUAGAGGUAGAGAGG	hsa-miR-6811-3p
ssc-miR-novel-562	X	GAAGCUUGCUUCCUUUGGGU	hsa-miR-100-3p
ssc-miR-novel-563	5	AUUGGAGGGCAAGUCUGGUACC	-
ssc-miR-novel-564	1	UCUGGUGCUGAUGGCAUC	hsa-miR-548q
ssc-miR-novel-565	1	UAUGGCCACUAUAGAGCA	-
ssc-miR-novel-566	14	GCAGGCACUGGCUCACGGACUC	bta-miR-6715
ssc-miR-novel-567	2	GAAACUGGGGCUGAGGAG	bta-miR-2285aa
ssc-miR-novel-568	3	AGGACAGUGGCCAUGGACC	cfa-miR-8801
ssc-miR-novel-569	8	CGAGGCUGAGGGGCAGGC	bta-miR-485
ssc-miR-novel-570	6	UGGGGACUCUGCCAGGAC	hsa-miR-4652-5p
ssc-miR-novel-571	4	AUGGCCAGACUGAAGCUC	hsa-miR-4435
ssc-miR-novel-572	6	UCUUGGGACCUGUCUCUACACC	cfa-miR-8849
ssc-miR-novel-573	14	GUGCAGGGAGGAGGGGGG	eca-miR-8993
ssc-miR-novel-574	12	GGCGGGGCUGUGCUCUCG	bta-miR-744
ssc-miR-novel-575	5	UAAAGUUAUAUAAGGGUUUU	bta-miR-2285ct
ssc-miR-novel-576	3	ACUCUGGUGUAGGCCCGCA	hsa-miR-6764-3p
ssc-miR-novel-577	AEMK02000264.1	GGGGGUGUAGCUCAGUGGUAGAGC	bta-miR-2438

ssc-miR-novel-578	AEMK02000682.1	CUCUGGUGGAGGCCUGCA	-
ssc-miR-novel-579	X	GCCAGAGAGGGUGAGAGC	-
ssc-miR-novel-580	14	AAAAGGACUUUGAAAAGA	-
ssc-miR-novel-581	11	CUGACUCACUCUGUUGUGCAGC	-
ssc-miR-novel-582	9	GUCAGAAUCCCACCCAGGC	-
ssc-miR-novel-583	9	CAUGGAGGUCUCUGUCUGGC	hsa-miR-4802-5p
ssc-miR-novel-584	6	UCGGGGAGGCUGUGCAGCGCG	hsa-miR-6777-5p
ssc-miR-novel-585	AEMK02000452.1	AAUGUUGCUCGGUGAACCCC	cfa-miR-409
ssc-miR-novel-586	7	GGCGGCGGCGGCGGCGGC	bta-miR-11975
ssc-miR-novel-587	3	CGGGGCUGGGCGCGCGCC	hsa-miR-185-3p
ssc-miR-novel-588	X	ACUGGUUGGAUCCUGCUU	-
ssc-miR-novel-589	7	CCGCCUCCUCUCGCCGCC	bta-miR-1281
ssc-miR-novel-590	3	CCGCACUGUGGGUACUUGC	bta-miR-2456
ssc-miR-novel-591	8	GGCGGGCGCGGCGGCCGC	hsa-miR-4767
ssc-miR-novel-592	4	GCUGAUUAUCGAGGCGAUUCUGAUC	hsa-miR-3920
ssc-miR-novel-593	7	ACCAGCCUGGACUGAGGUG	hsa-miR-6849-3p
ssc-miR-novel-594	13	AAGGAGCUGAGGACGGAGAAG	bta-miR-28
ssc-miR-novel-595	6	GGGCCUGUGGCUCAGAGG	hsa-miR-6735-3p
ssc-miR-novel-596	2	AAGGGUCUGGAGUUUGGG	hsa-miR-1182
ssc-miR-novel-597	12	GAGGGAGAGAACACCAUCUGAGUGG	hsa-miR-6760-5p
ssc-miR-novel-598	14	CAGUGGAUAGAGCUCUGUG	hsa-miR-3681-5p
ssc-miR-novel-599	18	AUCCCACUCUGGUCACCA	hsa-miR-3156-3p
ssc-miR-novel-600	12	CCUUCGGUGAGGGCUGCCGUGA	-
ssc-miR-novel-601	1	CACCCCAGACCCAAUGGCAAGA	-
ssc-miR-novel-602	10	ACUCUCUCUUCCUCUGUCA	hsa-miR-7110-3p
ssc-miR-novel-603	11	CGCGGAGGGCGGGGCCUGGCG	bta-miR-658
ssc-miR-novel-604	1	UCUGGGCUUCCUGGCAGCUGC	oar-miR-134-3p
ssc-miR-novel-605	14	GGAGGGAGACUGAGAAGC	hsa-miR-4510
ssc-miR-novel-606	3	UUGGCGGCCAUGGCGGUCUCGGA	eca-miR-7045

ssc-miR-novel-607	X	UGAACGGCGCCUGUGUGGUUAGA	-
ssc-miR-novel-608	14	UUCUCCAAGGACACUCUGUC	hsa-miR-515-5p
ssc-miR-novel-609	2	GUUGGAUUUAUGAAAGAC	hsa-miR-5004-3p
ssc-miR-novel-610	6	UCUGACCCUGGCCCCUGA	cfa-miR-8853
ssc-miR-novel-611	1	GUGGGAGGAGCCCUGGGC	bta-miR-30b-3p
ssc-miR-novel-612	8	ACGCGCUGCCUUUGAGCCCCCG	-
ssc-miR-novel-613	4	GUGAGUUUGUAGGGUUGGAGA	-
ssc-miR-novel-614	5	GCCGGCGGCGGCGGCGAC	-
ssc-miR-novel-615	4	UCUGGGAAGAGGCAGGGC	bta-miR-2447
ssc-miR-novel-616	5	CUCUCCACUUUCACCUUCA	eca-miR-9135
ssc-miR-novel-617	7	CAAAACAGAGAAUCAAGACUGA	-
ssc-miR-novel-618	3	GGCGCGCCCCCGGCCGCGG	hsa-miR-4783-5p
ssc-miR-novel-619	17	UGCUCCCGCCGCUGCUGCUGC	bta-miR-2388-5p
ssc-miR-novel-620	AEMK02000452.1	UCCCCCGGCCGCGCGCGC	hsa-miR-6840-5p
ssc-miR-novel-621	3	GGCGGCGGCGGCGGCGGC	bta-miR-11975
ssc-miR-novel-622	11	UCUGACCAGUAUCCAUGAGG	-
ssc-miR-novel-623	10	AGGACAGGGCUCCUGGCUC	bta-miR-10162-5p
ssc-miR-novel-624	6	GGGGGUCCGCCGGCCUUGGG	bta-miR-615
ssc-miR-novel-625	3	CUGGCAUCGUGAUGGACUCC	hsa-miR-4642
ssc-miR-novel-626	4	GGCGGGCGCGGCGGCCGC	hsa-miR-4767
ssc-miR-novel-627	14	CCAAACCAGUUGUGCCUGUAGA	hsa-miR-6715a-3p
ssc-miR-novel-628	5	CCCGGGGCGCGAGCCCGG	bta-miR-12030
ssc-miR-novel-629	13	GCUGGCCGAGGCGCCGCC	-
ssc-miR-novel-630	8	GCCCCCUCCGGCCCGGAC	bta-miR-2328-5p
ssc-miR-novel-631	6	AAUUCCACCACGUUCACG	-
ssc-miR-novel-632	3	CGCCCGCCGCGCCGGGGAGA	hsa-miR-4707-3p
ssc-miR-novel-633	2	UCUGGACAUGAAGCCCCUCUC	hsa-miR-4638-3p
ssc-miR-novel-634	2	UGGGGCCGUGGGGCCAGG	eca-miR-9056
ssc-miR-novel-635	4	GGUCACUCGGCCGAGGGC	hsa-miR-668-3p

ssc-miR-novel-636	10	CCUGCACCCGCCGCCCGCCC	eca-miR-9143
ssc-miR-novel-637	2	UGUGUCUGUGACAUCUGCUGGCA	-
ssc-miR-novel-638	13	CAGGACUGGGCUCGGGGG	hsa-miR-3169
ssc-miR-novel-639	3	GCUGGCAGGGCCCCCACC	eca-miR-9171
ssc-miR-novel-640	4	CGGCUCUGGGUCUGUGGGGAG	bta-miR-760-3p
ssc-miR-novel-641	15	GGUGGCCCAAGCCCGGGG	hsa-miR-663b
ssc-miR-novel-642	2	GGGCCUCUUCCCCGGGCA	hsa-miR-4757-5p
ssc-miR-novel-643	X	GGGGGCCGGCGGCGGCGGC	eca-miR-9056
ssc-miR-novel-644	15	GGUUCAAUCCCGGCCGGGCA	-
ssc-miR-novel-645	1	AGAGCCCGGGUGGCUGGCGGC	bta-miR-2373-3p
ssc-miR-novel-646	14	AUGGGUGACCUCCUGGGG	hsa-miR-1262
ssc-miR-novel-647	6	AGAGCUGGUCUUGAACGUC	hsa-miR-4487
ssc-miR-novel-648	11	GGGGCCGGGGGUGGGGCC	hsa-miR-6781-5p
ssc-miR-novel-649	18	ACUUGGGCCUGGCUGGCCG	cfa-miR-8903
ssc-miR-novel-650	AEMK02000452.1	AAUAUAACACAGAUGGCCUG	bta-miR-410
ssc-miR-novel-651	7	CCUCUGAGUCCCUACCACUGCCUC	bta-miR-2376
ssc-miR-novel-652	3	CGCCCUGCCCAGGGUGCC	bta-miR-12049
ssc-miR-novel-653	12	GGCAAGUCUUUUCAUGAUCUGAC	-
ssc-miR-novel-654	9	GGUUCGUUUCCCGGCCCA	-
ssc-miR-novel-655	6	AGGGGCAGAGGGAAUUUCC	eca-miR-486-3p
ssc-miR-novel-656	15	CGGGCUGUGGAGGGAGGG	hsa-miR-4675
ssc-miR-novel-657	2	GGUGGGGGCCGGGCCGCC	hsa-miR-4447
ssc-miR-novel-658	13	GGGCUGCUUGUCCGUGCUCUUCC	bta-miR-2379
ssc-miR-novel-659	3	AUUCCCUUCUGGGCCUCAGGA	hsa-miR-4755-5p
ssc-miR-novel-660	13	UUGGGACUGAGACACGGCCA	bta-miR-2287
ssc-miR-novel-661	15	CUCUCUCUGGGCCACCCC	hsa-miR-6873-3p
ssc-miR-novel-662	2	GGUGGGAGGUGAGUGCCA	bta-miR-2897
ssc-miR-novel-663	15	CAGGAGUGUAAUUAUUUCAUGA	hsa-miR-4433b-3p
ssc-miR-novel-664	3	AGGAGACGUGUGCUGGGCCC	cfa-miR-139

ssc-miR-novel-665	AEMK02000289.1	CCCGGUUCCGAGUUCCUGUGGGGUC	eca-miR-671-3p
ssc-miR-novel-666	15	CCCCUCUGGGGGCUGGGC	hsa-miR-2276-5p
ssc-miR-novel-667	1	AGCUUAUUAUCUGAUGCC	-
ssc-miR-novel-668	13	UUUGUUCUCCGUGCGGGCAGA	bta-miR-7857-3p
ssc-miR-novel-669	3	GUGGCUGUGGCUCGACCCC	bta-miR-2902
ssc-miR-novel-670	10	GUGGCUGUGGCAUAGGCC	bta-miR-2902
ssc-miR-novel-671	6	GCCCCCAGGGUCUCGGGCU	eca-miR-361-3p
ssc-miR-novel-672	6	ACCCUGACUUCUGGAAGG	hsa-miR-4732-3p
ssc-miR-novel-673	6	CUGGGCCCGGGGAGAGGAGCC	hsa-miR-6724-5p
ssc-miR-novel-674	14	CCUGACCCUGACCUUUCCCA	cfa-miR-8853
ssc-miR-novel-675	3	GGAGCUGGCUGGGAGCUC	hsa-miR-4487
ssc-miR-novel-676	7	AGGUCAAGGAAGUUGGUGCC	eca-miR-1898
ssc-miR-novel-677	5	UGCUGGUGGCCCUGGCAGGG	bta-miR-138
ssc-miR-novel-678	1	CGGGCGGGAGCCCGCGGGG	bta-miR-2881
ssc-miR-novel-679	2	AAGGAUUGGCUCUAAGGA	-
ssc-miR-novel-680	5	UAUCAAGUAUUGGUGAGGAUC	-
ssc-miR-novel-681	16	UCCCUGUGGUCUAGUGGC	hsa-miR-146b-3p
ssc-miR-novel-682	AEMK02000452.1	CUGGCUGGGCUGGUUUGCGC	hsa-miR-6842-3p
ssc-miR-novel-683	4	GCUGGCAGGGCCCCCACC	eca-miR-9171
ssc-miR-novel-684	2	UAUGUCUGCGAUUGGCAGAAG	bta-miR-654
ssc-miR-novel-685	14	AGGACUGGCUCUGAGGGC	hsa-miR-4515
ssc-miR-novel-686	1	CAUUGGCUGGAAAUGGACA	-
ssc-miR-novel-687	7	GACUGGACCACCAAUGGC	hsa-miR-3654
ssc-miR-novel-688	7	GUGCCUCCCGGCCCCUCC	hsa-miR-6836-3p
ssc-miR-novel-689	5	UGACCAUGUGGACCCAGGGAG	hsa-miR-591
ssc-miR-novel-690	1	AGGCAAGGAGGCCUCUGAGGC	hsa-miR-6809-5p
ssc-miR-novel-691	13	GGCCGGGCCCGCCCCCGC	cfa-miR-8834a
ssc-miR-novel-692	5	CGGGGAGCUCCCUCGGGUCC	bta-miR-1343-5p
ssc-miR-novel-693	4	CGAAACUGGGGCUGGGCAUC	hsa-miR-4738-3p

ssc-miR-novel-694	16	GCCUCUCUCUCCCCACCA	hsa-miR-3183
ssc-miR-novel-695	1	GCCCUUGUAGCUCAGUGGG	hsa-miR-6780b-3p
ssc-miR-novel-696	15	AUGACUGGGUCACUUUGCUG	-
ssc-miR-novel-697	X	GCCCCCUCCCAGCGGUUGUCACG	bta-miR-2328-5p
ssc-miR-novel-698	6	GGGGCCGGCCGUGUCCUGC	hsa-miR-6781-5p
ssc-miR-novel-699	7	UCGAGGACUGAUCUCUUGU	hsa-miR-611
ssc-miR-novel-700	12	CUGGGCUGUGUGAAGGGGC	hsa-miR-3620-5p
ssc-miR-novel-701	15	GGUAGAGUAUUUGACUGC	-
ssc-miR-novel-702	4	AGAGGGAGAGAACACGGA	hsa-miR-4510
ssc-miR-novel-703	6	CGGGGCCGGCUGUGGCUC	eca-miR-9056
ssc-miR-novel-704	6	UCCUCUGGAGGCUCGAGAAGA	bta-miR-2290
ssc-miR-novel-705	AEMK02000452.1	AGAUAUUGCACGGUUGAUCUC	bta-miR-154c
ssc-miR-novel-706	2	UCUGGGGGUGGAGCGGCC	hsa-miR-637
ssc-miR-novel-707	6	GCGGGGGUGGCGGCGGGC	hsa-miR-4787-5p
ssc-miR-novel-708	14	GGGCUGGGCUGUGAUGGG	eca-miR-8943
ssc-miR-novel-709	12	CCUGCAGGGCUCGGAACCUC	bta-miR-10179-5p
ssc-miR-novel-710	15	AGGAAGGUGAGGAUGACU	-
ssc-miR-novel-711	2	GCCUAGUGGUUAGGACAU	-
ssc-miR-novel-712	1	AGGCAAGGGCAGGUGGAGU	hsa-miR-6809-5p
ssc-miR-novel-713	2	AGGGGCUGGGGUACUGAGG	hsa-miR-185-3p
ssc-miR-novel-714	12	UAGGGACCCUGUGGGCAGC	bta-miR-2285ca
ssc-miR-novel-715	3	AGUCUGGGGACUGGGGGC	bta-miR-12028
ssc-miR-novel-716	7	GCUGGUUUAGACUCUGGACACCC	hsa-miR-29b-1-5p
ssc-miR-novel-717	8	GAGCGACCGCGGUGGGCCG	hsa-miR-4670-5p
ssc-miR-novel-718	7	AGACUCUGGGCUGGGCAUCUC	hsa-miR-4522
ssc-miR-novel-719	12	UAUCCCCAUGGAGUCUGUUGC	-
ssc-miR-novel-720	15	CGGGCCUGGCUCAUCCCA	hsa-miR-2861
ssc-miR-novel-721	AEMK02000452.1	CCUGGGAGUUCUGUGGCUUG	bta-miR-12057
ssc-miR-novel-722	2	GUCACCACUGAGGAGGGAGCC	bta-miR-197

ssc-miR-novel-723	3	AUGGGUGACCUCCUGGGU	hsa-miR-1262
ssc-miR-novel-724	17	CUUGGGGUUGUAGGACUGA	cfa-miR-8899
ssc-miR-novel-725	3	UCCAGGGCUGCCCUUCUUAC	hsa-miR-4685-5p
ssc-miR-novel-726	AEMK02000452.1	AAUAUUAUACAGUCAACCUC	bta-miR-656
ssc-miR-novel-727	12	GGGCAGCGCUGAGGGCUGGGGG	eca-miR-885-3p
ssc-miR-novel-728	X	CAUGCCUUGAGUGUAGGAC	bta-miR-532
ssc-miR-novel-729	1	CAGCCAGGGGAUAGGGACAGG	cfa-miR-8844
ssc-miR-novel-730	14	CUGGGGAAAGGGAGUCCC	bta-miR-6525
ssc-miR-novel-731	2	CCUGGGGGCUGUAAAUCC	hsa-miR-637
ssc-miR-novel-732	12	CCUGACCCAGGCCCCUGC	cfa-miR-8853
ssc-miR-novel-733	2	CGGAAGCCCGGAGCCCUCC	bta-miR-671
ssc-miR-novel-734	7	CCUGGACUGGUUCGAGAGGCC	bta-miR-378
ssc-miR-novel-735	2	CGGAGCAGAAUCCCUGGGCC	hsa-miR-6081
ssc-miR-novel-736	AEMK02000361.1	CUGACAUCUGUUUUUGCC	bta-miR-489
ssc-miR-novel-737	X	CCUCCUCCCGGCCCCGGC	bta-miR-12059
ssc-miR-novel-738	13	CUCGGAGAGACACCCAGCUUGC	bta-miR-3957
ssc-miR-novel-739	2	UUGAGAUGAGAGCCCUUGGCCAC	-
ssc-miR-novel-740	14	GUGUGGGCAGUGGGCAGGGCC	hsa-miR-4296
ssc-miR-novel-741	7	GGAGGGAGACUGAGAAGC	hsa-miR-4510
ssc-miR-novel-742	AEMK02000598.1	AAGAUUGUUCUGAGGUUG	-
ssc-miR-novel-743	10	CAGUUGGUUAGAGCUUGG	-
ssc-miR-novel-744	9	GACUGGCCGGGUCGUGCC	bta-miR-193a-3p
ssc-miR-novel-745	7	GGGGGCAGUUCUUGGGAGC	eca-miR-486-3p
ssc-miR-novel-746	3	UGCUGGUGAAUUCUUGAGAGC	bta-miR-138
ssc-miR-novel-747	2	UUCACAGUUCAUAGGAGGA	bta-miR-27a-3p
ssc-miR-novel-748	3	AGCCAGUGGCGGGGCUGUC	eca-miR-9107
ssc-miR-novel-749	9	AAGGCCUGGGAACUCUGGGC	bta-miR-2306
ssc-miR-novel-750	1	AACGCUGGGGUUAUAGGAU	-
ssc-miR-novel-751	12	UUGCAGCUGUGUCUGAGGGC	bta-miR-1301

ssc-miR-novel-752	13	ACAGAAAGAGAUGGGGGC	bta-miR-2285z
ssc-miR-novel-753	7	GGGCGGGGGUCCGCCGGCC	hsa-miR-663a
ssc-miR-novel-754	17	GAGUUAGAAUCAGGGGGGC	eca-miR-9016
ssc-miR-novel-755	6	GAGGGCUGCUUUGCUGCU	hsa-miR-4446-3p
ssc-miR-novel-756	9	CCGUGUCCCCUUCCCACC	hsa-miR-6774-3p
ssc-miR-novel-757	2	UUGAGUGAAUGAGAUCAGCC	bta-miR-2383
ssc-miR-novel-758	3	GGAGGUGGCCUGUGGGCA	hsa-miR-6134
ssc-miR-novel-759	4	GAGCAAGGAUUGACAGAC	eca-miR-8962
ssc-miR-novel-760	3	AGGACUGGAGUUCUUUGUU	hsa-miR-4515
ssc-miR-novel-761	AEMK02000449.1	UUUCCUCACAUCAUGGAC	hsa-miR-670-3p
ssc-miR-novel-762	6	ACCUGUGCUGGAUUGCCA	bta-miR-6526
ssc-miR-novel-763	15	UCUGCCAUCUUCUCUGUG	hsa-miR-4769-3p
ssc-miR-novel-764	13	GCGGGCGGCUGGUGCACC	-
ssc-miR-novel-765	12	ACCAAGAGAGCAUUGGCCUUGA	eca-miR-9160
ssc-miR-novel-766	14	CGGCGGCGCGGGCCCCGGG	hsa-miR-4467
ssc-miR-novel-767	1	UAGCAGCUGUUAUUGGGUGAAC	hsa-miR-646
ssc-miR-novel-768	4	AAUCUGAGGAUCUGGCCUAGA	hsa-miR-3200-5p
ssc-miR-novel-769	6	CCCAGCGCUCCCCAGAUGCCUC	hsa-miR-4738-5p
ssc-miR-novel-770	2	AGCACUUACGGAUUCUGGGGGU	-
ssc-miR-novel-771	6	AUUUGCAUGUUCUAGAAAUUAGGA	eca-miR-8981
ssc-miR-novel-772	7	UGCUGGUGGCCCUGGCAGGG	bta-miR-138
ssc-miR-novel-773	AEMK02000452.1	AACACACCUGGUUAACCUCU	bta-miR-329a
ssc-miR-novel-774	AEMK02000452.1	UGAAAUGUUUAGGACCACUAG	-
ssc-miR-novel-775	6	GUGGCCAGGGUGAAGCGCC	hsa-miR-4435
ssc-miR-novel-776	18	GAUGAUGCGGGUUCGAUUCC	hsa-miR-1322
ssc-miR-novel-777	1	GGGAGAAGGGGUGCUGGC	hsa-miR-4434
ssc-miR-novel-778	9	CACAGCAAGUGUAGACAGGCA	bta-miR-3120
ssc-miR-novel-779	15	UCCCUGGGGUUGUAGGAC	hsa-miR-4292
ssc-miR-novel-780	7	GGAAGAAGAUGACAGUACC	hsa-miR-3185

ssc-miR-novel-781	3	CUGGCCUUGCUCAUUGGGUUGAGGA	-
ssc-miR-novel-782	8	CCAGAGAUGGGGGAGGGC	hsa-miR-4756-3p
ssc-miR-novel-783	1	ACAGCAGGUGAAAAUUCC	bta-miR-214
ssc-miR-novel-784	1	AGGCCCUUACAUAGUCAGAC	hsa-miR-6851-3p
ssc-miR-novel-785	AEMK02000489.1	GUGAGGCCCUCGGAUCGGC	-
ssc-miR-novel-786	AEMK02000695.1	GUGAGGCCCUCGGAUCGGC	-
ssc-miR-novel-787	AEMK02000542.1	CCGUGAAGUCGACCUGUGGUUC	-
ssc-miR-novel-788	18	GUUCUCACCUCGCUGGUGUC	-
ssc-miR-novel-789	5	AUGAACGCCAAGCUGGCCC	-
ssc-miR-novel-790	2	CAGCACUCUGUCCCCCCCAG	-
ssc-miR-novel-791	4	CCGAGCCUGGCCUCGGGC	bta-miR-10225a
ssc-miR-novel-792	13	AGGAGGAGGAGGAGGACG	bta-miR-10176-5p
ssc-miR-novel-793	5	CAGAAGCAACAGGAAGCUG	-
ssc-miR-novel-794	1	UCUGUGCCGAAAGCAUGGGGA	eca-miR-6529
ssc-miR-novel-795	7	GGUUCAAUCCCCAGCACC	-
ssc-miR-novel-796	AEMK02000452.1	ACCCGUCCCGUGCGUCCCCGGA	bta-miR-1247-5p
ssc-miR-novel-797	12	ACUGCCCCAGGUGCUGCUGG	hsa-miR-1913
ssc-miR-novel-798	Y	CAGUGGGUUAAGGAUCCGGC	-
ssc-miR-novel-799	6	CUCGGAGCAGCGCGCGAGA	-
ssc-miR-novel-800	13	AGGUCCCGGGUUCCUCCCG	hsa-miR-4281
ssc-miR-novel-801	2	GGGGUGGGGGCCGGGCCGCC	hsa-miR-6756-5p
ssc-miR-novel-802	2	GCCCAUGUGCAGGGUGAAGG	-
ssc-miR-novel-803	7	CAUAGUUUGGUAUUGGUG	bta-miR-2371
ssc-miR-novel-804	18	GUGUGGAUGUCUGAAGGAGGG	hsa-miR-4752
ssc-miR-novel-805	5	GGUUUGAUUCCCGGCUGG	-
ssc-miR-novel-806	2	CCUCUGCGCCCUCCUGCCCAGC	bta-miR-6529b
ssc-miR-novel-807	15	GGGGUGGAGCAGUUGGCAGA	bta-miR-10171-3p
ssc-miR-novel-808	7	GUGGGACUUGGAGAAAAACUUGC	bta-miR-2287
ssc-miR-novel-809	6	CUUCAGGUCCCUGUUCAGGC	-

ssc-miR-novel-810	5	UACUCCUGAAGGCUGAAA	-
ssc-miR-novel-811	5	GGACUCGACUGGUGUGCGGACAC	-
ssc-miR-novel-812	17	UUCUCAGGUUUGACAGUCC	bta-miR-1388-3p
ssc-miR-novel-813	2	UCUCUGCUCACCACCACAGCC	hsa-miR-6728-3p
ssc-miR-novel-814	14	AGGUUGGGUGUGGAAGAG	hsa-miR-92a-1-5p
ssc-miR-novel-815	6	CAGGGAGGAGAGGCUGGAG	bta-miR-149-3p
ssc-miR-novel-816	AEMK02000639.1	CCCCGGGGUCGACCUGCUG	bta-miR-12034
ssc-miR-novel-817	X	AAAUUUGAGAGGCUCUGC	-
ssc-miR-novel-818	2	UUGGUCUGUUAAGGACUCUGCUC	bta-miR-2357
ssc-miR-novel-819	14	GAGGGCUGCUCUGUUGGGG	hsa-miR-4446-3p
ssc-miR-novel-820	7	GGGGGGUGGCUCGGGGGG	bta-miR-2305
ssc-miR-novel-821	17	AGGAAUGAGACAGAGAUG	-
ssc-miR-novel-822	10	CUUGGGAGCCAACCUGUGACUG	hsa-miR-887-5p
ssc-miR-novel-823	5	GUCUGGGAGGAAGGCGCC	hsa-miR-4314
ssc-miR-novel-824	15	CAGUGGUAGAGCACUCCC	-
ssc-miR-novel-825	2	GGUUCUGGGUUCGGGUCC	-
ssc-miR-novel-826	5	GAGACUGAGGUGCUGGAG	hsa-miR-4499
ssc-miR-novel-827	1	CACCGGGGCUGAGGACAG	hsa-miR-4655-5p
ssc-miR-novel-828	15	CGAGGAGGAGGCAGGGGC	eca-miR-8947
ssc-miR-novel-829	14	CAGGGAGGAGAGGCUGGAG	bta-miR-149-3p
ssc-miR-novel-830	1	CCCUUCCCAGGUCGGGGGA	hsa-miR-6734-3p
ssc-miR-novel-831	18	GUGAGGAACCCUUGGCCC	hsa-miR-3915
ssc-miR-novel-832	AEMK02000479.1	AUGGGGGUCAUGGGGAAGGAG	bta-miR-2285ah-5p
ssc-miR-novel-833	13	CCCUGGGGUCAGACUUAGAAAGGGG	eca-miR-8970
ssc-miR-novel-834	13	GGAGAUGAAAGAAUGGGA	bta-miR-143
ssc-miR-novel-835	11	UGUGUGGCUAAGUGGUAGA	hsa-miR-644a
ssc-miR-novel-836	1	AGAAGAACUCAGAGAUGGG	eca-miR-8910
ssc-miR-novel-837	AEMK02000452.1	CUGCUCACCAGCUUUCUCUCC	hsa-miR-1178-3p
ssc-miR-novel-838	X	AUAGCAGCGCCUCACGUUUUGA	hsa-miR-4524a-5p

ssc-miR-novel-839	7	CAGGGCUUAGGAGCUUUUGGCG	hsa-miR-3158-3p
ssc-miR-novel-840	11	CGGUCCCCCGCCGUCCCCGCCG	-
ssc-miR-novel-841	AEMK02000510.1	GGUGGGAGAUUGUGAAGU	bta-miR-2897
ssc-miR-novel-842	16	CUAACUCCCGUGUACUGCACU	hsa-miR-4764-3p
ssc-miR-novel-843	3	AGGUGAGGGGAAGCAGCGGC	hsa-miR-4710
ssc-miR-novel-844	2	CAGGCUGAAUACUCGCCC	hsa-miR-3929
ssc-miR-novel-845	8	GUCUGGCUCUUUGCGGGA	eca-miR-8984
ssc-miR-novel-846	4	UUCCUGGGGUUGUAGGAC	bta-miR-1343-3p
ssc-miR-novel-847	1	UUCAUUAGCUAGUUGGUA	-
ssc-miR-novel-848	AEMK02000682.1	UGGUGGACUGGCCAAUCC	hsa-miR-8071
ssc-miR-novel-849	AEMK02000452.1	AUCACACAAAGGCAACUUUUG	bta-miR-377
ssc-miR-novel-850	18	GUGUGGCCUCUGUCUUCA	cfa-miR-7180
ssc-miR-novel-851	X	GGAGGUAACUAAAAGAUC	bta-miR-6119-5p
ssc-miR-novel-852	X	UGCUCCUCUUACUUAGUAGCC	hsa-miR-4268
ssc-miR-novel-853	14	GUGGGCUGUAACUCAUCC	hsa-miR-3620-5p
ssc-miR-novel-854	11	GAAACUGAAAAACUUGAG	-
ssc-miR-novel-855	9	AGAGUCAAAUGCUUCUAAU	-
ssc-miR-novel-856	13	AAGAAGGGCUCAGAGAUGA	bta-miR-3154
ssc-miR-novel-857	17	GGAAGAGAGCUGGAAAGCU	-
ssc-miR-novel-858	8	GCAGGAACUCUUAGGUGGGGG	bta-miR-873
ssc-miR-novel-859	13	UUGGUGAGACUGUAGAAAAA	hsa-miR-7855-5p
ssc-miR-novel-860	2	CUGGUGGCUGGCUUUACCUC	eca-miR-8989
ssc-miR-novel-861	6	CAGAGGUUACAGGUAUGGAG	hsa-miR-12127
ssc-miR-novel-862	9	UUGGGGGGAACGAGGGGAAG	hsa-miR-7109-5p
ssc-miR-novel-863	8	GUGGGGAGUUUGACUGGGG	hsa-miR-3150a-3p
ssc-miR-novel-864	1	GGAAGGAAGUGGAGGCAC	hsa-miR-1252-5p
ssc-miR-novel-865	3	GCAGAUGAAGGUUGAACC	hsa-miR-1245b-3p
ssc-miR-novel-866	9	AUGGAGGUAGAGCUCUGA	bta-miR-12054
ssc-miR-novel-867	17	UAGCCUGGGAACCUCCAUAUGCC	cfa-miR-8800

ssc-miR-novel-868	9	GCUGGUGUAGUGGUAUCAUGC	hsa-miR-7978
ssc-miR-novel-869	1	UCUGAAGGCCUGUCAUCC	bta-miR-12041
ssc-miR-novel-870	5	UGGUGCGGGUUCGAUUCC	hsa-miR-675-5p
ssc-miR-novel-871	11	UGCACAACAGAGCGAGCCAGCUG	-
ssc-miR-novel-872	4	GCAGGAGAUGGACAACAG	hsa-miR-4768-3p
ssc-miR-novel-873	1	CACCCCAGACCCAAUGGCAAG	-
ssc-miR-novel-874	12	GCUCUGUGAUGAACCCCAUGCG	bta-miR-2364
ssc-miR-novel-875	2	GUUUAGCUCAGCUGGGGG	-
ssc-miR-novel-876	13	GAGGUGUGAUGGGGAAGG	eca-miR-9017
ssc-miR-novel-877	6	AGCCGAGUGGGAAAGGAUG	-
ssc-miR-novel-878	14	CCGUGGACCACGUUCCCC	-
ssc-miR-novel-879	7	GGAGAAGCUGGGCACUCUG	bta-miR-2486-5p
ssc-miR-novel-880	9	GGCAGGAGGCCGUCUAUC	hsa-miR-4722-5p
ssc-miR-novel-881	8	GAGCUGAAGCUGCGGAUU	-
ssc-miR-novel-882	9	CAGGUGGCUACAGCGGCG	hsa-miR-6815-5p
ssc-miR-novel-883	8	UCUGGUUAUGAUGUGUUCU	-
ssc-miR-novel-884	7	GGCUGGAGACUGAGAAGC	eca-miR-8929
ssc-miR-novel-885	15	AAGAGUGGCAGGUUUCUC	-
ssc-miR-novel-886	7	UUCUGAGAGGAAGGCCCC	bta-miR-11996
ssc-miR-novel-887	2	AGGGUGAAGGAACUAGGC	-
ssc-miR-novel-888	14	GAGAGAAGAAAGGGAGGC	-
ssc-miR-novel-889	16	UAGGCUGGGUGUGGAAGC	bta-miR-940
ssc-miR-novel-890	18	GGCGGGAGACUGGGAAAC	-
ssc-miR-novel-891	12	CGCUUCCAGGCAAAGCCAC	hsa-miR-2115-5p
ssc-miR-novel-892	3	GAGCUUCACUGUAGCUCA	-
ssc-miR-novel-893	13	GGUUGCAGGUUCGGGUCC	eca-miR-9079
ssc-miR-novel-894	3	CUGUCUGAAGAUGGGGGGGC	-
ssc-miR-novel-895	2	UAAGCAGCAAGGUUGUCUGUC	bta-miR-562
ssc-miR-novel-896	12	GUUUUUAGGACUCUGAGC	-

ssc-miR-novel-897	13	AUGGAGCUGUGAUGGGA	eca-miR-8944
ssc-miR-novel-898	6	GGGGGUUGUGGGAGUCCG	bta-miR-2324
ssc-miR-novel-899	8	CUGCAGAGCACUGGCACA	hsa-miR-3678-3p
ssc-miR-novel-900	18	GCUGGAGGCCGCCUAUCC	cfa-miR-1843
ssc-miR-novel-901	4	UGGUGGAGUGUGGGACCU	bta-miR-2898
ssc-miR-novel-902	16	UCACGCCUGCUGUGAAAGC	eca-miR-9037
ssc-miR-novel-903	2	GGGGGUGGGCGGGCGGGGCG	hsa-miR-608
ssc-miR-novel-904	3	GGUUCAAAUCCAGCCUCC	-
ssc-miR-novel-905	7	GCCUGAGACACGGCCCAG	hsa-miR-4324
ssc-miR-novel-906	2	CCUGCAGGAGGUGAGGGC	bta-miR-10179-5p
ssc-miR-novel-907	7	UCUGAAAGUACUGGGGGCCUC	hsa-miR-4766-5p
ssc-miR-novel-908	15	CAGGAGUGGGACUAUGGG	hsa-miR-4433b-3p
ssc-miR-novel-909	11	AGAAGAAGUCAGAGAUGA	hsa-miR-3185
ssc-miR-novel-910	7	GAGGACUGCAGUGACUGCGUGC	hsa-miR-3169
ssc-miR-novel-911	16	GCACUGUUGUUAAGGAAC	hsa-miR-1208
ssc-miR-novel-912	4	AGGGCUGGGCACAGGGGG	eca-miR-8953
ssc-miR-novel-913	4	UCGGCCGCGCCCCACUUGUC	hsa-miR-1908-3p
ssc-miR-novel-914	13	GUGAGGUGGCCGAGCAGG	bta-miR-2451
ssc-miR-novel-915	3	UGCGGCCGGCUGGCGGGGCA	cfa-miR-1199-3p
ssc-miR-novel-916	1	GAAGGAUGUAGGUAGGCA	-
ssc-miR-novel-917	5	GUUAAUAACUAGUAUCCA	-
ssc-miR-novel-918	14	GACACUGAGGCUCAAAAGC	-
ssc-miR-novel-919	3	CUGGCUCUGCUCUGAGCU	bta-miR-12004
ssc-miR-novel-920	12	AGCCGCUGAGAGAUGGAC	eca-miR-8999
ssc-miR-novel-921	3	GCAUCCUCGGGGCUCUGUUCCUC	hsa-miR-5196-3p
ssc-miR-novel-922	5	AUCAAGUAUUGGUGAGGA	bta-miR-26a
ssc-miR-novel-923	6	GCCUGGGGGCUGAGGGCG	eca-miR-8970
ssc-miR-novel-924	4	CGGGGCGGGCGGGUCGCGGG	bta-miR-1777b
ssc-miR-novel-925	7	CUGGCUGCCUGCCGGGGC	hsa-miR-647

ssc-miR-novel-926	14	GUCGGGAGGUCCUGGCUGC	hsa-miR-6771-5p
ssc-miR-novel-927	13	UUAAAUGAAAAGAGGAGUC	-
ssc-miR-novel-928	15	AUCAUGGGGGUUGUUGGAG	eca-miR-9179
ssc-miR-novel-929	1	CAUGUGGACAGGAGCUUGGACUUC	bta-miR-4657
ssc-miR-novel-930	5	GAGAAGGGUUCCAUGUGA	bta-miR-3154
ssc-miR-novel-931	15	CUAGGAGGAUGGCCCUGCUGC	hsa-miR-4266
ssc-miR-novel-932	13	UGGGCGGGGUUGUGGGGG	bta-miR-2881
ssc-miR-novel-933	2	UGCUCUGCAGUGCCCCACCUUG	hsa-miR-3194-3p
ssc-miR-novel-934	2	UCACUCCUCUCCUCCCGUCU	bta-miR-483
ssc-miR-novel-935	17	AGGACAGGUGGCAGGGAG	bta-miR-10162-5p
ssc-miR-novel-936	1	AGGUUGGGGGUUCGAUUCC	hsa-miR-92a-1-5p
ssc-miR-novel-937	2	CGAGGCAGCGCUGAGGAUGAAC	hsa-miR-1827
ssc-miR-novel-938	2	GGAGGCAGCAGUGGGGGA	hsa-miR-1827
ssc-miR-novel-939	15	UUGCCACCGUGUCCCCCCAG	hsa-miR-4715-3p
ssc-miR-novel-940	18	CAGCCCCAGAGCCCCCUAG	hsa-miR-4323
ssc-miR-novel-941	18	GGAGGAGGAAGGUGUGGAG	eca-miR-8947
ssc-miR-novel-942	3	CCCCCUGGCCUCCUCUUC	bta-miR-331-3p
ssc-miR-novel-943	15	CCAGCAGGGCCAGGGCAG	bta-miR-214
ssc-miR-novel-944	2	UGGACUGGGGGGGGGAAG	hsa-miR-4515
ssc-miR-novel-945	6	CCCCAGGGAAGGCUGUGUCUC	hsa-miR-1915-3p
ssc-miR-novel-946	18	CUCUGAGGGAGCCAGGCC	hsa-miR-12119
ssc-miR-novel-947	3	CUGGGAGGCUGGCAGGGCUCUGGG	bta-miR-30b-3p
ssc-miR-novel-948	17	AAGGAGGGCGUGGUGUAC	bta-miR-10180-3p
ssc-miR-novel-949	18	CUGGGCUGUAUCCCACACC	hsa-miR-3620-5p
ssc-miR-novel-950	13	CUGUGCCUGGUGCAGCCC	hsa-miR-550b-2-5p
ssc-miR-novel-951	4	CUGUGCGGCUCUCUGAGCAGACCC	bta-miR-147
ssc-miR-novel-952	6	CCAGGCACUUCUCUUCUGAACAG	bta-miR-6715
ssc-miR-novel-953	11	CCUGAGGGAAAGGUGAAAA	eca-miR-9077
ssc-miR-novel-954	13	GGGGGCAGGAAUCUGCAUUUCA	eca-miR-486-3p

ssc-miR-novel-955	X	GUCUGCUGGUGGGUGGGC	hsa-miR-6071
ssc-miR-novel-956	12	AGGCAGAUCUAGGGGUGA	bta-miR-2896
ssc-miR-novel-957	7	AAGGGGAGGGAGGGACAG	bta-miR-2343
ssc-miR-novel-958	AEMK02000682.1	UAGGCCCGGCGGAUCUCG	hsa-miR-4747-3p
ssc-miR-novel-959	6	CAAGCUGGGAGCUCUGGCAGC	hsa-miR-320e
ssc-miR-novel-960	7	CUGGGGCUGCAGAAUCAG	bta-miR-2374
ssc-miR-novel-961	6	CACGUGGAUGACAAGGGAC	bta-miR-6527
ssc-miR-novel-962	6	AUCCCACUCCUGACAUCA	hsa-miR-3156-3p
ssc-miR-novel-963	16	UUUGGUGAAUGUCAGCAGG	-
ssc-miR-novel-964	6	GGGAAGCAGGAGGGACCC	hsa-miR-6828-5p
ssc-miR-novel-965	3	GCAUUGUGUCUAGUUUCCUGGGGGU	hsa-miR-4289
ssc-miR-novel-966	12	GGGAGGUGGAGGUGAGGGC	hsa-miR-3689d
ssc-miR-novel-967	1	AGGGGUGGGGGUUCAAGUCC	hsa-miR-608
ssc-miR-novel-968	5	UCUGAUGAUUAUGGAGAA	bta-miR-2471-3p
ssc-miR-novel-969	2	CAGCCCUGGGGCGGGGCCUGUC	bta-miR-2285as
ssc-miR-novel-970	9	GGGAGGGAAGCGGCUGGG	hsa-miR-1249-5p
ssc-miR-novel-971	3	CCCUGGGGGAAGAGGACUGGU	eca-miR-8970
ssc-miR-novel-972	4	CAGCAGUGAGACAGUGGG	eca-miR-9177
ssc-miR-novel-973	2	UGUGGCCCGAGUUGGGCUGGGCC	hsa-miR-663b
ssc-miR-novel-974	12	CUCCAUGCCCUGACCCCC	hsa-miR-5581-3p
ssc-miR-novel-975	3	GGGGUUGUGCCUGGUUACG	hsa-miR-12130
ssc-miR-novel-976	18	CUGGGAACUUCCAUAUGCC	eca-miR-450b-3p
ssc-miR-novel-977	7	GGGCGGCGGCGGCGGCGGC	hsa-miR-4467
ssc-miR-novel-978	12	CCACCGUCGGUUCUCUGGUGUCC	-
ssc-miR-novel-979	11	CGGCCCGGCGGGACUGCGGGAGC	hsa-miR-1538
ssc-miR-novel-980	3	CCUGUCUGAAGAUGGGGGG	hsa-miR-3120-5p
ssc-miR-novel-981	2	CUGAUGGAAGGACACUGAAC	hsa-miR-10395-5p
ssc-miR-novel-982	6	AAACAUCUGAGUAGCUGC	bta-miR-2483-3p
ssc-miR-novel-983	12	AGGUUGGGGGUUCGAAUCC	hsa-miR-92a-1-5p

ssc-miR-novel-984	X	CUGUGGCUGUGGUGUAGG	hsa-miR-4474-3p
ssc-miR-novel-985	13	CACUGUACUCCAGCCUGGGC	bta-miR-2384
ssc-miR-novel-986	14	GCACUGGAGGGCUGAACCC	bta-miR-2339
ssc-miR-novel-987	10	GGGGCCUCAGCUCAGAUGGGAGA	eca-miR-9114
ssc-miR-novel-988	13	UUGAGGAUGGAGUGACGU	bta-miR-2322-3p
ssc-miR-novel-989	X	UGUGACUGACACUGAGGC	bta-miR-134
ssc-miR-novel-990	6	CUGGGGCUGAAGCAGGUU	bta-miR-2374
ssc-miR-novel-991	11	GUGGGAACUCAUGAGAGAA	eca-miR-450b-3p
ssc-miR-novel-992	16	AUGGAGAAUGCCUUGGAAU	hsa-miR-4531
ssc-miR-novel-993	AEMK02000654.1	GUCUCGAGUCGCCCGGGA	hsa-miR-516a-5p
ssc-miR-novel-994	13	AUUCGGACUGGUGUGAAG	-
ssc-miR-novel-995	16	CCUGCAGGGCCGCCUUGGCCUCC	bta-miR-10179-5p
ssc-miR-novel-996	13	AGAGGAAUUGGCUGGGAAGC	bta-miR-11987
ssc-miR-novel-997	11	CUGAGGGAAAGGUGAAAAGC	hsa-miR-6834-5p
ssc-miR-novel-998	2	AGCGGUGCGGCGCGGGCGGGCC	hsa-miR-4746-3p
ssc-miR-novel-999	15	GUGGAUGCCUUGGCAAUCA	cfa-miR-8795
ssc-miR-novel-1000	3	GCUGGAAAUAGCUGGCUC	hsa-miR-875-3p
ssc-miR-novel-1001	15	GUUGGAUUGUUCACCCAC	hsa-miR-5004-3p
ssc-miR-novel-1002	7	AUGAGGUGAGGGUAGGAG	bta-miR-2451
ssc-miR-novel-1003	6	GUCUGUGACCAGAGACUGGGC	bta-miR-2317
ssc-miR-novel-1004	7	GCUGGCCGAGGCGCCGCCA	-
ssc-miR-novel-1005	17	AGGUGGGAGACUGGGAAGG	-
ssc-miR-novel-1006	16	CCUCCCUGGCGUCGGCAUCACC	hsa-miR-7113-3p
ssc-miR-novel-1007	13	GCUGGCCGAGGCGCCGCCA	-
ssc-miR-novel-1008	3	CUUGGAGGUAGAGCCCUG	-
ssc-miR-novel-1009	5	GCGGGGGUGGCGGCGGGGG	hsa-miR-4787-5p
ssc-miR-novel-1010	3	GCAGGGCGAAUGCCUUGGG	-
ssc-miR-novel-1011	15	CCUGUCCAGGGUGAAGGUGC	-
ssc-miR-novel-1012	2	UGAGAUGAGAGCCCUUGGCCA	bta-miR-143

ssc-miR-novel-1013	6	CUUCGAGGAUGCGGAUGUCA	-
ssc-miR-novel-1014	7	AGGUCAGGGGUUCAAGUCC	-
ssc-miR-novel-1015	12	GUUGUCACGCCAGUGGCC	-
ssc-miR-novel-1016	7	AACCUGCCCUUCGGAGGGG	-
ssc-miR-novel-1017	9	GUUGCUGCCGUGGCAGGG	-
ssc-miR-novel-1018	2	AGGGGCUGGGGGUCGGGGG	hsa-miR-185-3p
ssc-miR-novel-1019	6	UCGUGGAUUUGGAGAAUAGACU	bta-miR-3956
ssc-miR-novel-1020	1	AGCUAUGUAGGGAAGGGA	-
ssc-miR-novel-1021	3	UUGUUCACCCACUAAUAGG	-
ssc-miR-novel-1022	18	GGAGAAGCCGGCGGGAGC	bta-miR-2486-5p
ssc-miR-novel-1023	7	CUGGUCUGUGGAGCACUGGC	bta-miR-2357
ssc-miR-novel-1024	2	UAUAGCUCUGGACAGAGAAGGUGC	hsa-miR-7705
ssc-miR-novel-1025	AEMK02000489.1	GAGGGGCUCAGGUGGACCC	eca-miR-185
ssc-miR-novel-1026	18	AUGAUGCGGGUUCGAUUCC	-
ssc-miR-novel-1027	17	AGGAGGACGCUGCCGAAGG	hsa-miR-7154-3p
ssc-miR-novel-1028	9	GGUGCGUCCAGGACUGUGGACGGG	-
ssc-miR-novel-1029	1	CCUGGACUGGAGUACACA	bta-miR-378
ssc-miR-novel-1030	7	GGUGGCUGUAGCUCAGUC	-
ssc-miR-novel-1031	6	GUUCAAAUCCUGCCUCCG	hsa-miR-607
ssc-miR-novel-1032	18	UGCCGCCCACGGUGGCUGCUGC	hsa-miR-4632-3p
ssc-miR-novel-1033	5	AAGGGCUGACACCUGCCC	hsa-miR-4446-3p
ssc-miR-novel-1034	15	AGAAAUGGCCCAAGCGAC	-
ssc-miR-novel-1035	8	UAAAGGAUGUGGGGUUGC	-
ssc-miR-novel-1036	4	GUUCGAAUCCCUGUGGGGA	-
ssc-miR-novel-1037	12	CUGGUCUAGUGGUUAGGAUUUGG	-
ssc-miR-novel-1038	11	GUUCCCAGGCUAGGGGUC	-
ssc-miR-novel-1039	14	GAGAUGGUCAGGUAGAUAGG	-
ssc-miR-novel-1040	4	GGCGAGUUCGAUUCCUGCC	-
ssc-miR-novel-1041	2	CCUGGUCUGGACUUUGGAGGUGAAC	hsa-miR-3663-5p

ssc-miR-novel-1042	14	AGCCAGUGUCUGUGGAGC	eca-miR-9107
ssc-miR-novel-1043	15	AGGGCUGGUCCGGGACGUC	eca-miR-8953
ssc-miR-novel-1044	13	AAGGAGCUGAGGACGGAGAA	bta-miR-28
ssc-miR-novel-1045	X	CCGGCUCUCACCAUUCUCCCCACCC	hsa-miR-6741-3p
ssc-miR-novel-1046	12	CCUCAGGAGAGUUGGGGC	hsa-miR-3664-3p
ssc-miR-novel-1047	11	AUGUUGGGCUGAGAACCUGAGCUGC	bta-miR-2434
ssc-miR-novel-1048	6	GGGACAGUGUCUGGUGGG	cfa-miR-8801
ssc-miR-novel-1049	1	CAGAAGGGGAGUCGGAGCA	bta-miR-3154
ssc-miR-novel-1050	17	CUAAGGGAUCCUGAGCAA	hsa-miR-6892-5p
ssc-miR-novel-1051	5	UGCCAUCCUUAAGUUGGGC	-
ssc-miR-novel-1052	11	GAGUUGGGCUGGGGCGGC	hsa-miR-4259
ssc-miR-novel-1053	6	CCACUCUGGUCGCUCUGGA	bta-miR-2461-5p
ssc-miR-novel-1054	6	UGGGGACUGUGACCAUGGGGAC	hsa-miR-4652-5p
ssc-miR-novel-1055	1	AUUGUGGUUCUGGAGGUCC	-
ssc-miR-novel-1056	6	AGGCUUUCUCCACCCGAG	-
ssc-miR-novel-1057	14	CACCCUGAUGCAGCCAUGCC	-
ssc-miR-novel-1058	6	CCUGGCUGGCUAGGAGCUC	hsa-miR-3064-5p
ssc-miR-novel-1059	6	CUUUCUGUUGAAGGUAGGAUC	bta-miR-2399-5p
ssc-miR-novel-1060	5	GGGUGGGGGUCGAGGGAGG	hsa-miR-92a-2-5p
ssc-miR-novel-1061	5	CUGGGACAUCUGAGUUUGGACCC	eca-miR-1302
ssc-miR-novel-1062	17	GGGCUGUGUCUGCCUCUGGAAGAUC	hsa-miR-7974
ssc-miR-novel-1063	3	CAAGGUGCUGGCGGUGGAGGGC	bta-miR-18b
ssc-miR-novel-1064	9	AGGCAGGGCUGCUGAGGG	hsa-miR-1207-5p
ssc-miR-novel-1065	17	GAGACUUGAAAGGGGCUGC	bta-miR-6531
ssc-miR-novel-1066	8	CGAGUCGGCACACAGUAGGCUCC	hsa-miR-4785
ssc-miR-novel-1067	13	CCCGUGGCCACUUUCCCC	hsa-miR-132-5p
ssc-miR-novel-1068	1	GUUGGGGACUGGGGGUGAUCC	hsa-miR-450a-2-3p
ssc-miR-novel-1069	1	GAUGUGAAAGCCCUGGGC	-
ssc-miR-novel-1070	2	GGGCAGGUUCGGUCGUGGCUGGGG	hsa-miR-4736

ssc-miR-novel-1071	2	CGCCCUUCGGCCACCGGCUG	hsa-miR-6886-3p
ssc-miR-novel-1072	5	AGGCGGAGCUGCUGACACUCGC	hsa-miR-25-5p
ssc-miR-novel-1073	18	AGUGGAAGCAUGGUGACA	hsa-miR-3190-3p
ssc-miR-novel-1074	2	CCACCCUCAUCGCUGCCUGUG	hsa-miR-6865-3p
ssc-miR-novel-1075	14	AGACUGAGAAGCAUGGCA	hsa-miR-6857-3p
ssc-miR-novel-1076	12	CUGGGGGCGGGCUGUGGGG	bta-miR-2454-5p
ssc-miR-novel-1077	15	CCCCCACUUUGGUCCUGUUGGUACC	bta-miR-4286
ssc-miR-novel-1078	6	UGGCUGGGUCUGCAGAAC	eca-miR-8943
ssc-miR-novel-1079	12	AGGCUUGGGGCUGGGGACGAGACUC	eca-miR-9025
ssc-miR-novel-1080	7	CUGGCUCUGCUCUGAGCU	bta-miR-12004
ssc-miR-novel-1081	9	CCAGCAGGAUUGGGGAUUUGGAGCA	bta-miR-214
ssc-miR-novel-1082	17	CUUCAAGCUUUUGGACUGAGAC	hsa-miR-498-5p
ssc-miR-novel-1083	3	AGGAGGAGGAGGAGGAGG	bta-miR-10176-5p
ssc-miR-novel-1084	5	CUGCAGAGGACUGGGAUGCACAC	hsa-miR-3678-3p
ssc-miR-novel-1085	6	GCCUUCCUUCCAUCCUGCCA	oar-miR-1197-5p
ssc-miR-novel-1086	2	CUGGGAUCAUUGGCACCCUC	eca-miR-769a-3p
ssc-miR-novel-1087	AEMK02000394.1	CAGGUCAGAGCUAGGGGCAGG	eca-miR-8933
ssc-miR-novel-1088	8	CCUGUUCUUGGCAGAAUUCAGUUC	hsa-miR-26b-3p
ssc-miR-novel-1089	X	AUGACCUGGGGCCGGAUCAG	eca-miR-9104
ssc-miR-novel-1090	9	GUGGAGCUCGUACUGGAGGGGG	eca-miR-8944
ssc-miR-novel-1091	2	GGGGCGGGGACAGUGAGGUU	bta-miR-2881
ssc-miR-novel-1092	13	AUCGUGAUGGGGAUAGAUA	eca-miR-9068
ssc-miR-novel-1093	5	ACACCCAGGCUGGGGUGCC	cfa-miR-8901
ssc-miR-novel-1094	13	GGGUGCGGCUGCAGCCUAGGACC	hsa-miR-675-5p
ssc-miR-novel-1095	14	UCUGGCUGCUAUGGCCCCCUC	hsa-miR-3064-5p
ssc-miR-novel-1096	14	GUGGGACUGUCAUUUCUCC	bta-miR-2287
ssc-miR-novel-1097	8	CUGAGGACCUUGUCCUGUCC	bta-miR-1224
ssc-miR-novel-1098	7	GGCAGUGCUGGGUAGCUA	bta-miR-2416
ssc-miR-novel-1099	1	CUGAGCUGUUGCCCUCUUUG	bta-miR-2410

ssc-miR-novel-1100	AEMK02000278.1	CAGGUCAGAGCUAGGGGCAGG	eca-miR-8933
ssc-miR-novel-1101	17	CAAGCAGGGCUGCACGCC	bta-miR-2286
ssc-miR-novel-1102	15	UAUCAAAAACACAGGUAUC	-
ssc-miR-novel-1103	12	AUGAGGUGUGGGUAGGGG	bta-miR-2451
ssc-miR-novel-1104	14	ACUUGGGCACAAGAUGAGC	cfa-miR-8903
ssc-miR-novel-1105	AEMK02000361.1	AAUGGAGCCAGUGUCUGA	-
ssc-miR-novel-1106	7	CCCAGUGUAAGGACUAUCAC	bta-miR-199a-5p
ssc-miR-novel-1107	13	AAGUUCAGGCAUUUGGGCUUUACUC	hsa-miR-5000-5p
ssc-miR-novel-1108	4	CACUGUGUGUCCUAGCUCCCC	cfa-miR-8821
ssc-miR-novel-1109	14	GUUCAAAUCCCGGCUUCUCC	hsa-miR-607
ssc-miR-novel-1110	5	UCUGGAUGAAGGCCUGCUUCUGGGU	eca-miR-8911
ssc-miR-novel-1111	10	CCUGGCCUCGCUCAGUGGC	bta-miR-2439-5p
ssc-miR-novel-1112	13	AGCUGGUAGAGCAACUGAU	hsa-miR-133a-5p
ssc-miR-novel-1113	4	GAGAUGAGAGCCUGAUCC	-
ssc-miR-novel-1114	Y	CUGUGAAGUGGCUGGUGAGG	hsa-miR-3611
ssc-miR-novel-1115	10	CUGGACUGUGGGCUGCAU	bta-miR-7863
ssc-miR-novel-1116	2	GGUGGGGAAGACAGAGGCC	bta-miR-491
ssc-miR-novel-1117	3	CUCGGGGCUCUGUUCCUC	hsa-miR-6727-5p
ssc-miR-novel-1118	1	CUGGCCAUACUGUCUUCUCUGCC	hsa-miR-3187-3p
ssc-miR-novel-1119	7	UGAGAACUCUGCUGAAGGA	bta-miR-146b
ssc-miR-novel-1120	9	CUGACCUGGAUUUAGAACUGCAG	eca-miR-9104
ssc-miR-novel-1121	14	AAGAGUCUCGGGCUGUGUGUCC	hsa-miR-5007-5p
ssc-miR-novel-1122	12	CGAGUCAGCGUCUGAGCC	hsa-miR-5702
ssc-miR-novel-1123	9	CUUGGACUUGGAGGUUAACAC	hsa-miR-4529-3p
ssc-miR-novel-1124	9	AGCCUGAAGUAGUUGGGG	-
ssc-miR-novel-1125	5	GGGACUGUGGGGGGCAGA	bta-miR-1388-5p
ssc-miR-novel-1126	6	CAUGGACUUGGAGAACAGACU	-
ssc-miR-novel-1127	17	GGAUGGCUGUGGGUCCUC	eca-miR-9061
ssc-miR-novel-1128	11	CCUGGACAGGAGCUGUCGA	hsa-miR-4638-3p

ssc-miR-novel-1129	2	UGUCAACAUCGGGGGCCUGGC	bta-miR-505
ssc-miR-novel-1130	3	CAAGCAGCUCUAAUGCCUC	bta-miR-562
ssc-miR-novel-1131	10	AGGCAGGGCUGCUGAGGG	hsa-miR-1207-5p
ssc-miR-novel-1132	13	GUCCUUGAGGGGUUGGGCUUC	-
ssc-miR-novel-1133	14	ACUGGCAGGUAGAGCUGGAUCC	eca-miR-9171
ssc-miR-novel-1134	7	CCCUGAGGCAAUGCACUUGCUCUC	hsa-miR-544b
ssc-miR-novel-1135	5	AGGGGAGGCUACAGGAAGGGC	hsa-miR-6825-5p
ssc-miR-novel-1136	7	CUGGGACAUGUGUGCUCUCAGCGUG	eca-miR-1302
ssc-miR-novel-1137	12	GUCUGGAUGAGCUGGGUC	hsa-miR-4782-5p
ssc-miR-novel-1138	13	GAAAUGGAUUCAGAACUUUGA	hsa-miR-4694-3p
ssc-miR-novel-1139	3	CUUUGGACUACAGUCGGCCUC	hsa-miR-7850-5p
ssc-miR-novel-1140	AEMK02000623.1	GUCUGGAUGAGCUGGGUC	hsa-miR-4782-5p
ssc-miR-novel-1141	7	CCAGACUGGCGGAAUCAGCAUCCUC	bta-miR-759
ssc-miR-novel-1142	AEMK02000540.1	UCCUCCUCAUCUCCACCA	cfa-miR-8827
ssc-miR-novel-1143	Y	CUCUCCACACCAACUCGCCC	eca-miR-9135
ssc-miR-novel-1144	9	UCGUCUAGUGGUUAGGAC	-
ssc-miR-novel-1145	13	GUCCUUGCUGAACUGCUUUGAGG	eca-miR-502-5p
ssc-miR-novel-1146	4	CUGGACAGCUACUUCUGAGCAU	hsa-miR-4520-3p
ssc-miR-novel-1147	AEMK02000569.1	CUCUCCACACCAACUCGCCC	eca-miR-9135
ssc-miR-novel-1148	6	CUCCUGGGCUGUCCUGCC	bta-miR-1343-3p
ssc-miR-novel-1149	15	ACCCUCCUCUCUCCCCAGGAUAC	hsa-miR-4667-3p
ssc-miR-novel-1150	2	GUCUGCCUUGCUCUGGGC	hsa-miR-5001-3p
ssc-miR-novel-1151	11	GCUGUCUGGAGGAUAGAAC	hsa-miR-3120-5p
ssc-miR-novel-1152	1	GAAUUAUCUGGGAAGGUA	-
ssc-miR-novel-1153	15	UAUCUGGUUGAUUCUGCC	-
ssc-miR-novel-1154	16	UGAGGGCUGAGGAGAACCUGGCC	hsa-miR-4721
ssc-miR-novel-1155	9	CUGGACCUGGUGCUGGAC	eca-miR-9043
ssc-miR-novel-1156	12	ACCGUGCAUUGUUCUGAC	hsa-miR-1973
ssc-miR-novel-1157	2	AGGACAGUUAGGUUAGCUCUGGC	cfa-miR-8801

ssc-miR-novel-1158	2	GGGGUUGUGGAACUUGGUACC	hsa-miR-12130
ssc-miR-novel-1159	AEMK02000452.1	AUCAUACAAGGACAAUUUCU	eca-miR-485-3p
ssc-miR-novel-1160	9	CCUCAGGACUGAAGGCCAAGCUAC	hsa-miR-3664-3p
ssc-miR-novel-1161	4	CCCAAGUGCAACUAGCUCC	bta-miR-2285ag-3p
ssc-miR-novel-1162	13	AGUGGGAAGAACUCUUCC	bta-miR-10181-5p
ssc-miR-novel-1163	15	UCAUGGUGGGGUUGGGAAA	-
ssc-miR-novel-1164	6	UCCCUCAGACUGGUUGGAAC	bta-miR-760-5p
ssc-miR-novel-1165	9	GUGUCCAUUUGAAUCAACCCACCCA	hsa-miR-3912-5p
ssc-miR-novel-1166	8	GGAGCCCUUGUGUGUUAG	hsa-miR-1233-3p
ssc-miR-novel-1167	13	AGGAGGUCUGAGAGGAGC	-
ssc-miR-novel-1168	4	AGGGGUGCUGGCCAUGAGAG	eca-miR-342-5p
ssc-miR-novel-1169	9	CUCUUUUCUUUGUGAAGU	-
ssc-miR-novel-1170	5	CUGGAUAGCUCAGUCGGUAGAGC	-
ssc-miR-novel-1171	X	AGGUCACCGGUUCGAUCCC	-
ssc-miR-novel-1172	3	GUGGUCUAGUGGUUAGGAUUC	-
ssc-miR-novel-1173	6	UUCCCAGGGCGAGGCUCAUC	-
ssc-miR-novel-1174	14	GGUUCGUUUCCCCUUAGC	-
ssc-miR-novel-1175	2	GUUCGAUCCCAGCCAGAC	-
ssc-miR-novel-1176	11	UGCACAACAGAGCGAGCCAGC	-
ssc-miR-novel-1177	AEMK02000682.1	AGAGGAGCCGCGACAGAGCC	eca-miR-1597
ssc-miR-novel-1178	9	AAUCACUAAUUCCACUGCCA	eca-miR-34b-3p
ssc-miR-novel-1179	2	CAGCUGGCAGAGCAGCGG	eca-miR-9027
ssc-miR-novel-1180	X	GCUUCUUCCUGUUGAGUA	hsa-miR-4778-3p
ssc-miR-novel-1181	2	UGCUCCGGAAGCUGCAGGAGG	-
ssc-miR-novel-1182	9	CCACCUCCCCUGCAAACG	bta-miR-1306
ssc-miR-novel-1183	9	UAUGGCUUUUCAUUCCUAUG	bta-miR-135a
ssc-miR-novel-1184	13	AGAAGUGGCAGAGAGGCCA	hsa-miR-4795-5p
ssc-miR-novel-1185	9	CACAGCAAGUGUAGACAGGCAG	bta-miR-3120
ssc-miR-novel-1186	AEMK02000489.1	GGGCGGUGGUCGGCCGGC	eca-miR-8954

ssc-miR-novel-1187	5	GCGGGGGUGGCGGCGGGG	hsa-miR-4787-5p
ssc-miR-novel-1188	1	CAGAACUUCUGCUCGCCACAUGUA	-
ssc-miR-novel-1189	1	GGUUCAAGUCCAGCUGGG	-
ssc-miR-novel-1190	4	CUCUGGUGGGUGGGGAUUAGUCUC	-
ssc-miR-novel-1191	2	CUCUGGUGGGUGGGGAUUAGUCUC	-
ssc-miR-novel-1192	2	CCAGAGAUGGGGGAGGGC	hsa-miR-4756-3p
ssc-miR-novel-1193	9	GUUCGAUCCCCUGGCCCC	-
ssc-miR-novel-1194	AEMK02000682.1	GCGGGGGUGGCGGCGGGG	hsa-miR-4787-5p
ssc-miR-novel-1195	8	CCUGGUCUGGCUCUGUGACGC	hsa-miR-3663-5p
ssc-miR-novel-1196	3	ACUGGCAUCGUGAUGGACUCC	hsa-miR-4720-5p
ssc-miR-novel-1197	7	GCGCUGGUGUAGGUUGUAG	-
ssc-miR-novel-1198	6	GUUCAUGUCCCAUCAGGG	-
ssc-miR-novel-1199	X	AAUGGAUUACUCCUGGGC	bta-miR-1246
ssc-miR-novel-1200	AEMK02000695.1	GGGCGGUGGUCGGCCGGC	eca-miR-8954
ssc-miR-novel-1201	2	GCUCUGACUCCCCUCCUCC	hsa-miR-301a-5p
ssc-miR-novel-1202	12	UUUACGUCCUUUCACCUAGU	-
ssc-miR-novel-1203	6	CCCCGGGAGCACGGUGGAUC	bta-miR-12045
ssc-miR-novel-1204	6	GGUUCAAAUCCCUCAUCC	-
ssc-miR-novel-1205	4	CUGGGCUCUGACGGGUGCU	hsa-miR-4674
ssc-miR-novel-1206	12	UCUGACCCGCGCCCCUUCCCGCA	cfa-miR-8853
ssc-miR-novel-1207	6	GGGAAUAUAGCUCAGUGGUAGAG	-
ssc-miR-novel-1208	5	AGGACUCGACUGGUGUGCGGACACU	-
ssc-miR-novel-1209	5	GGGGGUAUAGCUCAGGAGUAGAGC	-
ssc-miR-novel-1210	14	GUGGGGACUAAGUACCCUGGCUC	hsa-miR-6810-5p
ssc-miR-novel-1211	AEMK02000452.1	GUCUGAGCAGAGCUCCAGCCCC	hsa-miR-4303
ssc-miR-novel-1212	9	GAAGAACUCAGAGAUGGG	hsa-miR-4511
ssc-miR-novel-1213	6	AGGCUGUGGGUUCGAGCCCU	hsa-miR-7974
ssc-miR-novel-1214	12	CCUGGGCUGUGUGAAGGGGC	oar-miR-134-3p
ssc-miR-novel-1215	14	UUCAGGGUUGUAGGACUGC	-

ssc-miR-novel-1216	3	GAAAGACUAUCAAAGGAUA	hsa-miR-548u
ssc-miR-novel-1217	7	UGGCUUCUCUGACCCGGCC	hsa-miR-6815-3p
ssc-miR-novel-1218	5	CGGCGGCGGCGGCGACGGC	hsa-miR-4467
ssc-miR-novel-1219	1	GGUUCGAUUCCUGGUCAGG	-
ssc-miR-novel-1220	1	AGUGGGGCUCAGAUUCCUGAC	hsa-miR-3151-5p
ssc-miR-novel-1221	6	UGUGUGUGAGCAUCUGCGUGGAC	hsa-miR-6867-5p
ssc-miR-novel-1222	6	AGAGACAGGCGGCUGUAACUCC	hsa-miR-4524a-3p
ssc-miR-novel-1223	8	GUCUGACCCCUUCCCUCC	hsa-miR-4256
ssc-miR-novel-1224	6	AAGCUGAUGUAUAGUGGC	-
ssc-miR-novel-1225	9	CAGGAGGCAUGCCAGGAGGA	hsa-miR-4695-5p
ssc-miR-novel-1226	17	GGUGAGCCAUUUUACAAGGA	eca-miR-9058a
ssc-miR-novel-1227	13	CCUGGUGGCUCUGGAGAAGGGUGG	hsa-miR-4456
ssc-miR-novel-1228	1	GUUCAGAUCCCAGCGGUGC	hsa-miR-3942-3p
ssc-miR-novel-1229	13	CUGGAGGGAAGCAAGCCUGGGC	hsa-miR-6515-5p
ssc-miR-novel-1230	13	GCCUUCUCUGCCAAGCCCUCC	hsa-miR-320a-5p
ssc-miR-novel-1231	11	AUGGAGGGCUGACUGUAGUUACG	hsa-miR-6515-5p
ssc-miR-novel-1232	13	AAGGCUGGUGUCUCUGAG	bta-miR-940
ssc-miR-novel-1233	15	GAAGAAAUCUGGAAGGAC	bta-miR-1434-3p
ssc-miR-novel-1234	7	UGGGGAAGGGAGAAUGGGCUGG	hsa-miR-4271
ssc-miR-novel-1235	16	AGGACCUGGAGUUCGUGC	hsa-miR-492
ssc-miR-novel-1236	6	AGGAGUUAGGGGAGGGGGUCC	hsa-miR-4653-3p
ssc-miR-novel-1237	15	GGUUCAAAUCCUGCUCCA	-
ssc-miR-novel-1238	18	UUGUGUCAAUAUGCGAUGAUG	eca-miR-592
ssc-miR-novel-1239	6	CCCCUCCUUCCCCGCCGG	hsa-miR-4667-3p
ssc-miR-novel-1240	4	GCUGGGUACCUUUUGUGUGGGC	hsa-miR-6861-5p
ssc-miR-novel-1241	1	UUGAUAAGGGUCCAGAAAUGUUUUG	hsa-miR-3161
ssc-miR-novel-1242	14	CUGGAUGGAAAAGGGGGAC	bta-miR-2392
ssc-miR-novel-1243	13	CAAAAGCAGAUUCUGGGA	hsa-miR-548e-5p
ssc-miR-novel-1244	1	GAAGAACUCAGAGAUGUG	hsa-miR-4511

ssc-miR-novel-1245	6	UCUGAAUGGCUUCUAUUUCAC	bta-miR-2303
ssc-miR-novel-1246	9	GUGGGGCUCGAGGUGUGAUGGG	bta-miR-2374
ssc-miR-novel-1247	17	CUCCUCCACAAGUGCUCC	hsa-miR-4691-5p
ssc-miR-novel-1248	1	CUCUCUCUUCUGGACUCAGCUCU	hsa-miR-6873-3p
ssc-miR-novel-1249	3	GAGGAGGGGGAGGAGGGGGC	bta-miR-10180-3p
ssc-miR-novel-1250	5	CUCCUGGGCUGGAAACCUUUG	bta-miR-1343-3p
ssc-miR-novel-1251	1	CAGGGUCUGUGGCUCUAA	hsa-miR-1182
ssc-miR-novel-1252	8	GUUCUGCCUGCAGGGAGACCG	hsa-miR-6738-3p
