# Supplementary material for: Investigation of salivary microRNAs as non-invasive biomarkers for weaning stress in piglets: A preliminary study
Source: PLoS One. 2026 Oct 13;21(10):e0360727. doi: 10.1371/journal.pone.0360727 (PMC13660905; doi:10.1371/journal.pone.0360727)
Supplement: Supplementary file 3 [file pone.0360727.s004.docx]

**Response to comments**

We appreciate the editor and reviewer’s valuable remarks that helped to improve our manuscript. To address these comments, we have provided point-by-point responses to all comments. In the revised manuscript, the changes related to the reviewer comments and the parts modified to make it readable are highlighted in yellow and light blue, respectively.

**Response to editor’s comments:**

**1. Please ensure that your manuscript meets PLOS ONE's style requirements, including those for file naming. The PLOS ONE style templates can be found at https://journals.plos.org/plosone/s/file?id=wjVg/PLOSOne_formatting_sample_main_body.pdf and https://journals.plos.org/plosone/s/file?id=ba62/PLOSOne_formatting_sample_title_authors_affiliations.pdf.**

**Response:** We thank the journal office for this reminder. We have carefully rechecked the manuscript and confirmed that it follows the PLOS ONE style requirements, including the formatting of the title page, main text, and file naming for revision submission.

**2.Please note that PLOS One has specific guidelines on code sharing for submissions in which author-generated code underpins the findings in the manuscript. In these cases, we expect all author-generated code to be made available without restrictions upon publication of the work. Please review our guidelines at https://journals.plos.org/plosone/s/materials-and-software-sharing#loc-sharing-code and ensure that your code is shared in a way that follows best practice and facilitates reproducibility and reuse.**

**Response:** We have reviewed the PLOS ONE code-sharing guidelines. No custom author-generated code was developed or used in this study. The analyses were conducted using publicly available software and tools, and the relevant software names, versions, and analysis criteria are provided in the Materials and Methods section.

**3. Thank you for stating in your Funding Statement:**

**“This work was supported by the National Research Foundation of Korea (NRF) grant funded by the Korea government (MSIT) (No. RS-2022-00166938 and RS-2026-25477091). “**

**Please provide an amended statement that declares *all* the funding or sources of support (whether external or internal to your organization) received during this study, as detailed online in our guide for authors at http://journals.plos.org/plosone/s/submit-now. Please also include the statement “There was no additional external funding received for this study.” in your updated Funding Statement.**

**Please include your amended Funding Statement within your cover letter. We will change the online submission form on your behalf.**

**Response:** We have amended the Funding Statement and included the requested sentence in the cover letter.

**4. Thank you for stating the following financial disclosure:**

**“This work was supported by the National Research Foundation of Korea (NRF) grant funded by the Korea government (MSIT) (No. RS-2022-00166938 and RS-2026-25477091). “**

**Please state what role the funders took in the study. If the funders had no role, please state: "The funders had no role in study design, data collection and analysis, decision to publish, or preparation of the manuscript."**

**If this statement is not correct you must amend it as needed.**

**Please include this amended Role of Funder statement in your cover letter; we will change the online submission form on your behalf.**

**Response:** We have included the following Role of Funder statement in the cover letter: “The funders had no role in study design, data collection and analysis, decision to publish, or preparation of the manuscript.”

**5. PLOS requires an ORCID iD for the corresponding author in Editorial Manager on papers submitted after December 6th, 2016. Please ensure that you have an ORCID iD and that it is validated in Editorial Manager. To do this, go to ‘Update my Information’ (in the upper left-hand corner of the main menu), and click on the Fetch/Validate link next to the ORCID field. This will take you to the ORCID site and allow you to create a new iD or authenticate a pre-existing iD in Editorial Manager.**

**Response:** The corresponding author’s ORCID iD has been verified in Editorial Manager.

**6. If the reviewer comments include a recommendation to cite specific previously published works, please review and evaluate these publications to determine whether they are relevant and should be cited. There is no requirement to cite these works unless the editor has indicated otherwise.**

**Response:** The reviewer did not request citation of specific previously published works.

**Response to reviewer’s comments:**

**1. The present paper showed an interesting overview on the salivary microRNAs and their association with the weaning transition in piglets. My major comment is related to the small number of animals: to assess the potential of microRNAs as weaning biomarkers, a large cohort of animals must be included in this study. Perhaps will be better to classify this study as a preliminary one, and to enlarge the number of animals.**

**Response:** Thank you for your positive evaluation and valuable comment. We agree that the relatively small number of animals is a limitation of this study. According to your comment, we have revised the Conclusion to clarify that this study is preliminary and that validation using larger cohorts is required to confirm the biomarker potential of salivary miRNAs for weaning stress (Lines 4, 31-33, 75-77, 371, 374-377, 381-384).

**2. Also, in Materials and methods section are no indication on the statistical analysis tools used.**

**Response:** Thank you for pointing this out. We added detailed information on the statistical analysis tools used for "Differentially expressed miRNA analysis" in the Materials and Methods section (Lines 110-111, 113).

**3. I did not understand the feasibility of this miRNAs as biomarkers, taken into account that weaning is a transitory period in piglets. I agree that saliva could be a critical sample for weaning piglets, but the miRNAs analysis seems to be too expensive for a quick assessment.**

**So, my recommendation is a major revision, with a careful assessment of the usefulness of miRNA detection in the saliva taking into account the transitory features of weaning stress in piglets.**

**Response:** Thank you for your comment. We agree that the feasibility of salivary miRNAs as biomarkers should be carefully interpreted considering the transitory nature of weaning stress and the cost of miRNA analysis. According to your comment, we have revised the manuscript to avoid overstatement and clarified that salivary miRNAs should be considered candidate molecular indicators rather than immediately applicable diagnostic biomarkers (Lines 34-35, 359-360). We also have revised the Conclusion to emphasize the need for further validation before practical application (Lines 381-384).
